# Global, regional, and national burden of neglected tropical diseases and malaria, 1990–2021

**DOI:** 10.1265/ehpm.25-00038

**Published:** 2025-07-17

**Authors:** Talaiti Tuergan, Aimitaji Abulaiti, Alimu Tulahong, Ruiqing Zhang, Yingmei Shao, Tuerganaili Aji

**Affiliations:** 1 Hepatobiliary and Echinococcosis Surgery Department, Digestive and Vascular Surgery Center, First Affiliated Hospital of Xinjiang Medical University, Urumqi, China; 2 State Key Laboratory of Pathogenesis, Prevention and Management of High Incidence Diseases in Central Asia, Xinjiang Medical University, Urumqi, China

**Keywords:** Neglected tropical diseases and malaria, Burden of disease, GBD, 1990, 2021

## Abstract

**Background:**

Neglected tropical diseases (NTDs) and malaria pose a major health challenge, especially in low- and middle-income countries.

**Methods:**

Initially, we performed a descriptive analysis of the Global Burden of Disease (GBD) 2021 database, categorizing data by subtypes. Next, linear regression models were employed to analyze temporal trends. We then utilized four predictive models to forecast the future burden. Additionally, we explored the relationship between estimated annual percentage change (EAPCs) and age-standardized rates (ASRs), as well as Human Development Index (HDI) scores for 2021. Furthermore, decomposition analysis was applied to assess the influence of aging, population dynamics, and epidemiological changes. Lastly, frontier analysis was conducted to examine the connection between disease burden and sociodemographic development.

**Results:**

In 2021, NTDs and malaria contributed significantly to the global disease burden, with considerable disparities across genders, age groups, Socio-demographic Index (SDI) regions, GBD regions, and individual countries. From 1990 to 2021, both the number of cases and the associated ASRs have shown a recent downward trend. The EAPCs are positively correlated with ASRs and HDI scores. Projections indicate a continued decline in disease burden through 2046. Additionally, our decomposition analysis highlighted the positive impact of aging and epidemiological shifts on the reduction of the disease burden. Finally, frontier analysis revealed that countries and regions with higher SDI scores have greater potential for further reducing their health burden.

**Conclusion:**

While the global burden of NTDs and malaria has improved overall, significant disparities remain across regions and countries. Our findings highlight the importance of implementing targeted intervention strategies and maintaining sustained investments to tackle the ongoing challenges.

**Supplementary information:**

The online version contains supplementary material available at https://doi.org/10.1265/ehpm.25-00038.

## 1. Introduction

Neglected Tropical Diseases (NTDs) and malaria pose a substantial and frequently underestimated public health challenge worldwide, especially in tropical and subtropical areas [1]. These diseases are predominantly caused by parasites, viruses, bacteria, and fungi, and they affect the most vulnerable and impoverished populations [2]. The extensive list of NTDs includes diseases such as lymphatic filariasis, schistosomiasis, visceral leishmaniasis, onchocerciasis, Chagas disease, dengue, and malaria, among others [3]. Despite their profound impact on human health, economic development, and social well-being, these diseases receive limited attention and funding compared to other more visible health threats [4].

The Global Burden of Disease (GBD) has consistently emphasized the significant loss of disability-adjusted life-years (DALYs) attributed to NTDs and malaria [5]. From 1990 to 2021, the global landscape of these diseases has undergone significant changes, with varying trends observed across regions and countries. This study intends to deliver a comprehensive examination of the global, regional, and national burdens of NTDs and malaria over the specified period, comparing our findings with existing literature to underscore key trends and disparities.

NTDs are ancient diseases that have plagued human societies for centuries [6]. They are often transmitted through vectors such as mosquitoes, flies, and other insects, or through contact with contaminated soil, water, or food [7]. These diseases not only cause significant morbidity and mortality but also lead to long-term disability and social stigma, further exacerbating the cycle of poverty in affected communities [8]. For instance, lymphatic filariasis, also known as elephantiasis, can cause severe swelling of the limbs and genitalia, leading to chronic disability and social isolation [9].

Malaria, caused by Plasmodium parasites transmitted through the bites of infected Anopheles mosquitoes, remains a leading cause of both death and illness worldwide [10]. While substantial strides have been made in malaria control and elimination, the disease remains a persistent and severe threat, particularly in sub-Saharan Africa [11]. The growing challenge posed by drug-resistant parasites and insecticide-resistant vectors adds another layer of complexity to ongoing control efforts [12].

Numerous studies have assessed the burden of NTDs and malaria at various geographic levels. For instance, a subnational study in Brazil analyzing NTDs from 1990 to 2016 indicated a significant decrease in DALYs attributed to NTDs, with Chagas disease, schistosomiasis, and dengue identified as the primary contributors [13]. However, despite these improvements, NTDs persist as significant and preventable causes of disability and early death, underscoring the need for continued and comprehensive strategies for their control and prevention.

Regionally, the burden of NTDs and malaria varies considerably. In Africa, where the majority of NTDs and malaria cases occur, the diseases are often concentrated in rural and remote areas with limited access to health care and sanitation facilities [14]. The situation is exacerbated by climate change, which is altering vector ecology and disease transmission patterns [15]. In Asia, where NTDs such as lymphatic filariasis, schistosomiasis, and soil-transmitted helminthiasis are endemic, progress has been made in disease control through mass drug administration and improved sanitation [16].

Despite these efforts, the control and elimination of NTDs and malaria remain challenging due to various factors, including limited resources, poor infrastructure, and social and cultural barriers [17]. Moreover, the COVID-19 pandemic has disrupted health systems and disease control programs, further threatening progress in NTD and malaria control [18].

In this study, we examine the global, regional, and national burden of NTDs and malaria between 1990 and 2021, utilizing data from the GBD study. We aim to identify key trends, disparities, and determinants of the burden of these diseases and provide insights into the effectiveness of current control strategies. By comparing our findings with existing literature, we aim to contribute to the ongoing debate on the best approaches to control and eliminate NTDs and malaria globally.

## 2. Methods

### 2.1. Data collection

In this research, a robust and multifaceted methodological framework was employed to thoroughly assess and quantify the burden imposed by NTDs as well as malaria. The study integrated a variety of analytical techniques and data sources to generate estimates that reflect the impact of these diseases across different scales. Specifically, the evaluation was conducted on a global scale, while also delving into regional differences and national-level details. Moreover, the investigation spanned a considerable timeframe—from 1990 to 2021—thereby allowing for an in-depth analysis of long-term trends and changes in disease burden over these three decades. All data for NTDs and malaria were extracted from the GBD 2021 public repository (http://ghdx.healthdata.org/gbd-2021), a large-scale initiative aimed at quantifying health losses caused by major diseases, injuries, and risk factors. The GBD database serves as a comprehensive resource that provides estimates for a broad spectrum of epidemiological metrics. These estimates are applied to a variety of health conditions, including NTDs and malaria, offering valuable insights into their impact [19]. The GBD 2021 study represents a substantial collaborative endeavor, incorporating data from diverse sources such as vital registration systems, household surveys, population censuses, and peer-reviewed studies [20]. The GBD 2021 framework utilizes a harmonized dataset and standardized methodologies to ensure comparability and consistency across diverse populations and time periods.

### 2.2. Study outcomes and explanatory variables

The main study outcomes in this research were the burden of NTDs and malaria, measured by multiple key epidemiological metrics obtained from the GBD 2021 public repository. These outcomes included the number of incidence cases, number of prevalence cases, number of deaths cases, number of DALYs cases, as well as age-standardized incidence rate (ASIR), age-standardized prevalence rate (ASPR), age-standardized deaths rate (ASDR), and age-standardized DALYs rate (ASDAR).

The ASIR is defined as the number of new cases of a disease occurring in a population per 100,000 person-years, adjusted for age differences across populations. This adjustment allows for a more comparable measure of disease occurrence over time and between different regions. The ASPR represents the total number of existing cases of a disease in a population per 100,000 persons, also adjusted for age, providing an indication of the overall disease burden at a given time. The ASDR is calculated as the number of deaths due to a disease per 100,000 person-years, age-adjusted, which helps in comparing mortality risks across populations with varying age structures. The ASDAR measures the number of DALYs lost due to a disease per 100,000 person-years, age-adjusted. DALYs combine years of life lost due to premature death and years lived with disability, and the age-standardized rate enables a standardized assessment of the overall disease burden considering age variations.

Explanatory variables (covariates) considered in this study included sex, age, Socio-demographic Index (SDI) regions, GBD regions, individual countries, and Human Development Index (HDI) scores. The SDI is a composite measure that reflects a region’s average income per capita, educational attainment, and fertility rate The HDI is a summary measure of average achievement in key dimensions of human development: a long and healthy life, being knowledgeable, and having a decent standard of living.

Data for NTDs and malaria were sourced from the GBD 2021 database, which integrates data from various channels. As reported by the GBD 2021 study, the data compilation involves information from vital registration systems, household surveys, population censuses, and peer-reviewed studies. In addition to the GBD database, no other external data sources were used in this study.

### 2.3. Description analysis

This study employs a mixed-methods design. Firstly, we conducted a descriptive analysis utilizing the GBD 2021 database [20], with a specific emphasis on the year 2021. In our analysis, we carefully partitioned the dataset into several distinct subgroups to facilitate a more detailed examination. Specifically, we categorized the information by factors such as sex and age, as well as by geographical delineations including SDI regions, GBD regions, and individual countries. This approach, consistent with standard GBD methodology [21], enabled us to assess patterns and disparities across different demographic and regional segments. For each subgroup, we performed a thorough analysis that encompassed health indicators—namely, incidence, prevalence, deaths, and DALYs. This meticulous evaluation, guided by epidemiological stratification principles [22], enabled us to uncover significant disparities in the burden of NTDs and malaria across various demographic and geographic categories.

### 2.4. Trend analysis

Secondly, we investigated the evolution of the NTDs and malaria burden throughout the study period (1990–2021). To quantify these temporal trends, we employed linear regression models to determine the estimated annual percentage change (EAPC) for every global disease burden indicator and subgroup [23]. Positive EAPC values denoted an upward trend, while negative values indicated a decline. Additionally, to characterize regional trends, we conducted cluster analysis using EAPC values, categorizing GBD regions into four clusters based on similarity in burden trajectories. This approach follows established methodologies for trend analysis in public health research [23].

### 2.5. Predicted analysis

To forecast future disease burden, we utilized the age-period-cohort (APC) model, which disentangles the effects of age, period, and birth cohort on observed trends [24]. Sensitivity analyses were performed using two alternative methods: the Autoregressive Integrated Moving Average (ARIMA) model [25] and exponential smoothing (ES) model [26], to validate projection robustness. These time-series forecasting techniques are widely used in epidemiological modeling [27].

### 2.6. Correlation analysis

To investigate associations between EAPCs and socioeconomic factors, we used Spearman correlation analysis, a non-parametric method suitable for non-normally distributed data [HDI scores, age-standardized rates (ASRs)] [28].

### 2.7. Decomposition analysis

A decomposition analysis was conducted to quantify the contributions of demographic aging, population dynamics, and epidemiological transitions to disease burden changes (1990–2021). This method, adapted from previous studies on health trend attribution [29], isolates the effects of each factor to clarify their individual roles in driving burden variations.

### 2.8. Frontier analysis

To delve deeper into the association between the burden of NTDs and malaria and the level of sociodemographic progress, we conducted a frontier analysis. This approach allowed us to establish a non-linear frontier that delineates the minimum achievable burden at each level of development. Essentially, this frontier serves as a benchmark, reflecting the optimal disease burden that could be attained under ideal conditions given a country’s socioeconomic status. By comparing actual burden estimates against this benchmark, we gained valuable insights into the efficiency of health systems and identified areas where improvements could potentially lead to substantial reductions in disease impact. Non-parametric data envelope analysis, based on methodologies outlined in previous studies [10, 11], was employed to construct this frontier. The effective difference was defined as the gap between the observed rate in a country and the minimum achievable rate suggested by its corresponding frontier. This metric captures the unrealized health potential within the current developmental framework of the nation or region. In other words, it quantifies the extent to which a country’s actual health outcomes deviate from the optimal scenario that could be reached under ideal conditions for its level of socioeconomic progress. This measure not only highlights areas where health performance may be improved but also serves as an indicator of the latent opportunities for reducing the burden of diseases like NTDs and malaria.

In this study, all statistical analyses, including data processing, modeling, and graphical visualizations, were conducted using the statistical software R, version 3.5.1. R provided a comprehensive and flexible environment that enabled us to perform a wide range of statistical tests and create detailed, high-quality visual representations of our data. This choice of software ensured both the reproducibility and transparency of our analytical procedures while facilitating clear communication of our findings.

## 3. Results

### 3.1. The disease burden of NTDs and malaria in 2021

In 2021, NTDs and malaria caused 309,802,762 incidence [95% uncertainty interval (UI): 232,164,372–398,594,668], with an ASIR of 4,259.54 (95% UI: 3,188.38–5,488.91) per 100,000 population. The prevalence totaled 1,111,497,287 (95% UI: 1,049,604,638–1,189,217,544), corresponding to an ASPR of 14,454.15 (95% UI: 13,659.74–15,452.2). Additionally, 847,472 deaths (95% UI: 375,547–1,617,956) were recorded, with an ASDR of 11.8 (95% UI: 5.22–22.48). The total DALYs reached 71,629,913 (95% UI: 38,735,014–122,926,457), and the corresponding ASR of DALYs was 1,020.27 (95% UI: 542.47–1,756.51) per 100,000 population (Tables S1–S4).

Regarding gender differences, our analysis revealed distinct patterns in the epidemiological metrics of NTDs and malaria. Specifically, the data indicated that females experienced a considerably higher number of incidence cases, as well as an elevated ASIR, when compared to males. Conversely, the prevalence of these diseases and the overall disease burden—as reflected by the ASPR—were more pronounced among males. Furthermore, when examining deaths and DALYs, both the absolute case numbers and the ASRs consistently tended to be higher in males relative to females. These findings are illustrated in Fig. S1 and further detailed in Tables S1–S4.

Analysis of the distribution of disease burden across age groups (refer to Fig. S2 and Tables S1–S4) revealed a clear trend: in 2021, children (<5 years old) experienced a disproportionately high burden of disease compared to other age groups. This indicates that children were particularly vulnerable during that year. This vulnerability can be attributed to multiple factors. Biologically, the immune systems of children are still maturing, resulting in a reduced ability to mount effective immune responses against pathogens. Moreover, environmental and social changes in 2021, such as modifications to public health interventions, may have further exacerbated the exposure risk for this age group. On the other hand, while the elderly population also faces health risks due to age-related physiological decline and comorbidities, their disease burden was relatively lower in 2021. This may be due to protective measures implemented, including vaccination programs, improved care in senior care facilities, and social distancing protocols that minimized their exposure to infectious diseases.

At the regional level, when evaluating data based on the SDI, the relationship between the number of cases and the ASRs displayed a distinct “J-shaped” pattern. In essence, regions with lower SDI levels—typically reflecting poorer socioeconomic conditions—exhibited the highest disease burden. These patterns, as illustrated in Figs. S3–S4 and detailed in Tables S1–S4, highlight the critical impact of sociodemographic factors on health outcomes and underscore the need for targeted public health interventions in low SDI regions.

Across the 54 GBD regions, Africa exhibited the highest numbers of incidence, deaths, and DALYs cases related to NTDs and malaria. Following Africa, the Sub-Saharan Africa - WB and the broader African Region ranked next in terms of disease burden. This ranking underscores the substantial impact of these conditions on the African continent and highlights the urgent need for targeted interventions in these high-burden regions. For prevalence, the Limited Health System category ranked first, followed by World Bank Lower Middle Income and Asia. High-income North America recorded the lowest number of incidence cases, followed by Western North America and Central Europe. Australasia and Commonwealth High Income ranked lowest in prevalence, deaths, and DALYs cases. For ASRs, Western Africa and Western Sub-Saharan Africa led in ASIR, ASDR, and ASDAR, while Central Sub-Saharan Africa ranked highest in ASPR, followed by Central Africa and Minimal Health System. The lowest ASIR was recorded in High-income North America, followed by North America and Western Europe. For ASPR, ASDR, and ASDAR, Australasia ranked the lowest (Fig. S5, Tables S1–S4).

Globally, the burden of NTDs and malaria varied considerably. Nigeria recorded the highest number of incidence, deaths, and DALYs cases, while India led in number of prevalence cases. Conversely, San Marino recorded the lowest number of incidence and prevalence cases, while Tokelau reported the lowest number of deaths and DALYs cases. Regarding ASRs, Liberia recorded the highest ASIR and ASPR, Burkina Faso the highest ASDR, and Sierra Leone the highest ASDAR. The lowest ASIR, ASPR, ASDR, and ASDAR were observed in Ireland, Norway, Finland, and Canada, respectively (Fig. S6, Tables S1–S4).

### 3.2. Temporal trend for the disease burden of NTDs and malaria from 1990 to 2021

**Fig. 1:**
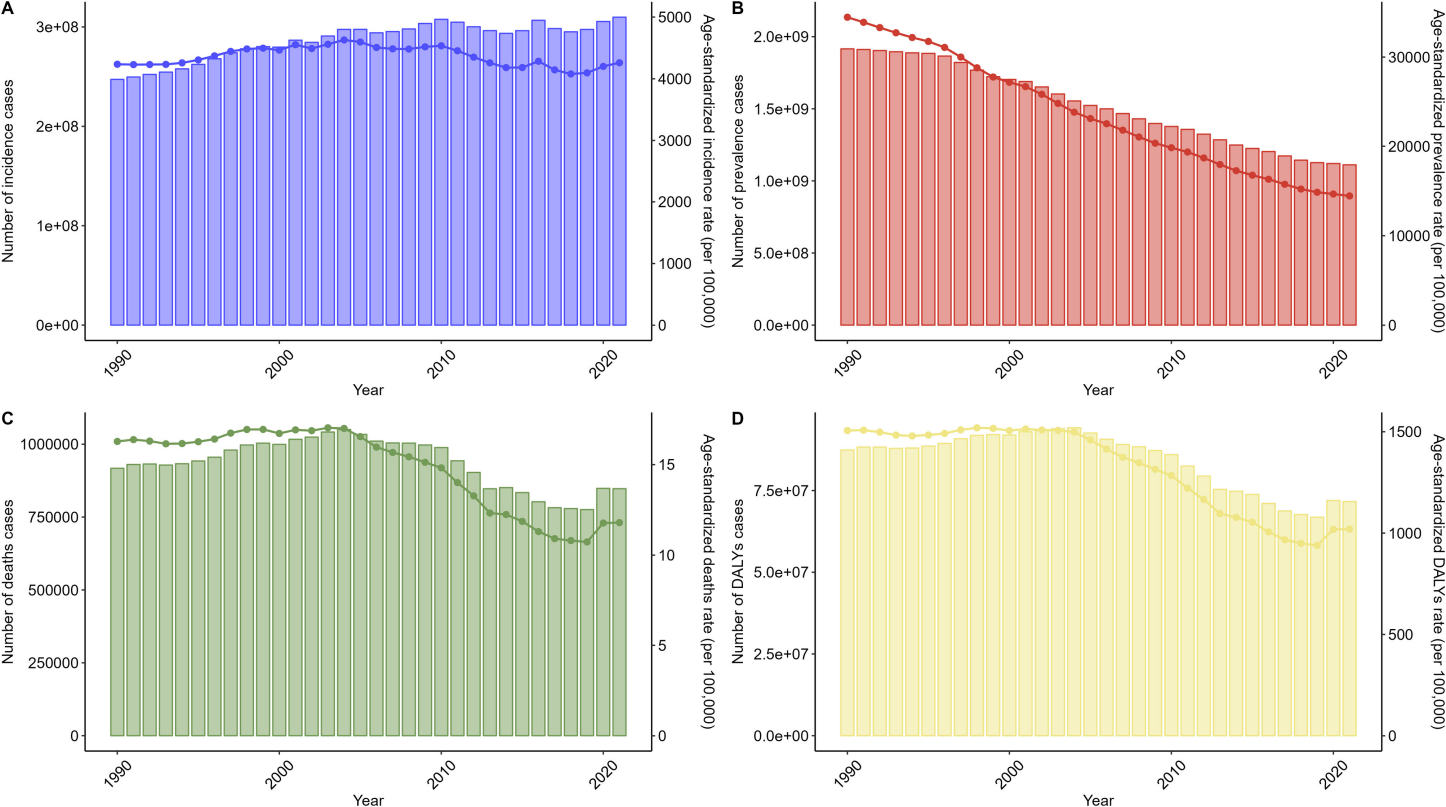
Global Trends in NTD and Malaria Burden, 1990–2021.

From 1990 to 2021, the number of incidence cases for NTDs and malaria exhibited an increasing trend, whereas all other indicators showed a decreasing trend. Incidence rose from 247,235,621 (95% UI: 196,271,497–308,015,155) to 309,802,762 (95% UI: 232,164,372–398,594,668). However, the corresponding ASIR declined slightly from 4,235.42 (95% UI: 3,347.35–5,282.14) to 4,259.54 (95% UI: 3,188.38–5,488.91), with an EAPC of −0.11 (95% CI: −0.24–0.03). Prevalence decreased from 1,915,551,162 (95% UI: 1,816,268,530–2,023,083,395) to 1,111,497,287 (95% UI: 1,049,604,638–1,189,217,544). Deaths increased slightly from 917,420 (95% UI: 558,500–1,685,655) to 847,472 (95% UI: 375,547–1,617,956), while DALYs declined from 87,421,645 (95% UI: 59,036,484–141,599,541) to 71,629,913 (95% UI: 38,735,014–122,926,457). Corresponding ASRs for prevalence, deaths, and DALYs demonstrated significant declines, with EAPC values of −2.98 (95% CI: −3.05 to −2.91), −1.49 (95% CI: −1.80 to −1.17), and −1.66 (95% CI: −1.94 to −1.38), respectively (Fig. 1, Tables S1–S4).

The trends in both the number of cases and ASRs among males and females mirrored those observed in the overall population, with all indicators declining except for the number of incidence cases (Fig. S7, Tables S1–S4). Similar trends were observed in middle-aged groups, but notable differences appeared in children and older adults (Fig. S8, Tables S1–S4).

At the SDI regional level, the ASRs for prevalence, deaths, and DALYs showed a consistent decline across all SDI regions, a pattern that mirrors the overall global trend. In contrast, the trend for the ASIR was more complex: while the low- and low-middle SDI regions maintained a steady and consistent trend, the other three SDI regions experienced a different trajectory, indicating variability in ASIR trends based on socioeconomic development. Additionally, when looking at the absolute numbers, the total cases of deaths and DALYs consistently decreased across all SDI regions. However, the trends in the absolute numbers for incidence and prevalence cases did not follow a uniform pattern and instead exhibited slight variations among the different SDI regions (Fig. S9, Tables S1–S4).

**Fig. 2:**
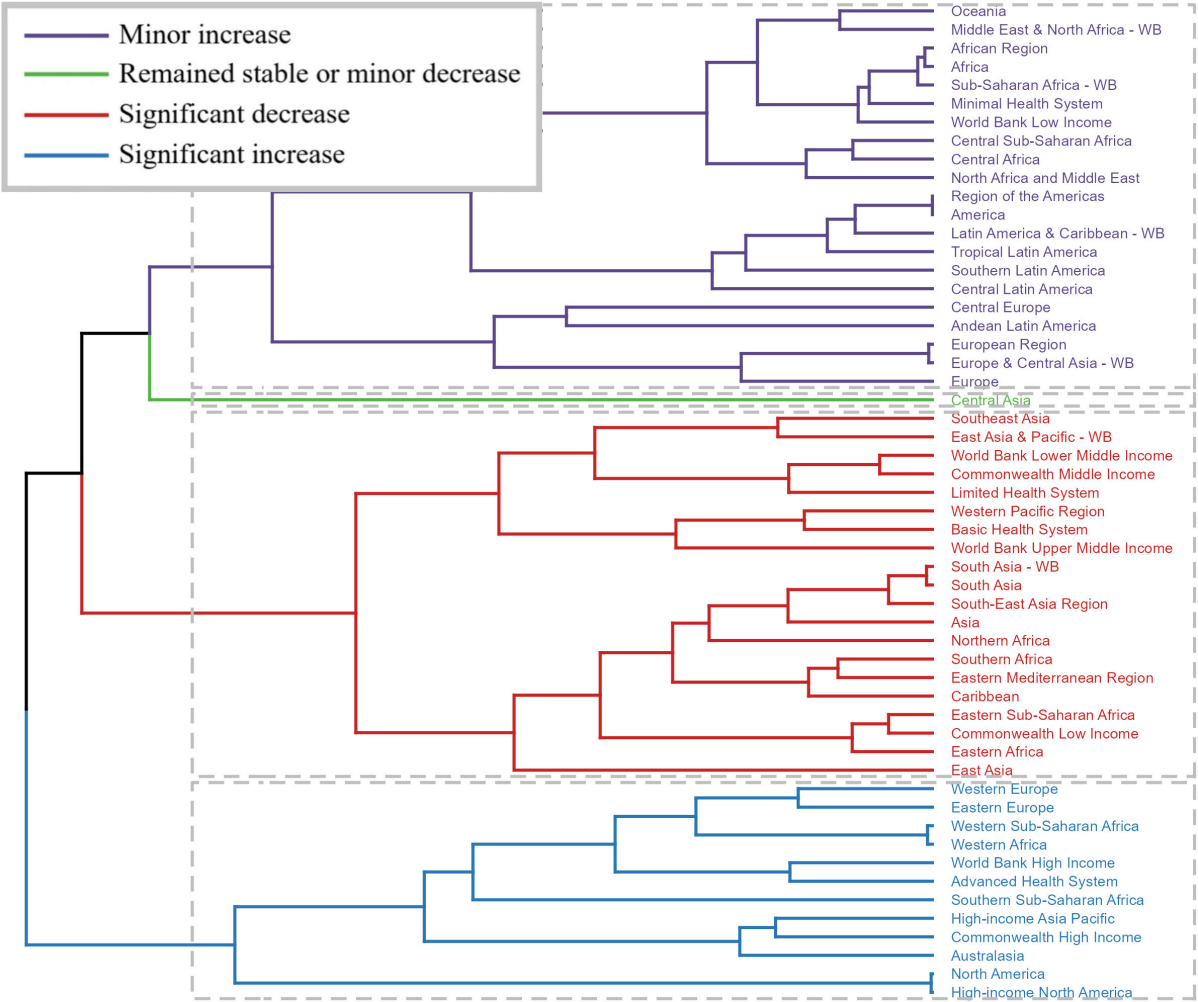
Cluster Analysis of NTD and Malaria Rate Changes (EAPC), 1990–2021.

Among the 54 GBD regions, the burden of NTDs and malaria displayed significant regional differences. Hierarchical clustering analysis revealed distinct patterns: regions such as Western and Eastern Europe, Western and Southern Sub-Saharan Africa, and high-income regions like North America and Australasia exhibited significant increases in ASRs. In contrast, regions including Southeast Asia, South Asia, Northern Africa, and Eastern Sub-Saharan Africa experienced notable decreases in ASRs (Fig. 2, Tables S1–S4).

**Fig. 3:**
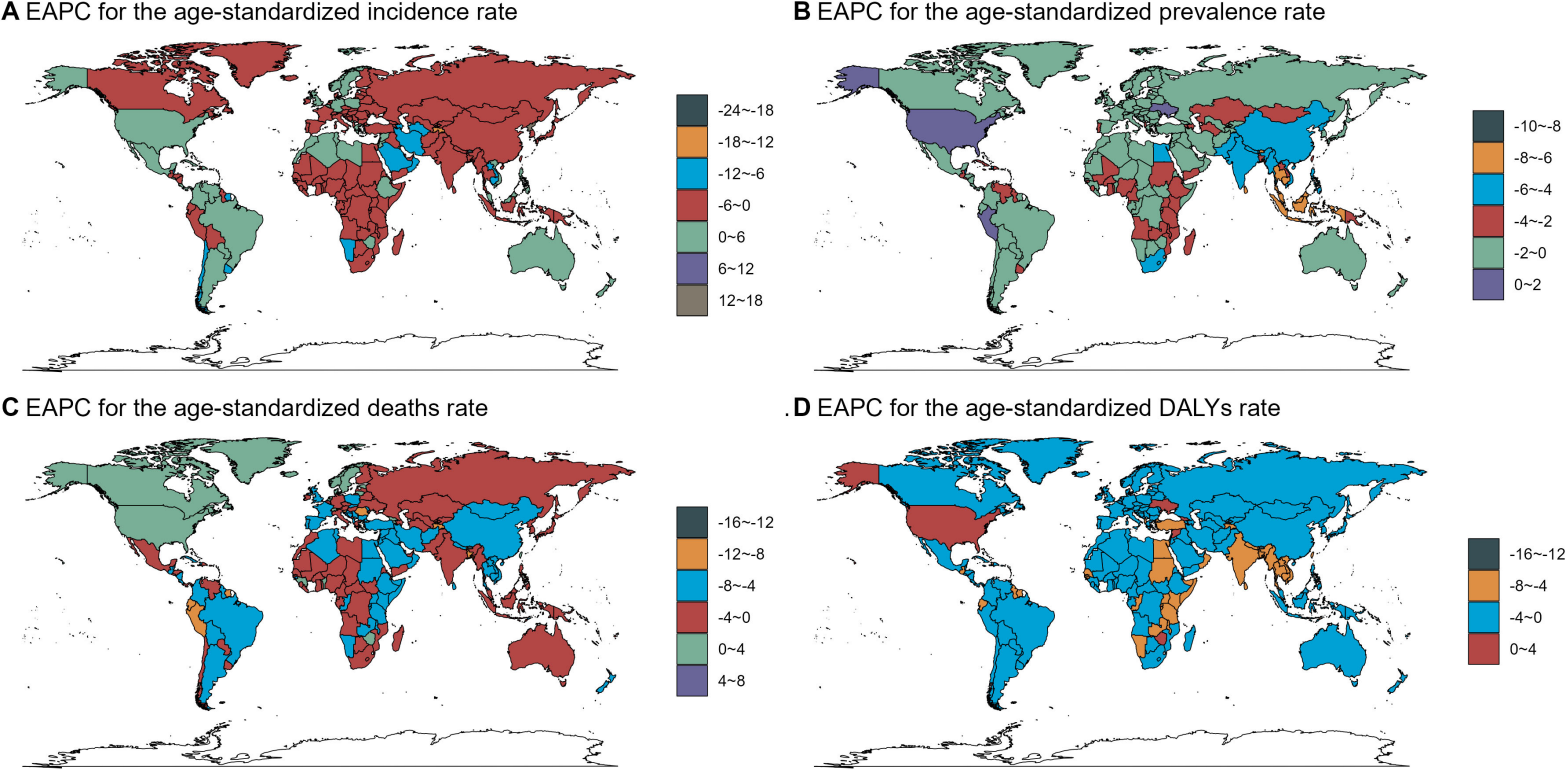
Estimated Annual Percentage Change (EAPC) in NTD and Malaria Burden Rates, 1990–2021.

At the country level, considerable variability in trends was evident. For instance, Eswatini exhibited the most pronounced decrease in the ASIR, with an EAPC of −9.68 [95% confidence interval (CI): −11.23 to −8.11]. In contrast, the Maldives recorded the largest reduction in the ASPR, with an EAPC of −8.62 (95% CI: −9.51 to −7.72). These findings underscore the heterogeneous nature of disease trends across different nations. Ecuador showed the most pronounced decrease in ASDR (EAPC = −8.78, 95% CI: −10.18 to −7.34), and Vanuatu recorded the largest reduction in ASDAR (EAPC = −7.81, 95% CI: −9.70 to −5.88). Conversely, Monaco demonstrated the most significant increases in ASIR (EAPC = 9.8, 95% CI: 7.60–12.05), ASDR (EAPC = 7.80, 95% CI: 5.80–9.85), and ASDAR (EAPC = 2.20, 95% CI: 1.39–3.02), while Peru showed the highest rise in ASPR (EAPC = 1.88, 95% CI: 1.28–2.48) (Fig. 3, Tables S1–S4).

### 3.3. The influential factors for EAPC

**Fig. 4:**
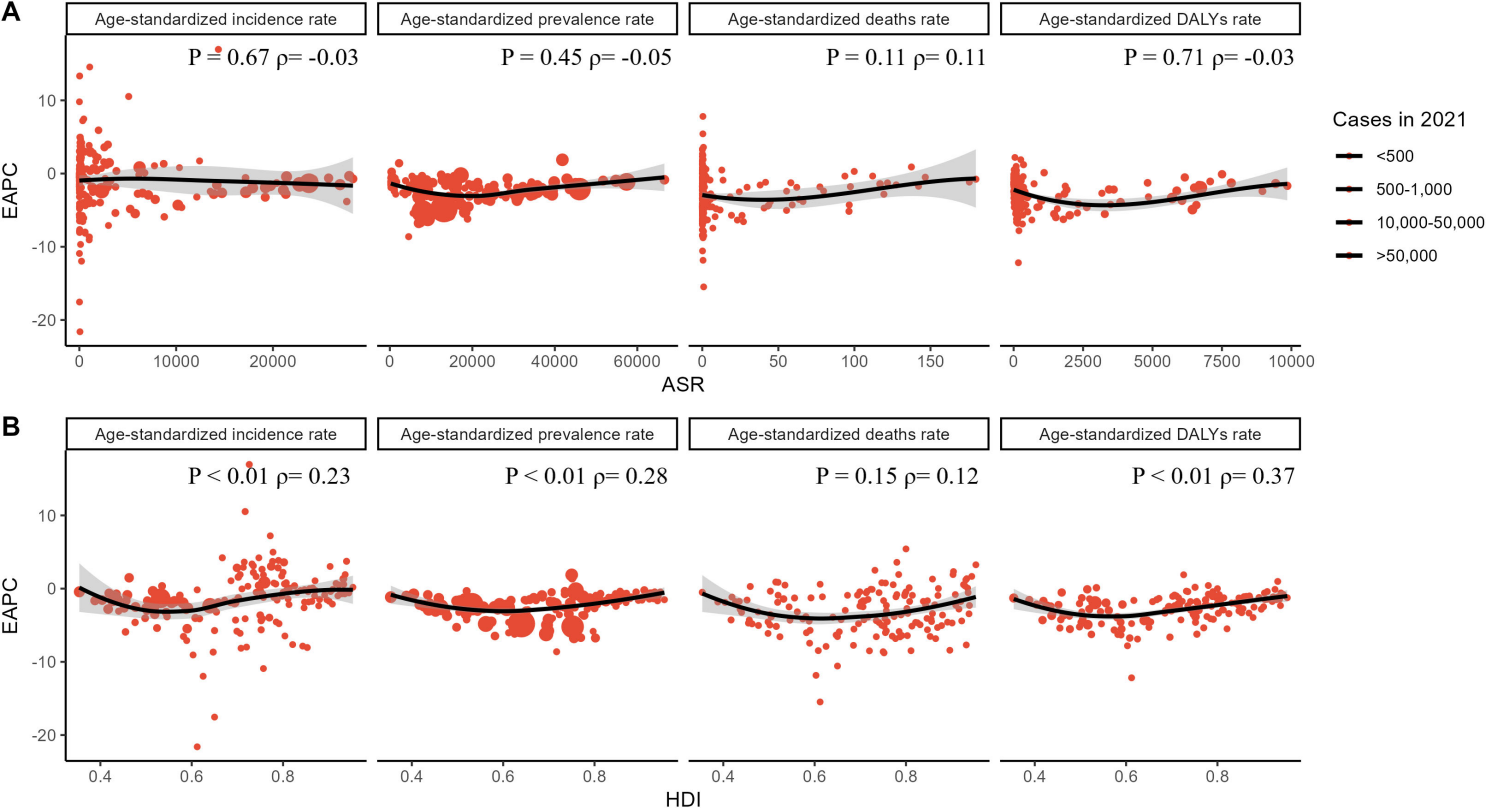
Association of EAPC with 2021 ASRs and HDIs for NTDs and Malaria.

The results demonstrate a discernible correlation between EAPCs and ASRs of NTDs and malaria, as well as HDIs in 2021, respectively (Fig. 4). Specifically, the ASRs recorded in 2021 for NTDs and malaria reflect the baseline disease burden, whereas the HDIs in 2021 serve as indicators of healthcare accessibility and a surrogate measure for the health system’s maturity within each country. A negative association was identified between EAPCs and ASRs across several health outcomes, including ASIR (P = 0.67, ρ = −0.03), ASPR (P = 0.45, ρ = −0.05), and ASDAR (P = 0.71, ρ = −0.03), though none of these associations were statistically significant. Conversely, for ASDR, a positive but non-significant correlation was observed (P = 0.11, ρ = 0.11).

In contrast, the EAPCs showed positive correlations with the HDI values. Notably, the correlations were statistically significant for ASIR (P < 0.01, ρ = 0.23), ASPR (P < 0.01, ρ = 0.28), and ASDAR (P < 0.01, ρ = 0.37) related to NTDs and malaria. This suggests that countries with higher HDI values tended to experience a greater increase in these disease burden indicators over time. However, the correlation between the EAPCs for deaths and HDI was not statistically significant (P = 0.15, ρ = 0.12), indicating that the relationship between HDI and mortality trends was less clear (Fig. 4).

### 3.4. The predicted results from 2022 to 2046

Projections based on the APC model consistently suggest that from 2022 to 2046, both males and females will experience a decline in the number of incidence cases, prevalence, and DALYs associated with these diseases. In contrast, the model forecasts an increase in the number of deaths during the same period. Regarding ASRs, the APC model predicts a slight decrease for both genders (Fig. S10, Table S5). Collectively, these findings highlight the persistence of a relatively smooth future disease burden, which is further corroborated by the results obtained from the ARIMA model and the ES model (Figs. S11–S12, Tables S6–S7).

### 3.5. Decomposition analysis of number of cases

Over the past 31 years, there has been a global increase in the number of prevalence cases, deaths, and DALYs associated with NTDs and malaria. However, the number of incidence cases did not followed the same upward trajectory. This suggests that while the ongoing disease burden remains significant, But the new cases is not growing very fast. The rise in incidence cases globally has been primarily driven by population growth, resulting in a significant increase (population: 78,517,444.45). Conversely, the decline in prevalence (aging: −108,327,184.8; population: −1,217,113,075) and DALYs (aging: −1,028,622.485; population: −20,147,001.37) has been predominantly attributed to positive aging and epidemiological shifts.

**Fig. 5:**
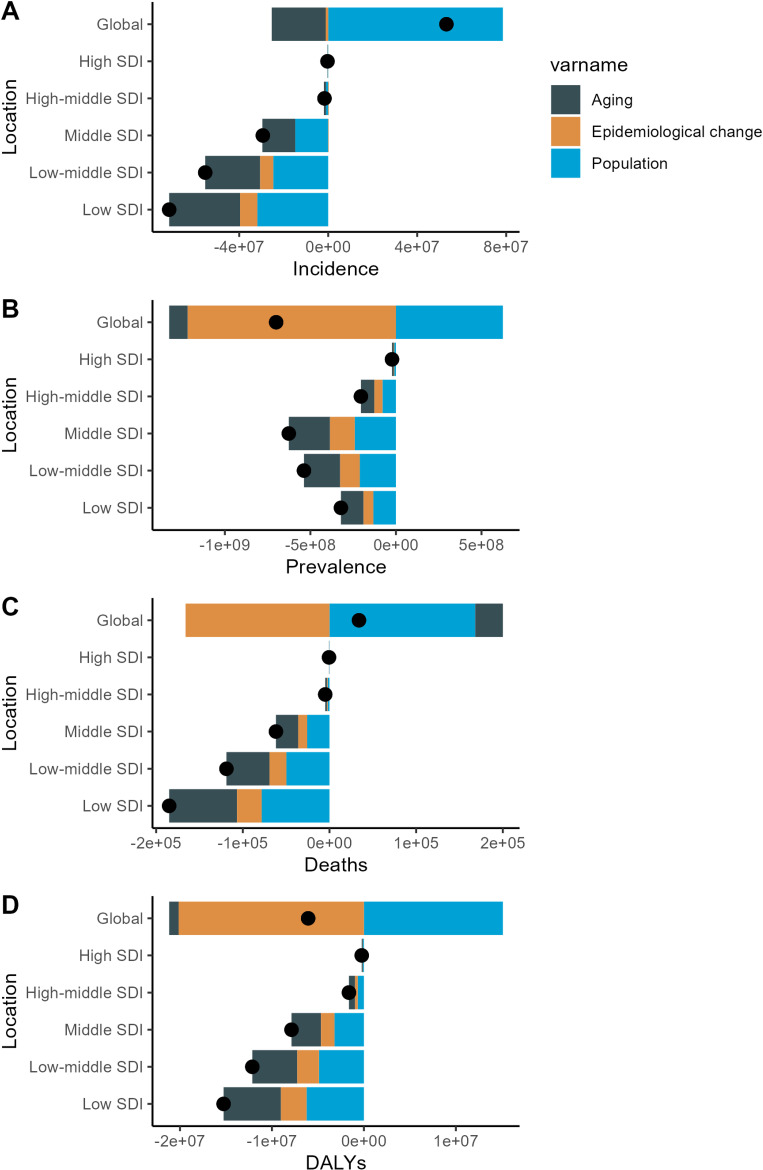
Drivers of Change in NTD and Malaria Case Numbers (1990–2021): Global and SDI Levels.

The reduction in the number of deaths has been primarily driven by significant epidemiological changes, which have led to a notable global decline in mortality associated with NTDs and malaria (epidemiological change: −166,060.893). When examining the trends across various SDI regions, the most pronounced declines in incidence, prevalence, deaths, and DALYs were observed in middle- to lower-SDI regions. These trends suggest that regions with lower levels of socioeconomic development have experienced the most substantial improvements in health outcomes over the study period (Fig. 5, Tables S8–S11).

### 3.6. Frontier analysis of ASRs

**Fig. 6:**
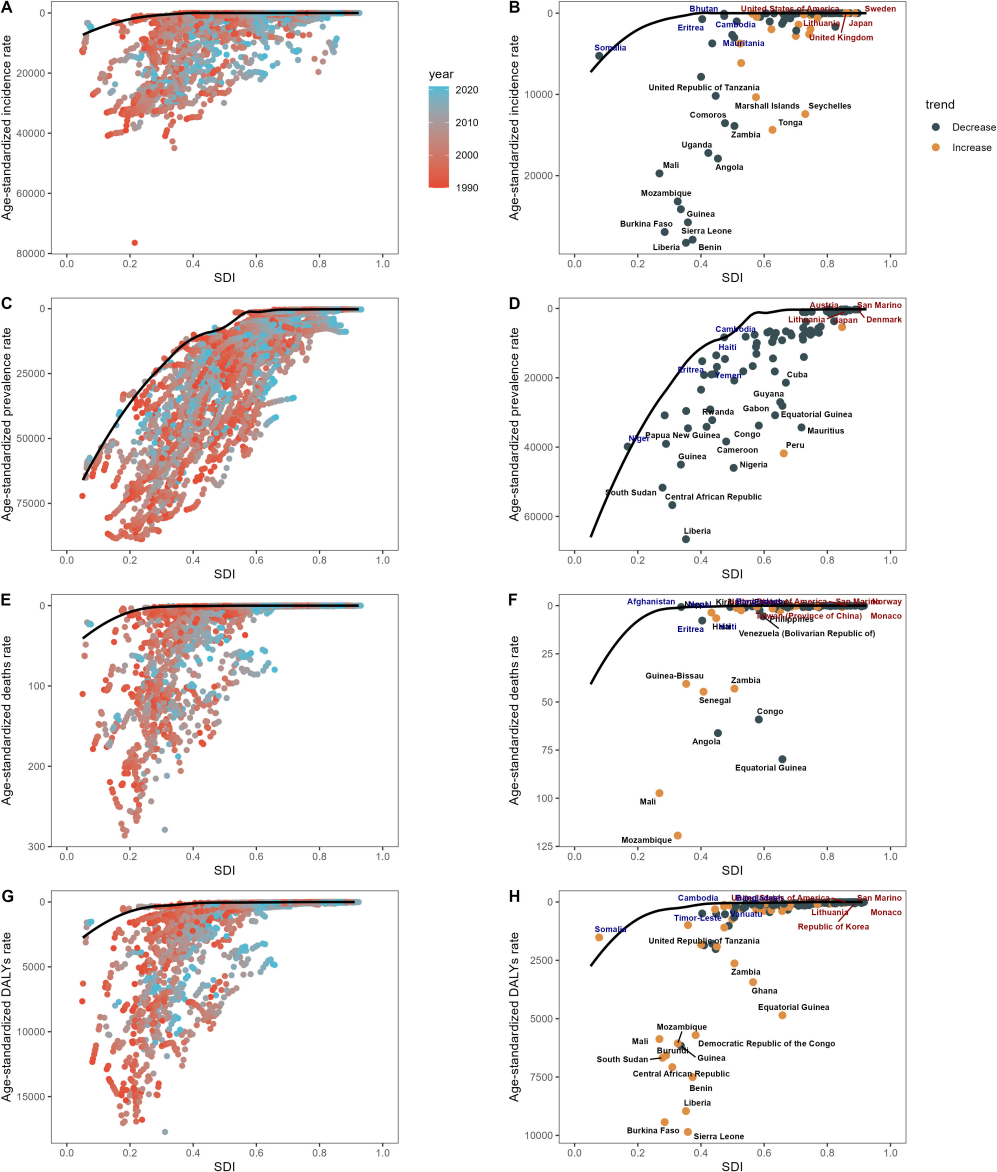
Frontier Analysis of NTD and Malaria Burden (ASR) versus Socio-demographic Index (SDI) in 2021.

Figure 6 presents an overview of the unrealized health gains that countries or regions with different developmental statuses could have achieved between 1990 and 2021. This visualization underscores the disparities in disease burden and the corresponding effective differences among nations with varying levels of sociodemographic development as observed in 2021. Notably, as the level of sociodemographic development increases, the effective difference tends to widen, suggesting that regions with higher SDI values possess a greater potential for further reducing their health burden. These findings, as depicted in Fig. 6, highlight the opportunities available for improvements in health outcomes, particularly in more developed regions.

## 4. Discussion

To the best of our knowledge, this is the most up-to-date study that provides a comprehensive assessment and quantification of the global disease burden of NTDs and malaria. In 2021, these diseases accounted for a significant portion of the global health burden [20], with notable disparities observed across various factors, including gender, age groups, SDI regions, GBD regions, and individual countries. This study highlights the uneven distribution of disease impact, emphasizing the need for targeted interventions and policies to address these disparities on a global scale.

Between 1990 and 2021, the majority of indicators, such as prevalence, deaths, and DALYs, demonstrated a downward trend both in the number of cases and their corresponding ASRs. However, incidence cases followed an upward trajectory during this period. The EAPCs were positively correlated with the ASRs related to NTDs and malaria, suggesting a relationship between these trends. Projections for the future indicate that this decline in disease burden is expected to continue through 2046.

Our decomposition analysis demonstrated that both demographic aging and epidemiological transitions have played significant roles in reducing the overall disease burden. In parallel, the frontier analysis indicated that countries or regions with higher SDI values possess considerable untapped potential to further lessen their health burdens.

The current study reveals complex trends in the burden of NTDs and malaria from 1990 to 2021. Despite an increasing incidence of cases, the ASIR showed a slight decline, which contrasts with the substantial reductions observed in prevalence, deaths, and DALYs. These findings align with previous research indicating improvements in disease control and management [30]. However, the persistence of high incidence rates, despite overall declines in other indicators, suggests ongoing challenges in preventing new cases, particularly in endemic regions [31]. The slight increase in deaths, albeit within wide uncertainty intervals, raises concerns about the effectiveness of current treatment and intervention strategies and necessitates further investigation [32]. Additionally, the decline in prevalence and DALYs rates, indicated by negative EAPC values, signifies progress but underscores the need for sustained efforts to achieve further reductions [33]. Comparable studies have emphasized the importance of integrated control programs and multi-stakeholder collaborations to effectively address the burden of NTDs and malaria [34, 35]. Therefore, continued surveillance, enhanced prevention measures, and scaled-up interventions are crucial to mitigate the impact of these diseases.

Our findings highlight gender-specific disparities in the burden of NTDs and malaria, with females exhibiting a higher incidence and ASIR while males carry a greater prevalence burden. This gender differentiation aligns with previous studies indicating that biological, socio-cultural, and environmental factors can influence disease susceptibility and outcomes [30]. The higher incidence among females may be attributed to physiological differences and hormonal changes, while the higher prevalence among males could be linked to occupational risks and behavioral patterns [36]. The trends observed in the number of cases and corresponding ASRs for both genders align with those of the overall population, indicating a declining disease burden with the exception of incidence cases. These findings are consistent with global efforts to control and manage NTDs and malaria through interventions such as mass drug administration, vector control, and enhancements in sanitation infrastructure [35, 37]. However, the persistence of high incidence rates suggests ongoing challenges in primary prevention, especially among females. The decreasing trends in prevalence, deaths, and DALYs underscore progress but emphasize the need for gender-tailored strategies to address remaining disparities [38]. Continued surveillance, gender-sensitive interventions, and community engagement are crucial to achieving equitable reductions in the burden of these diseases.

Our findings demonstrate a clear age-dependent pattern in the distribution of disease burden for NTDs and malaria in 2021, with children bearing a disproportionately higher burden. This aligns with numerous studies suggesting that children are particularly vulnerable to these diseases, primarily due to their underdeveloped immune systems and increased exposure to risk factors, including inadequate sanitation and limited access to healthcare services [30]. The persistence of this trend across middle age groups suggests a cumulative effect of early-life exposure on disease outcomes later in life. In contrast, the disease burden in older adults exhibits a different pattern, potentially reflecting age-related physiological changes and variations in immune responsiveness [39]. This age-specific vulnerability underscores the importance of targeted interventions that prioritize childhood health and address the underlying determinants of disease, such as poverty, inadequate housing, and lack of clean water and sanitation [35, 40]. Our findings contribute to a growing body of evidence that underscores the urgent need for comprehensive, integrated strategies tailored to different age groups in order to effectively reduce the global burden of NTDs and malaria. This study reinforces that public health interventions must be multifaceted—incorporating both broad-based approaches and age-specific measures—to address the unique vulnerabilities and health needs of various population segments. By aligning preventive, diagnostic, and treatment strategies with the demographic and epidemiological realities observed, policymakers and health practitioners can optimize resource allocation and intervention design. Ultimately, such tailored, integrated approaches are essential for achieving sustained improvements in global health outcomes and mitigating the impact of these diseases.

Our findings demonstrate a “J-shaped” relationship between the burden of NTDs and malaria and SDI regions, with the greatest burden concentrated in regions with low SDI values. This pattern aligns with findings from previous studies, which have consistently reported a strong association between poverty, inadequate sanitation, and a higher disease burden for NTDs and malaria [30, 40]. These studies emphasize the critical role that socioeconomic factors and environmental conditions play in shaping the prevalence and impact of these diseases, further highlighting the need for targeted interventions that address both health and underlying social determinants. While the ASIR remained relatively stable in low-middle and low SDI regions, it deviated from the trends observed in higher SDI regions between 1990 and 2021. This deviation suggests that improvements in healthcare access, as well as the implementation of disease control measures, may have been more pronounced in higher SDI regions. These advancements likely contributed to the differing trends in incidence, highlighting the influence of socioeconomic development and healthcare infrastructure on disease dynamics. However, the absolute number of deaths and DALYs cases declined across all SDI regions, indicating a general reduction in the overall impact of the disease. This trend suggests that, despite varying regional trajectories in incidence and prevalence, global health improvements—likely driven by better healthcare interventions, disease management strategies, and prevention efforts—have contributed to a decrease in the severe outcomes associated with NTDs and malaria. Notably, slight variations in incidence and prevalence cases were observed, potentially reflecting regional disparities in surveillance systems, reporting practices, and control strategies. These findings underscore the importance of implementing tailored intervention strategies that address the specific challenges encountered by different SDI regions, aiming to effectively mitigate the global burden of NTDs and malaria [35].

The findings of this study demonstrate considerable variations in the burden of NTDs and malaria across the 54 GBD regions. Africa, particularly Sub-Saharan Africa, emerged as a hotspot for incidence, deaths, and DALYs related to NTDs and malaria, aligning with previous research highlighting the region’s heightened disease burden [32, 41]. In contrast, High-income North America consistently ranked lowest in incidence cases, indicating a stark disparity in disease prevalence across economic strata. Notably, Australasia and Commonwealth High Income regions, despite high absolute counts of prevalence, deaths, and DALYs, exhibited the lowest ASRs for mortality and morbidity. This counterintuitive finding can be primarily attributed to two key factors supported by GBD-related research. Firstly, GBD 2021 Collaborators reported that these regions typically feature aging populations [20]. Since the elderly are more susceptible to diseases, this naturally inflates the absolute disease burden metrics. However, ASRs adjust for age-related risk differences, normalizing the data and thus resulting in lower rates compared to regions with younger demographics. Secondly, these high-income areas possess well-developed healthcare systems. Advanced diagnostic capabilities, abundant medical resources, and skilled healthcare professionals enable early disease detection and effective management, reducing disease progression and mortality. Such interventions directly contribute to the observed low ASRs, despite the high absolute burden, suggesting the efficacy of healthcare strategies in these regions.

Hierarchical clustering analysis revealed distinct patterns of variation in ASRs across regions. Significant increases in ASRs were observed in Western Europe, Eastern Europe, and several high-income regions, indicating a rise in disease burden in these areas. In contrast, notable decreases were seen in Southeast Asia, East Asia, and several lower-middle-income regions, suggesting improvements in health outcomes and the effectiveness of disease control measures in these regions. These findings highlight the urgent need for targeted interventions in high-burden areas, particularly in regions where disease rates remain elevated. At the same time, they emphasize the importance of identifying and adapting models of effective disease management from regions with lower ASRs, which may offer valuable insights and strategies for reducing the burden of NTDs and malaria in other parts of the world [42, 43]. This approach could facilitate more efficient and context-specific public health responses across diverse regions.

The global burden of NTDs and malaria exhibits considerable variability across countries and territories. Our findings reveal that Nigeria and India bear the heaviest burdens in terms of incidence, deaths, and DALYs, respectively, aligning with previous studies [41, 42]. Several factors contribute to this. Large populations in both countries increase the absolute number of disease cases, as more people mean greater exposure to pathogens. Socioeconomic challenges also play a key role. Poverty in Nigeria limits access to clean water, sanitation, and housing, facilitating disease transmission. In India, despite economic growth, many lack basic health services and live in poor sanitary conditions, increasing disease vulnerability. Weak public health infrastructure further exacerbates the burden. Nigeria’s health system struggles with limited resources and insufficient funding, hindering disease control. In India, uneven distribution of healthcare resources, especially in rural areas, leads to delayed treatment. Climate and environment are also relevant. Nigeria’s tropical climate favors the breeding of disease vectors like mosquitoes. India’s diverse geography, along with poor drainage and certain agricultural practices, supports the spread of pathogens. Conversely, San Marino and Tokelau reported the lowest incidence and prevalence, and deaths and DALYs, respectively, suggesting that these areas may have more effective control measures or lower exposure risks. Regarding ASRs, Liberia, Burkina Faso, and Sierra Leone exhibited the highest incidence, death, and DALY rates, respectively, while Ireland, Norway, Finland, and Canada reported the lowest across these categories. These disparities highlight the need for tailored intervention strategies tailored to specific regions. Furthermore, our analysis of trends from 1990 to 2021 reveals notable variations in the EAPC of ASRs. Eswatini, Maldives, Ecuador, and Vanuatu exhibited the most significant reductions in incidence, prevalence, death, and DALY rates, respectively, reflecting the impact of effective public health interventions in these countries. In contrast, Monaco and Peru reported increases in incidence, death, and prevalence rates, respectively, which require urgent attention. These findings underscore the dynamic nature of NTD and malaria burdens and the importance of continuous monitoring and evaluation of intervention strategies [35, 43].

Our study highlights intriguing correlations between the EAPC and ASRs of NTDs and malaria, alongside HDI scores in 2021. The ASRs for these diseases in 2021 represent a baseline measure of disease burden, whereas HDIs reflect healthcare accessibility and health system maturity. While we found non-significant negative associations between EAPCs and ASRs for incidence, prevalence, and DALYs, a positive, albeit non-significant trend was noted for ASDR. This contrasts with prior studies reporting negative correlations between disease burden and health system development [30, 44]. Notably, significant positive correlations were observed between the EAPCs for incidence, prevalence, and DALYs of NTDs and malaria and the HDI, suggesting that advancements in human development may be linked to favorable disease trends over time. However, the lack of significance for deaths might indicate that reducing mortality requires more targeted interventions beyond general health system improvements [35]. These findings underscore the complex interplay between disease dynamics and socioeconomic factors, necessitating multifaceted approaches to disease control and elimination.

Our projected outcomes from 2022 to 2046, using the APC model, suggest a decreasing trend in incidence, prevalence, and DALYs for both genders, with a contrasting increase in deaths. This pattern aligns with previous studies that report declining disease incidence and prevalence due to advancements in healthcare and preventive measures [45, 46]. However, the observed increase in deaths necessitates further investigation, possibly indicating a shift towards more severe disease manifestations or ineffective treatment strategies [47]. The slight decrease in ASRs for both genders supports the overall trend of a decreasing disease burden. These findings are consistent with the ARIMA and ES model projections, reinforcing the robustness of our predictions. Collectively, these models offer a comprehensive perspective on the future disease landscape, emphasizing the necessity of targeted interventions to address the rising mortality trend while sustaining the reductions in incidence, prevalence, and DALYs.

Our decomposition analysis highlights divergent trends in the number of cases for NTDs and malaria over the past 31 years. While incidence have increased globally, primarily due to population growth [48], prevalence and DALYs cases have decreased, primarily driven by aging and epidemiological changes [49, 50]. Notably, the reduction in deaths is solely attributed to epidemiological changes [51]. These results align with earlier research, which suggests that advancements in healthcare, sanitation, and treatment accessibility have played a crucial role in reducing the burden of these diseases [52]. The decreases in incidence, prevalence, mortality, and DALYs in middle- to lower-SDI regions highlight the success of the targeted interventions carried out in these areas [53]. However, the rising incidence driven by population growth underscore the necessity for sustained and intensified efforts to control and prevent these diseases on a global scale.

Our frontier analysis of ASRs offers an in-depth view of untapped health improvements across countries and regions with varying levels of sociodemographic development from 1990 to 2021. Our findings align with previous studies indicating that as sociodemographic indices improve, the effective difference in health outcomes, particularly in disease burden, tends to widen [53, 54]. This trend suggests that while higher SDI regions may have achieved significant health improvements, they also possess greater potential for further gains due to advanced healthcare systems and resources [55]. However, the increasing effective difference also highlights the disparities in health outcomes between developed and less developed regions, emphasizing the need for targeted interventions to bridge these gaps [56]. Our findings highlight the need for ongoing monitoring and assessment of health policies and programs to guarantee equitable health outcomes worldwide.

Nevertheless, it is important to acknowledge the inherent limitations of this study. First, the disease burden evaluation was primarily carried out at the national and regional levels. It is worth noting that some countries, due to their vast geographical extents, exhibit potentially significant variations in disease burden across different provinces within the same country. This limitation hindered our ability to comprehensively capture the spatial heterogeneity of the disease burden. Secondly, the GBD database presented certain deficiencies. Notably, ensuring data quality assurance posed a significant challenge, which may have implications for the accuracy and reliability of our findings. This underscores the necessity for further refinements in data collection and validation processes to enhance the overall quality of the GBD database. Thirdly, another key limitation of this study was the inability to incorporate environmental factors such as temperature, relative humidity, dusty environments, and sanitation conditions into the analysis due to the lack of long-term environmental monitoring data and inconsistent statistical calibers of sanitation facility coverage rates in the study areas. Finally, this study was constrained by the lack of general health status and comorbidity data (such as diabetes, cardiovascular diseases) in the GBD framework, which precluded analysis. Future studies incorporating electronic health records or cohort data could bridge this gap.

## 5. Conclusion

This study offers the latest analysis of the global, regional, and national burden of NTDs and malaria, focusing on incidence, prevalence, mortality, and DALYs. The findings emphasize the urgent need for targeted interventions to effectively decrease the global burden of NTDs and malaria. Future research should concentrate on identifying the underlying drivers and creating effective strategies to tackle them, especially in high-risk regions and vulnerable populations.

## Abbreviations

NTDs: Neglected tropical diseases
GBD: Global Burden of Disease
EAPCs: Estimated annual percentage change
ASRs: Age-standardized rates
HDI: Human Development Index
SDI: Socio-demographic Index
DALYs: Disability-adjusted life-years
ASIR: Age-standardized incidence rate
ASPR: Age-standardized prevalence rate
ASDR: Age-standardized deaths rate
ASDAR: Age-standardized DALYs rate
APC: age-period-cohort
ARIMA: Autoregressive Integrated Moving Average
ES: Exponential smoothing
UI: Uncertainty interval
CI: Confidence interval

## Supplementary information

**Additional file 1:** **Figure S1.** Numbers and age-standardized rates of neglected tropical diseases and malaria-related incidence, prevalence, deaths, and DALYs for both sexes in 2021. Abbreviations: DALYs, disability-adjusted life years. **Figure S2.** Numbers and age-standardized rates of neglected tropical diseases and malaria-related incidence, prevalence, deaths, and DALYs for different age groups in 2021. Abbreviations: DALYs, disability-adjusted life years. **Figure S3.** Numbers and age-standardized rates of neglected tropical diseases and malaria-related incidence, prevalence, deaths, and DALYs for different SDI regions in 2021. Abbreviations: DALYs, disability-adjusted life years. **Figure S4.** Age-standardized rates of incidence, prevalence, deaths, and DALYs of neglected tropical diseases and malaria across countries and territories by socio-demographic index for both sexes, 1990–2021. The black line was an adaptive association fitted with adaptive Loess regression based on all data points. Abbreviations: DALYs, disability-adjusted-life-years. **Figure S5.** Numbers and age-standardized rates of neglected tropical diseases and malaria-related incidence, prevalence, deaths, and DALYs for different GBD regions in 2021. Abbreviations: DALYs, disability-adjusted life years. **Figure S6.** Numbers and age-standardized rates of neglected tropical diseases and malaria-related incidence, prevalence, deaths, and DALYs across countries and territories in 2021. Abbreviations: DALYs, disability-adjusted life years. **Figure S7.** Trends in the numbers and age-standardized rates of neglected tropical diseases and malaria-related incidence, prevalence, deaths, and DALYs globally by sexes from 1990 to 2021. Abbreviations: DALYs, disability-adjusted-life-years. **Figure S8.** Trends in the numbers and age-standardized rates of neglected tropical diseases and malaria-related incidence, prevalence, deaths, and DALYs globally by age groups from 1990 to 2021. Abbreviations: DALYs, disability-adjusted-life-years. **Figure S9.** Trends in the numbers and age-standardized rates of neglected tropical diseases and malaria-related incidence, prevalence, deaths, and DALYs globally by SDI regions from 1990 to 2021. Abbreviations: DALYs, disability-adjusted-life-years. **Figure S10.** The predicted results in the neglected tropical diseases and malaria-related numbers and age-standardized rates of incidence, prevalence, deaths, and DALYs by sex globally from 2022 to 2046 of the APC model. Abbreviations: DALYs, disability-adjusted-life-year; APC, age-period-cohort. **Figure S11.** The predicted results in the neglected tropical diseases and malaria-related numbers and age-standardized rates of incidence, prevalence, deaths, and DALYs by sex globally from 2022 to 2046 of the ARIMA model. Abbreviations: DALYs, disability-adjusted-life-year; ARIMA, Autoregressive Integrated Moving Average. **Figure S12.** The predicted results in the neglected tropical diseases and malaria-related numbers and age-standardized rates of incidence, prevalence, deaths, and DALYs by sex globally from 2022 to 2046 of the ES model. Abbreviations: DALYs, disability-adjusted-life-year; ES, Exponential Smoothing. **Table S1.** The number of incidence cases and the age-standardized incidence rate of neglected tropical diseases and malaria in 1990 and 2021, and its trends from 1990 to 2021 globally. **Table S2.** The number of prevalence cases and the age-standardized prevalence rate of neglected tropical diseases and malaria in 1990 and 2021, and its trends from 1990 to 2021 globally. **Table S3.** The number of deaths cases and the age-standardized deaths rate of neglected tropical diseases and malaria in 1990 and 2021, and its trends from 1990 to 2021 globally. **Table S4.** The number of DALYs cases and the age-standardized DALYs rate of neglected tropical diseases and malaria in 1990 and 2021, and its trends from 1990 to 2021 globally. Abbreviations: DALYs, disability-adjusted-life-years. **Table S5.** The predicted results in the neglected tropical diseases and malaria-related numbers and age-standardized rates of incidence, prevalence, deaths, DALYs by sex globally from 2022 to 2046 of the APC model. Abbreviations: DALYs, disability-adjusted-life-years; APC, age-period-cohort. **Table S6.** The predicted results in the neglected tropical diseases and malaria-related numbers and age-standardized rates of incidence, prevalence, deaths, DALYs by sex globally from 2022 to 2046 of the ARIMA model. Abbreviations: DALYs, disability-adjusted-life-years; ARIMA, Autoregressive Integrated Moving Average. **Table S7.** The predicted results in the neglected tropical diseases and malaria-related numbers and age-standardized rates of incidence, prevalence, deaths, DALYs by sex globally from 2022 to 2046 of the ES model. Abbreviations: DALYs, disability-adjusted-life-years; ES, exponential smoothing. **Table S8.** Changes in neglected tropical diseases and malaria-related incidence number according to population-level determinants and causes from 1990 to 2021. **Table S9.** Changes in neglected tropical diseases and malaria-related prevalence number according to population-level determinants and causes from 1990 to 2021. **Table S10.** Changes in neglected tropical diseases and malaria-related deaths number according to population-level determinants and causes from 1990 to 2021. **Table S11.** Changes in neglected tropical diseases and malaria-related DALYs number according to population-level determinants and causes from 1990 to 2021.

## Declarations

### Availability of data and material

The data used in this study are available from the GBD 2021 database (https://ghdx.healthdata.org/gbd-2021).
