# Supplementary material for: Global, regional, and national burden of neglected tropical diseases and malaria, 1990–2021
Source: Environ Health Prev Med. 2025 Jul 17;30:54. doi: 10.1265/ehpm.25-00038 (PMC12301076; doi:10.1265/ehpm.25-00038)
Supplement: Supplementary file 1 — Additional file 1: Figure S1. Numbers and age-standardized rates of neglected tropical diseases and malaria-related incidence, prevalence, deaths, and DALYs for both sexes in 2021. Abbreviations: DALYs, disability-adjusted life years. Figure S2. Numbers and age-standardized rates of neglected tropical diseases and malaria-related incidence, prevalence, deaths, and DALYs for different age groups in 2021. Abbreviations: DALYs, disability-adjusted life years. Figure S3. Numbers and age-standardized rates of neglected tropical diseases and malaria-related incidence, prevalence, deaths, and DALYs for different SDI regions in 2021. Abbreviations: DALYs, disability-adjusted life years. Figure S4. Age-standardized rates of incidence, prevalence, deaths, and DALYs of neglected tropical diseases and malaria across countries and territories by socio-demographic index for both sexes, 1990–2021. The black line was an adaptive association fitted with adaptive Loess regression based on all data points. Abbreviations: DALYs, disability-adjusted-life-years. Figure S5. Numbers and age-standardized rates of neglected tropical diseases and malaria-related incidence, prevalence, deaths, and DALYs for different GBD regions in 2021. Abbreviations: DALYs, disability-adjusted life years. Figure S6. Numbers and age-standardized rates of neglected tropical diseases and malaria-related incidence, prevalence, deaths, and DALYs across countries and territories in 2021. Abbreviations: DALYs, disability-adjusted life years. Figure S7. Trends in the numbers and age-standardized rates of neglected tropical diseases and malaria-related incidence, prevalence, deaths, and DALYs globally by sexes from 1990 to 2021. Abbreviations: DALYs, disability-adjusted-life-years. Figure S8. Trends in the numbers and age-standardized rates of neglected tropical diseases and malaria-related incidence, prevalence, deaths, and DALYs globally by age groups from 1990 to 2021. Abbreviations: DALYs, disability-adjusted-lif [file ehpm-30-054-s001.docx]

**Global, regional, and national burden of neglected tropical diseases and malaria, 1990-2021**

**Figure legend**

**Figure S1.** Numbers and age-standardized rates of neglected tropical diseases and malaria-related incidence, prevalence, deaths, and DALYs for both sexes in 2021. Abbreviations: DALYs, disability-adjusted life years.

**Figure S2.** Numbers and age-standardized rates of neglected tropical diseases and malaria-related incidence, prevalence, deaths, and DALYs for different age groups in 2021. Abbreviations: DALYs, disability-adjusted life years.

**Figure S3.** Numbers and age-standardized rates of neglected tropical diseases and malaria-related incidence, prevalence, deaths, and DALYs for different SDI regions in 2021. Abbreviations: DALYs, disability-adjusted life years.

**Figure S4.** Age-standardized rates of incidence, prevalence, deaths, and DALYs of neglected tropical diseases and malaria across countries and territories by socio-demographic index for both sexes, 1990-2021. The black line was an adaptive association fitted with adaptive Loess regression based on all data points. Abbreviations: DALYs, disability-adjusted-life-years.

**Figure S5.** Numbers and age-standardized rates of neglected tropical diseases and malaria-related incidence, prevalence, deaths, and DALYs for different GBD regions in 2021. Abbreviations: DALYs, disability-adjusted life years.

**Figure S6.** Numbers and age-standardized rates of neglected tropical diseases and malaria-related incidence, prevalence, deaths, and DALYs across countries and territories in 2021. Abbreviations: DALYs, disability-adjusted life years.

**Figure S7.** Trends in the numbers and age-standardized rates of neglected tropical diseases and malaria-related incidence, prevalence, deaths, and DALYs globally by sexes from 1990 to 2021. Abbreviations: DALYs, disability-adjusted-life-years.

**Figure S8.** Trends in the numbers and age-standardized rates of neglected tropical diseases and malaria-related incidence, prevalence, deaths, and DALYs globally by age groups from 1990 to 2021. Abbreviations: DALYs, disability-adjusted-life-years.

**Figure S9.** Trends in the numbers and age-standardized rates of neglected tropical diseases and malaria-related incidence, prevalence, deaths, and DALYs globally by SDI regions from 1990 to 2021. Abbreviations: DALYs, disability-adjusted-life-years.

**Figure S10.** The predicted results in the neglected tropical diseases and malaria-related numbers and age-standardized rates of incidence, prevalence, deaths, and DALYs by sex globally from 2022 to 2046 of the APC model. Abbreviations: DALYs, disability-adjusted-life-year; APC, age-period-cohort.

**Figure S11.** The predicted results in the neglected tropical diseases and malaria-related numbers and age-standardized rates of incidence, prevalence, deaths, and DALYs by sex globally from 2022 to 2046 of the ARIMA model. Abbreviations: DALYs, disability-adjusted-life-year; ARIMA, Autoregressive Integrated Moving Average.

**Figure S12.** The predicted results in the neglected tropical diseases and malaria-related numbers and age-standardized rates of incidence, prevalence, deaths, and DALYs by sex globally from 2022 to 2046 of the ES model. Abbreviations: DALYs, disability-adjusted-life-year; ES, Exponential Smoothing.

**Table legend**

**Table S1.** The number of incidence cases and the age-standardized incidence rate of neglected tropical diseases and malaria in 1990 and 2021, and its trends from 1990 to 2021 globally.

**Table S2.** The number of prevalence cases and the age-standardized prevalence rate of neglected tropical diseases and malaria in 1990 and 2021, and its trends from 1990 to 2021 globally.

**Table S3.** The number of deaths cases and the age-standardized deaths rate of neglected tropical diseases and malaria in 1990 and 2021, and its trends from 1990 to 2021 globally.

**Table S4.** The number of DALYs cases and the age-standardized DALYs rate of neglected tropical diseases and malaria in 1990 and 2021, and its trends from 1990 to 2021 globally. Abbreviations: DALYs, disability-adjusted-life-years.

**Table S5.** The predicted results in the neglected tropical diseases and malaria-related numbers and age-standardized rates of incidence, prevalence, deaths, DALYs by sex globally from 2022 to 2046 of the APC model. Abbreviations: DALYs, disability-adjusted-life-years; APC, age-period-cohort.

**Table S6.** The predicted results in the neglected tropical diseases and malaria-related numbers and age-standardized rates of incidence, prevalence, deaths, DALYs by sex globally from 2022 to 2046 of the ARIMA model. Abbreviations: DALYs, disability-adjusted-life-years; ARIMA, Autoregressive Integrated Moving Average.

**Table S7.** The predicted results in the neglected tropical diseases and malaria-related numbers and age-standardized rates of incidence, prevalence, deaths, DALYs by sex globally from 2022 to 2046 of the ES model. Abbreviations: DALYs, disability-adjusted-life-years; ES, exponential smoothing.

**Table S8.** Changes in neglected tropical diseases and malaria-related incidence number according to population-level determinants and causes from 1990 to 2021.

**Table S9.** Changes in neglected tropical diseases and malaria-related prevalence number according to population-level determinants and causes from 1990 to 2021.

**Table S10.** Changes in neglected tropical diseases and malaria-related deaths number according to population-level determinants and causes from 1990 to 2021.

**Table S11.** Changes in neglected tropical diseases and malaria-related DALYs number according to population-level determinants and causes from 1990 to 2021.

**Figure S1.** Numbers and age-standardized rates of neglected tropical diseases and malaria-related incidence, prevalence, deaths, and DALYs for both sexes in 2021. Abbreviations: DALYs, disability-adjusted life years.


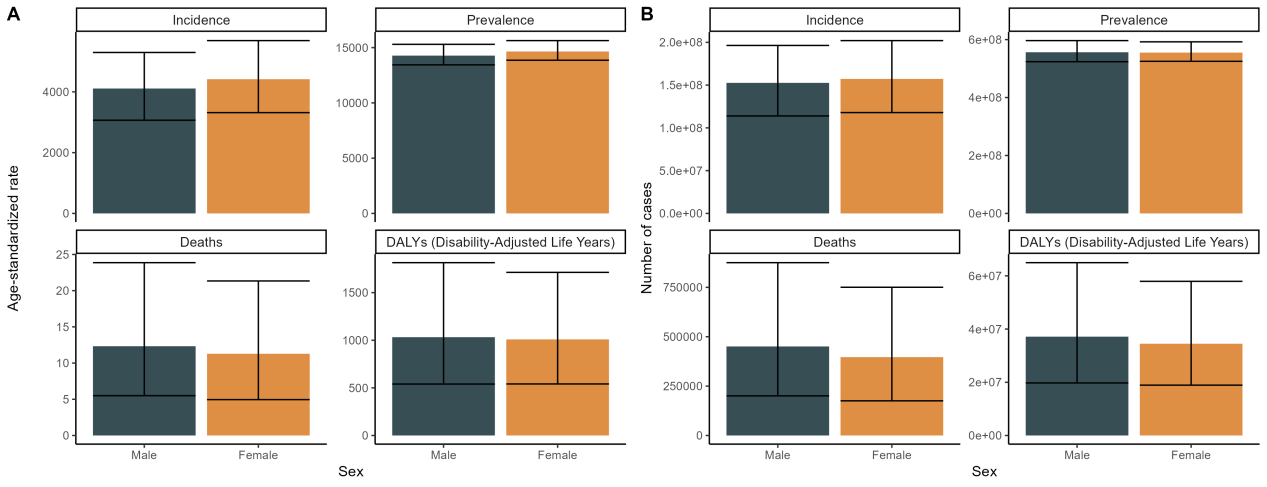


**Figure S2.** Numbers and age-standardized rates of neglected tropical diseases and malaria-related incidence, prevalence, deaths, and DALYs for different age groups in 2021. Abbreviations: DALYs, disability-adjusted life years.

**
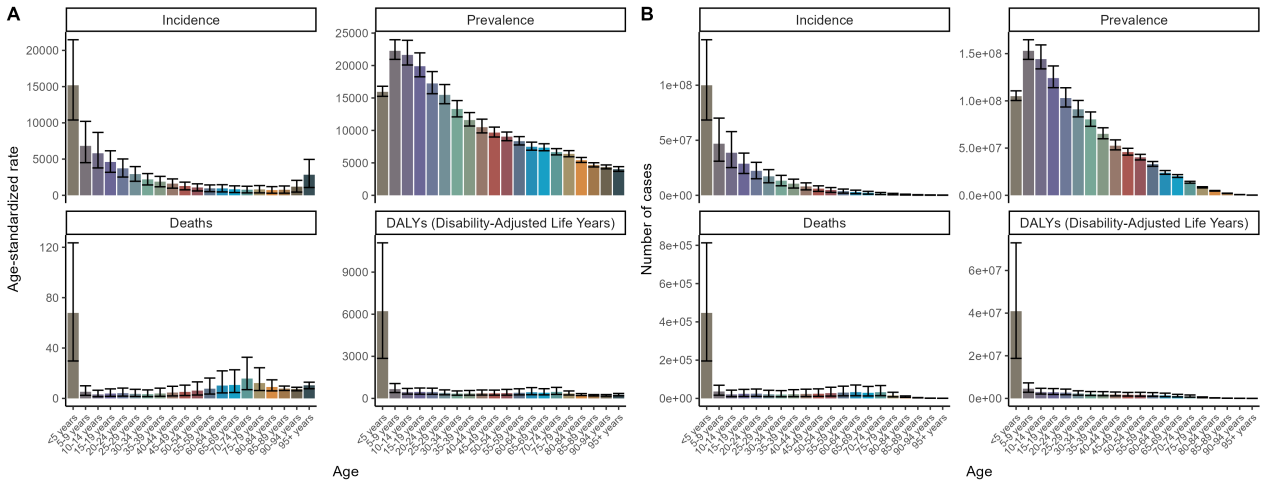
**

**Figure S3.** Numbers and age-standardized rates of neglected tropical diseases and malaria-related incidence, prevalence, deaths, and DALYs for different SDI regions in 2021. Abbreviations: DALYs, disability-adjusted life years.


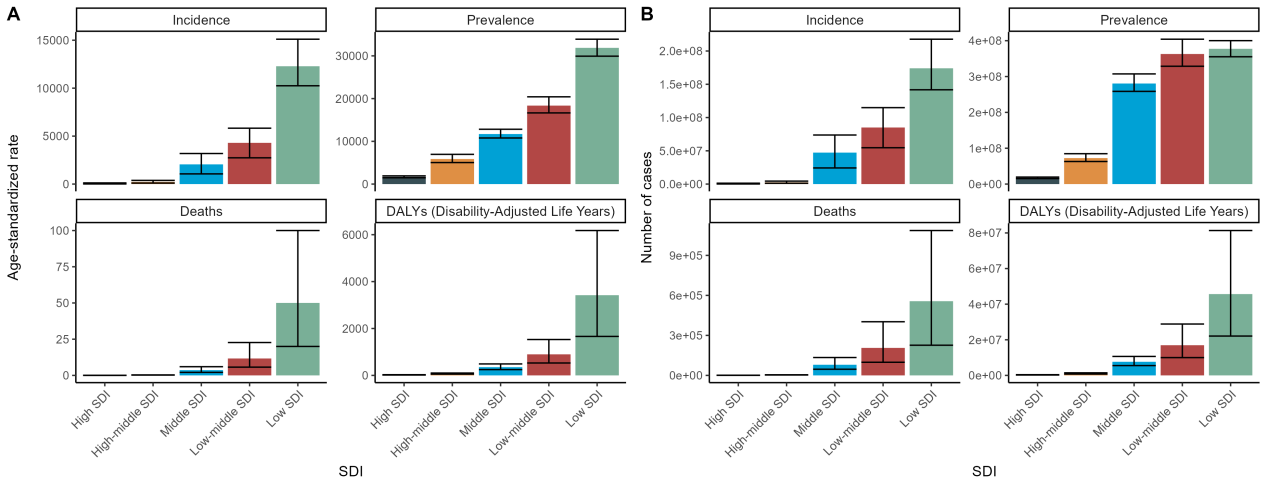


**Figure S4.** Age-standardized rates of incidence, prevalence, deaths, and DALYs of neglected tropical diseases and malaria across countries and territories by socio-demographic index for both sexes, 1990-2021. The black line was an adaptive association fitted with adaptive Loess regression based on all data points. Abbreviations: DALYs, disability-adjusted-life-years.


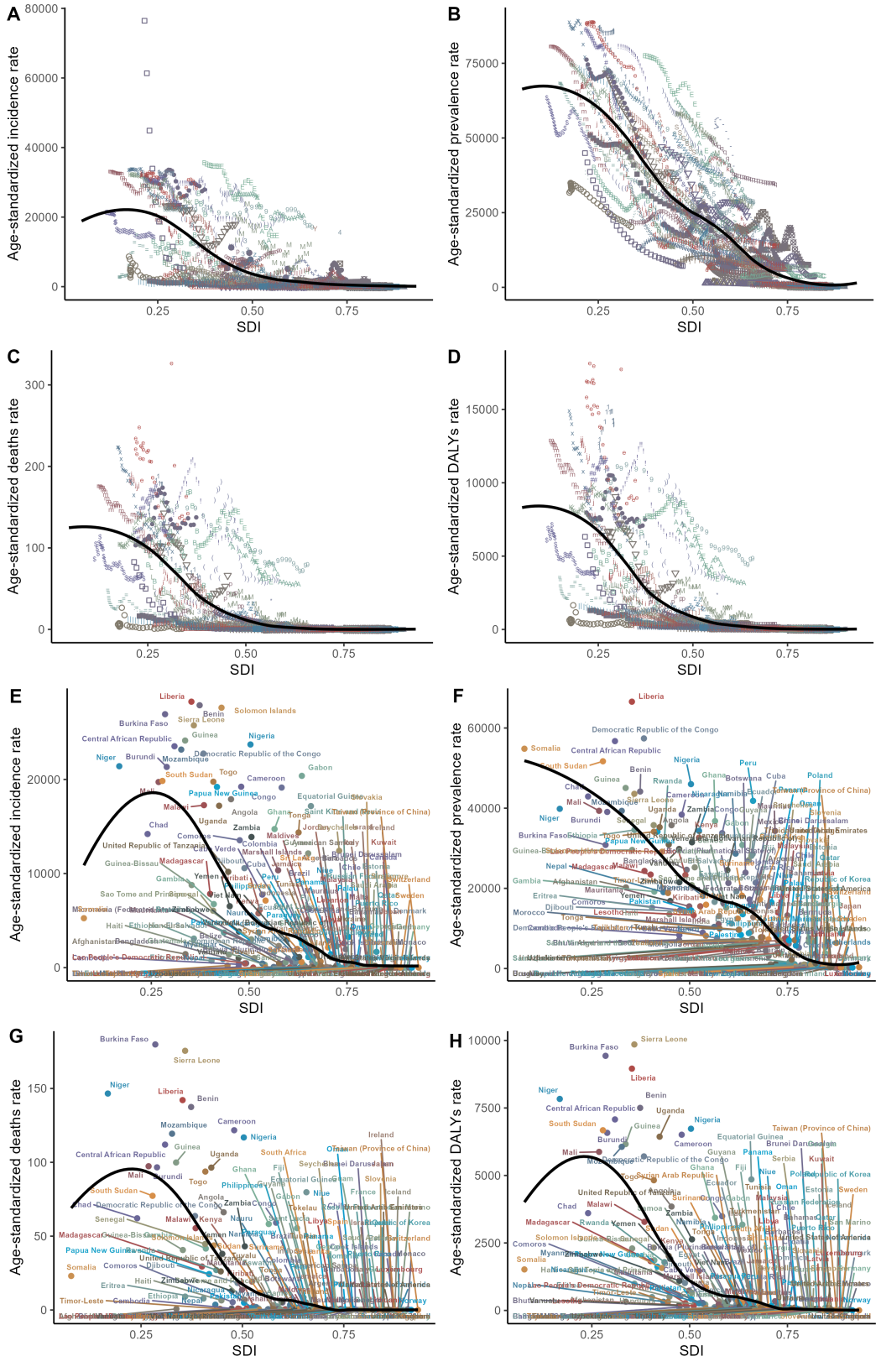


**Figure S5.** Numbers and age-standardized rates of neglected tropical diseases and malaria-related incidence, prevalence, deaths, and DALYs for different GBD regions in 2021. Abbreviations: DALYs, disability-adjusted life years.


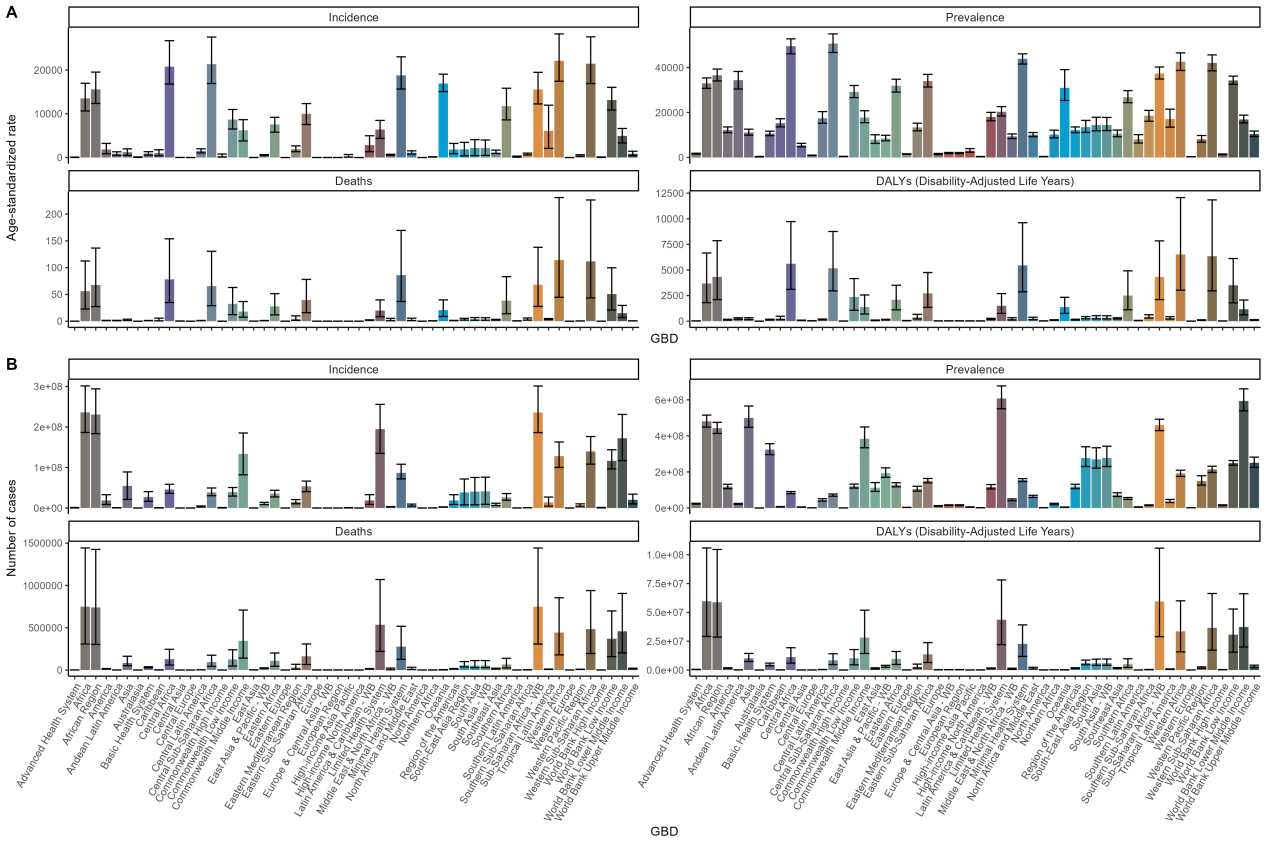


**Figure S6.** Numbers and age-standardized rates of neglected tropical diseases and malaria-related incidence, prevalence, deaths, and DALYs across countries and territories in 2021. Abbreviations: DALYs, disability-adjusted life years.


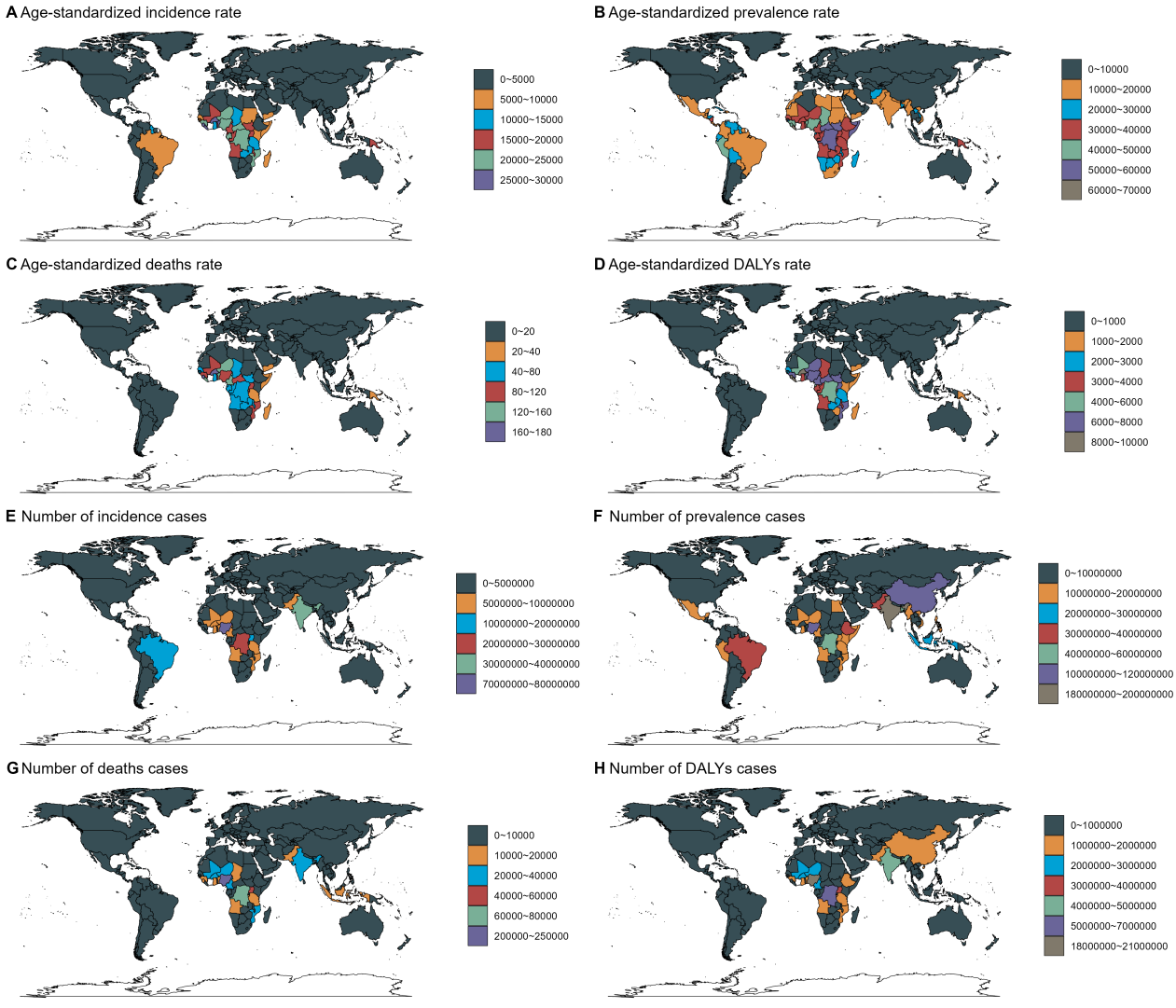


**Figure S7.** Trends in the numbers and age-standardized rates of neglected tropical diseases and malaria-related incidence, prevalence, deaths, and DALYs globally by sexes from 1990 to 2021. Abbreviations: DALYs, disability-adjusted-life-years.


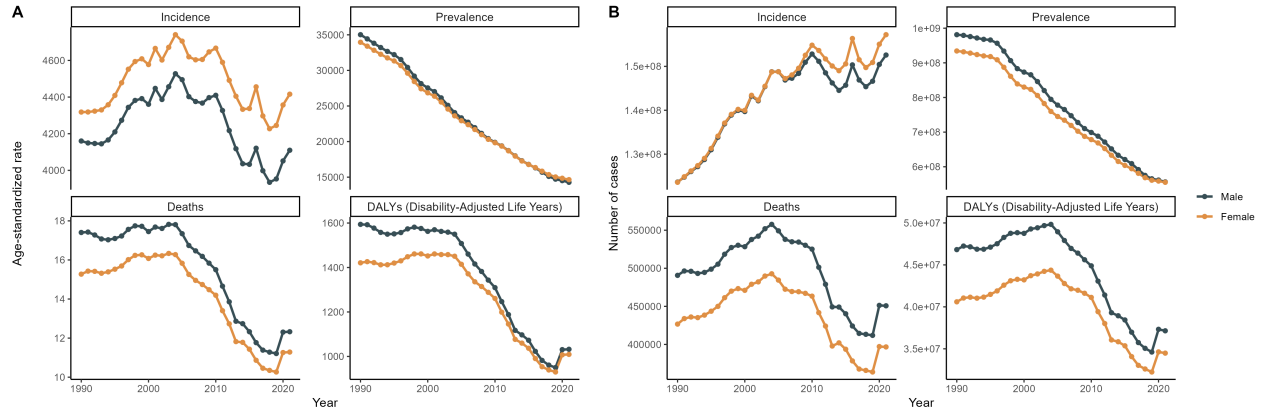


**Figure S8.** Trends in the numbers and age-standardized rates of neglected tropical diseases and malaria-related incidence, prevalence, deaths, and DALYs globally by age groups from 1990 to 2021. Abbreviations: DALYs, disability-adjusted-life-years.


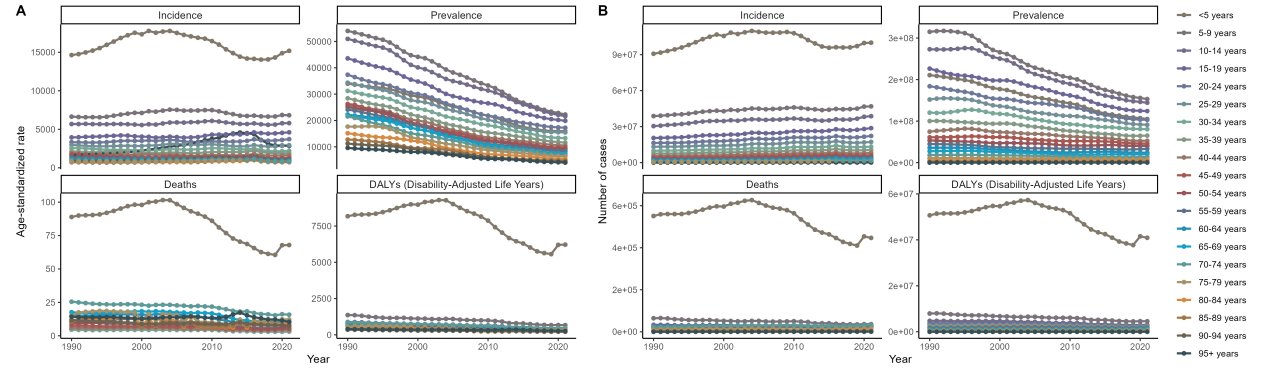


**Figure S9.** Trends in the numbers and age-standardized rates of neglected tropical diseases and malaria-related incidence, prevalence, deaths, and DALYs globally by SDI regions from 1990 to 2021. Abbreviations: DALYs, disability-adjusted-life-years.


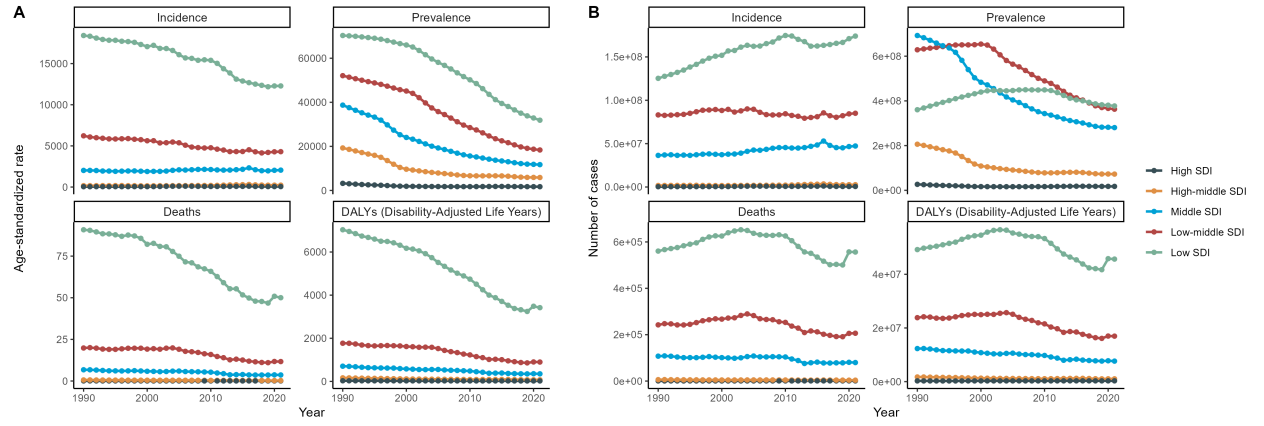


**Figure S10.** The predicted results in the neglected tropical diseases and malaria-related numbers and age-standardized rates of incidence, prevalence, deaths, and DALYs by sex globally from 2022 to 2046 of the APC model. Abbreviations: DALYs, disability-adjusted-life-year; APC, age-period-cohort.

**
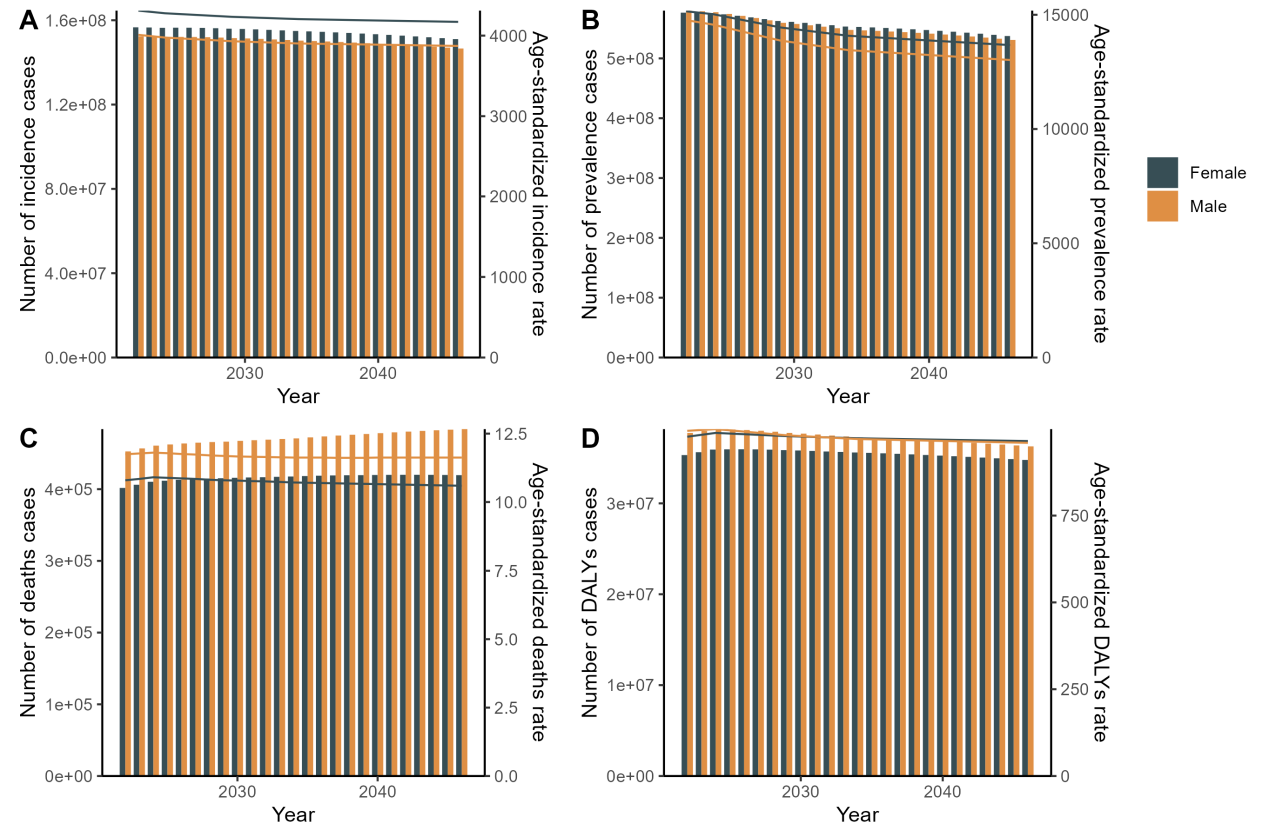
**

**Figure S11.** The predicted results in the neglected tropical diseases and malaria-related numbers and age-standardized rates of incidence, prevalence, deaths, and DALYs by sex globally from 2022 to 2046 of the ARIMA model. Abbreviations: DALYs, disability-adjusted-life-year; ARIMA, Autoregressive Integrated Moving Average.


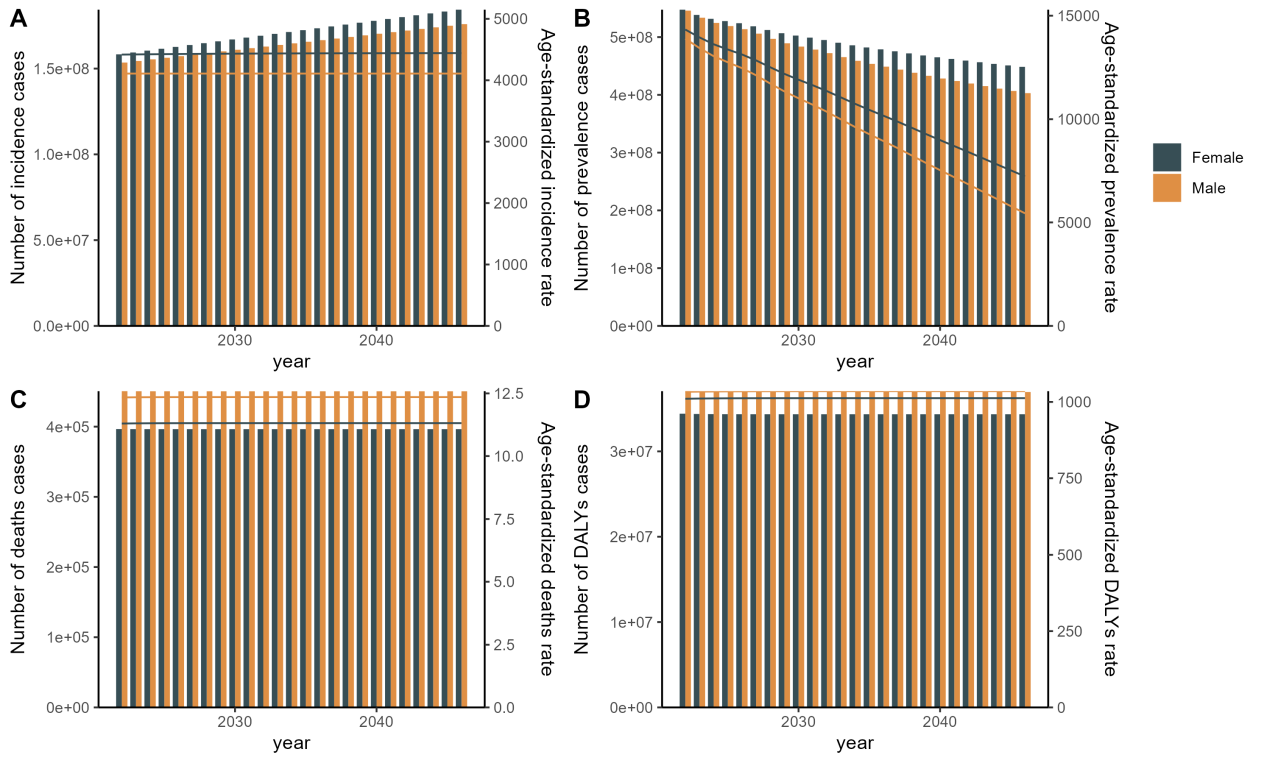


**Figure S12.** The predicted results in the neglected tropical diseases and malaria-related numbers and age-standardized rates of incidence, prevalence, deaths, and DALYs by sex globally from 2022 to 2046 of the ES model. Abbreviations: DALYs, disability-adjusted-life-year; ES, Exponential Smoothing.


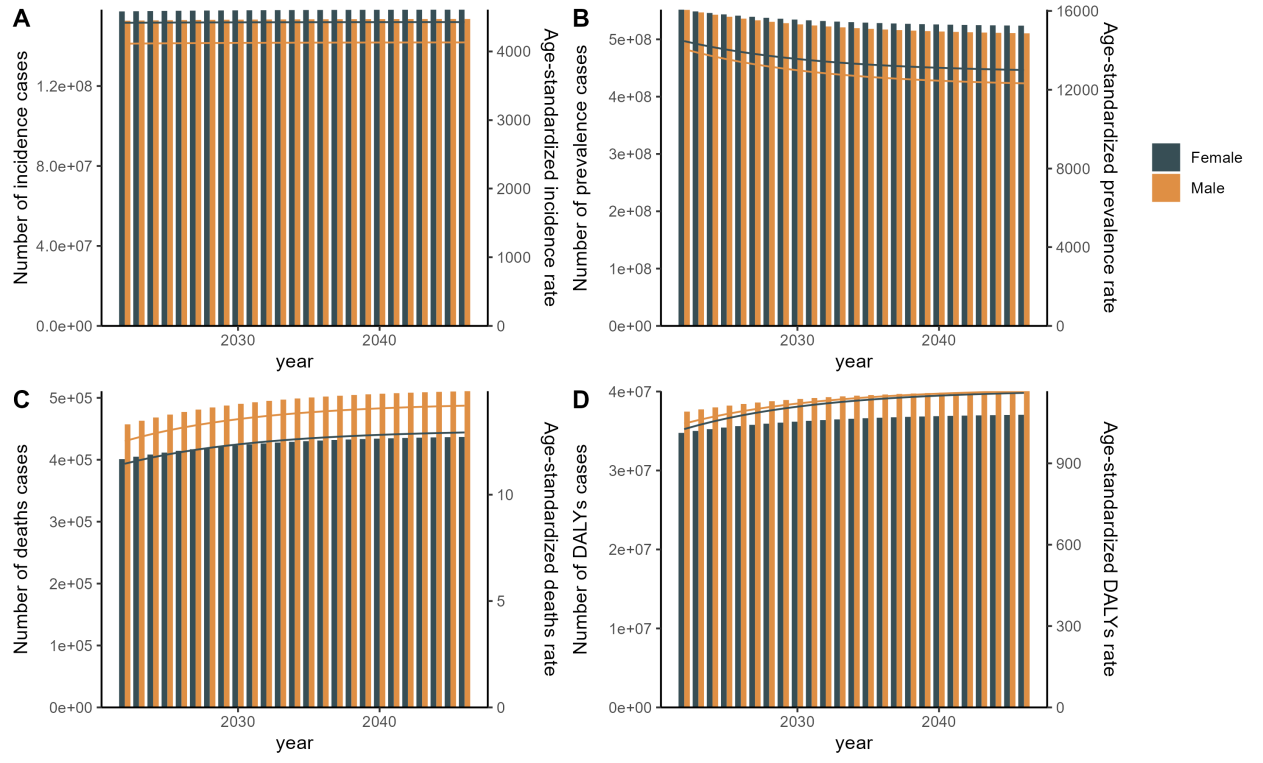


**Table S1.** The number of incidence cases and the age-standardized incidence rate of neglected tropical diseases and malaria in 1990 and 2021, and its trends from 1990 to 2021 globally.

|  | Number of incidence cases (95% UI) in 1990 | The age-standardized incidence rate/100000 (95% UI) in 1990 | Number of incidence cases (95% UI) in 2021 | The age-standardized incidence rate/100000 (95% UI) in 2021 | EAPC (95% CI) |
| --- | --- | --- | --- | --- | --- |
| Global | 247235621 (196271497-308015155) | 4235.42 (3347.35-5282.14) | 309802762 (232164372-398594668) | 4259.54 (3188.38-5488.91) | -0.11 (-0.24-0.03) |
| Sex |  |  |  |  |  |
| Female | 123586856 (97915826-153871825) | 4318.21 (3408.2-5376.64) | 157220092 (117871841-201998891) | 4415.78 (3316.59-5686.45) | -0.05 (-0.18-0.08) |
| Male | 123648765 (98099912-154041394) | 4160.2 (3281.41-5196.77) | 152582670 (113931362-196335745) | 4109.73 (3066.22-5295.97) | -0.17 (-0.31--0.03) |
| Age |  |  |  |  |  |
| <5 years | 90740510 (72948064-110715079) | 14637.03 (11766.99-17859.06) | 99958896 (68405899-141308700) | 15187.36 (10393.32-21469.88) | -0.33 (-0.62--0.04) |
| 5-9 years | 38775006 (23793312-56461352) | 6644.9 (4077.47-9675.81) | 46879680 (30977643-70123820) | 6823.29 (4508.77-10206.46) | 0.06 (-0.12-0.25) |
| 10-14 years | 30439871 (18863405-43513904) | 5682.44 (3521.38-8123.08) | 38684202 (25196292-57860687) | 5802.9 (3779.62-8679.5) | 0.07 (-0.02-0.16) |
| 15-19 years | 20518494 (14846752-26572924) | 3950.24 (2858.31-5115.85) | 28732074 (19819684-38341061) | 4604.65 (3176.33-6144.6) | 0.42 (0.3-0.55) |
| 20-24 years | 16357810 (11983951-21093504) | 3324.16 (2435.33-4286.53) | 22233118 (15054471-29956822) | 3723.15 (2521.02-5016.56) | 0.21 (0.11-0.32) |
| 25-29 years | 13137145 (9702839-16889221) | 2968.04 (2192.14-3815.74) | 17250288 (11400456-23326383) | 2932.01 (1937.72-3964.76) | 0 (-0.13-0.13) |
| 30-34 years | 9814569 (7235633-12687278) | 2546.44 (1877.33-3291.78) | 13303816 (8645763-18129369) | 2200.87 (1430.28-2999.17) | -0.18 (-0.35--0.01) |
| 35-39 years | 7528931 (5476966-9789188) | 2137.42 (1554.88-2779.09) | 10586532 (6612512-14670340) | 1887.53 (1178.98-2615.66) | -0.13 (-0.28-0.03) |
| 40-44 years | 5426638 (3923726-7082060) | 1894.24 (1369.63-2472.08) | 8082990 (4937301-11318494) | 1615.79 (986.97-2262.56) | -0.33 (-0.46--0.2) |
| 45-49 years | 4066030 (2929267-5305821) | 1751.12 (1261.55-2285.07) | 6074213 (3580035-8611663) | 1282.82 (756.07-1818.71) | -0.72 (-0.9--0.53) |
| 50-54 years | 3127621 (2211231-4114132) | 1471.33 (1040.23-1935.41) | 4833507 (2681634-7055817) | 1086.37 (602.72-1585.85) | -0.74 (-0.95--0.52) |
| 55-59 years | 2432146 (1678878-3226736) | 1313.25 (906.52-1742.3) | 3798734 (2001513-5675341) | 959.94 (505.78-1434.15) | -0.66 (-0.86--0.46) |
| 60-64 years | 1858592 (1237175-2505747) | 1157.21 (770.3-1560.15) | 3056349 (1503298-4693420) | 954.97 (469.71-1466.47) | -0.47 (-0.62--0.32) |
| 65-69 years | 1270382 (832404-1737681) | 1027.74 (673.41-1405.78) | 2294712 (1063186-3604309) | 831.89 (385.43-1306.66) | -0.18 (-0.45-0.08) |
| 70-74 years | 817737 (517951-1137136) | 965.89 (611.79-1343.16) | 1602541 (699697-2580596) | 778.54 (339.92-1253.7) | 0.08 (-0.22-0.38) |
| 75-79 years | 497407 (293146-720368) | 808.06 (476.23-1170.27) | 1077865 (445743-1773029) | 817.28 (337.98-1344.38) | 0.44 (0.2-0.69) |
| 80-84 years | 260025 (148152-383554) | 735.03 (418.79-1084.22) | 633708 (254444-1051088) | 723.55 (290.52-1200.1) | 0.58 (0.3-0.87) |
| 85-89 years | 109230 (56497-169118) | 722.85 (373.88-1119.17) | 350796 (136293-589376) | 767.24 (298.09-1289.05) | 0.83 (0.51-1.14) |
| 90-94 years | 40235 (16047-67761) | 938.93 (374.48-1581.27) | 213826 (80118-369478) | 1195.27 (447.85-2065.35) | 1.51 (1.11-1.9) |
| 95+ years | 17243 (4811-31961) | 1693.69 (472.6-3139.31) | 154914 (58805-270027) | 2842.3 (1078.93-4954.35) | 2.92 (2.25-3.6) |
| SDI region | |  |  |  |  |
| High-middle SDI | 1752924 (506445-4350161) | 165.51 (47.24-407.16) | 2656423 (1212396-4456010) | 228.66 (103.75-385.12) | 1.79 (1.3-2.29) |
| High SDI | 366279 (87281-828910) | 43.17 (10.45-97.52) | 612441 (143381-1323858) | 55.68 (13.21-120.1) | 1.64 (0.71-2.58) |
| Low-middle SDI | 83128191 (63460133-109897231) | 6225.08 (4664.04-8191.53) | 85029507 (54638828-114873962) | 4298.8 (2739.17-5827.21) | -1.4 (-1.51--1.29) |
| Low SDI | 125307873 (104293206-150049659) | 18412.63 (15475.62-21925.34) | 174026128 (141733552-217744842) | 12285.81 (10250.17-15104.74) | -1.46 (-1.6--1.32) |
| Middle SDI | 36444590 (20182537-54846732) | 2020.51 (1107.61-3067.77) | 47283393 (24245120-73741321) | 2054.45 (1062.49-3186.71) | 0.28 (0.13-0.43) |
| GBD region | |  |  |  |  |
| Advanced Health System | 432163 (145981-907446) | 33.86 (11.64-70.79) | 647245 (172620-1377974) | 41.92 (11.38-89.19) | 1.46 (0.61-2.32) |
| Africa | 163897323 (137303012-197984155) | 18816.05 (15826.28-22515.46) | 236205581 (186462342-301510123) | 13510.01 (10636.26-16969.08) | -1.42 (-1.62--1.22) |
| African Region | 159965500 (133702468-192815429) | 22502.96 (18826.42-26832.55) | 230880570 (183543860-294268621) | 15559.04 (12353.63-19521.2) | -1.49 (-1.69--1.3) |
| America | 13160399 (5167328-25328828) | 1792.01 (701.04-3455.62) | 18689018 (8733840-32812671) | 1841.06 (862.32-3222.99) | 0.96 (0.42-1.51) |
| Andean Latin America | 1250769 (689000-2683133) | 3400.33 (1914.52-6993.89) | 571001 (324286-859307) | 863.23 (489.08-1299.88) | -3.85 (-4.7--3) |
| Asia | 69823740 (39757383-108271886) | 2101.31 (1210.51-3202.34) | 54623828 (21203968-89072805) | 1207.5 (470.88-1969.81) | -1.51 (-1.72--1.31) |
| Australasia | 5843 (458-17138) | 28.92 (2.25-84.86) | 18539 (5727-43342) | 59.23 (18.31-139.69) | 3.73 (2.91-4.57) |
| Basic Health System | 21822146 (10634720-36058015) | 925.46 (450.31-1537.1) | 27270338 (16485015-40520964) | 897.06 (545.16-1325.32) | 0.76 (0.29-1.22) |
| Caribbean | 359965 (173980-704993) | 1000.7 (478.43-1995.04) | 460067 (191567-846981) | 970.61 (405.75-1780.34) | -0.63 (-1.68-0.42) |
| Central Africa | 29833498 (24295269-35690105) | 30570.59 (24931.68-37077.91) | 45894957 (36229674-58654579) | 20777.93 (16779.95-26699.78) | -1.66 (-1.92--1.39) |
| Central Asia | 61669 (26820-169640) | 87.8 (38.06-240.5) | 12066 (10031-14576) | 12.63 (10.49-15.22) | -9.71 (-12.56--6.76) |
| Central Europe | 5024 (4025-6788) | 4.04 (3.19-5.58) | 3568 (2997-4208) | 2.68 (2.25-3.15) | -1.52 (-1.66--1.39) |
| Central Latin America | 2337410 (1358506-3511073) | 1455.49 (861.28-2170.43) | 3847252 (2652667-5060099) | 1513.67 (1042.54-1993.34) | 1.65 (0.71-2.6) |
| Central Sub-Saharan Africa | 24124264 (19779392-29303032) | 30246.11 (25174.14-36862.5) | 38546874 (30369933-49439737) | 21343.78 (16917.26-27528.6) | -1.57 (-1.87--1.27) |
| Commonwealth High Income | 243687 (35033-618107) | 228.2 (32.72-578.97) | 536763 (135159-1174823) | 362.95 (92.19-792.24) | 2.33 (1.23-3.46) |
| Commonwealth Low Income | 41324895 (34176063-51336604) | 14351.55 (11831.6-17575.88) | 39558488 (29541306-50588924) | 8670.61 (6513.8-10977.58) | -2.19 (-2.43--1.95) |
| Commonwealth Middle Income | 110067932 (78547496-147542225) | 8179.3 (5762.64-10915.48) | 133588791 (81967192-185255991) | 6227.07 (3785.24-8653.12) | -0.96 (-1.08--0.84) |
| East Asia | 347385 (284783-462574) | 28.3 (23.1-37.95) | 97439 (50609-190056) | 6.52 (3.27-12.62) | -4.19 (-5.73--2.61) |
| East Asia & Pacific - WB | 11785774 (7187099-18434337) | 614.87 (374.72-961.49) | 10654015 (8511007-14025029) | 496.33 (399.56-651.92) | -0.3 (-0.57--0.02) |
| Eastern Africa | 37248012 (30542741-46019200) | 15709.43 (12865.49-19000.36) | 35509129 (27376046-43749904) | 7556.64 (5789.66-9190) | -2.89 (-3.26--2.51) |
| Eastern Europe | 10244 (8109-12541) | 4.09 (3.27-4.98) | 8830 (7042-10852) | 3.48 (2.77-4.22) | -0.46 (-0.53--0.39) |
| Eastern Mediterranean Region | 14083263 (7762641-29050888) | 3269.12 (1835.16-6390.27) | 15136698 (10478802-20812467) | 1914.61 (1322.36-2652.97) | -2.06 (-2.63--1.49) |
| Eastern Sub-Saharan Africa | 51562416 (42773870-63010735) | 19403.34 (16066.81-23473.09) | 53506607 (40201325-66470862) | 9991.61 (7544.76-12327.38) | -2.62 (-2.93--2.3) |
| Europe | 50761 (39372-67539) | 6.52 (4.97-8.78) | 30490 (22081-44368) | 3.57 (2.45-5.36) | -3.53 (-4.45--2.61) |
| Europe & Central Asia - WB | 109505 (70171-211366) | 13.46 (8.59-26.14) | 41200 (32364-54911) | 4.56 (3.51-6.1) | -6 (-7.99--3.97) |
| European Region | 109682 (70329-211581) | 13.36 (8.54-25.92) | 41618 (32768-55346) | 4.55 (3.52-6.06) | -5.97 (-7.95--3.95) |
| High-income Asia Pacific | 221084 (34290-574329) | 131.22 (20.29-341) | 486526 (108229-1124305) | 294.4 (65.36-679.88) | 3.47 (2.31-4.64) |
| High-income North America | 1051 (715-1793) | 0.36 (0.24-0.62) | 2337 (884-8144) | 0.59 (0.22-2.09) | 3 (2.25-3.76) |
| Latin America & Caribbean - WB | 13225732 (5169268-25542929) | 2953.24 (1138.81-5738.25) | 18744151 (8733082-32944316) | 2830.75 (1318.8-4970.68) | 0.75 (0.2-1.3) |
| Limited Health System | 169018813 (134433903-213072476) | 9042.06 (7078.73-11330.08) | 194836159 (134873162-255995950) | 6394.38 (4370.66-8474.32) | -1.34 (-1.47--1.2) |
| Middle East & North Africa - WB | 2507324 (1134713-5547626) | 855.32 (432.82-1710.27) | 2945754 (2430562-3549065) | 614.75 (509.76-738.67) | -1.71 (-3.11--0.28) |
| Minimal Health System | 55726735 (47472388-66831938) | 28697.46 (24768.85-34020.22) | 86854149 (71931087-108122593) | 18785.57 (15646.67-23011.75) | -1.55 (-1.65--1.46) |
| North Africa and Middle East | 5789756 (4080592-8782551) | 1470.32 (1077.26-2120.33) | 6892718 (4939891-9703021) | 1082.15 (776.82-1522.47) | -2.09 (-3.14--1.03) |
| North America | 1051 (715-1793) | 0.36 (0.24-0.63) | 2337 (885-8144) | 0.59 (0.22-2.09) | 3 (2.25-3.76) |
| Northern Africa | 171546 (122549-242539) | 144.71 (105.31-207.02) | 247759 (197179-363165) | 119.32 (95.2-174.44) | -0.27 (-1.2-0.67) |
| Oceania | 2611969 (1320662-5203702) | 32250.79 (17729.83-61124.91) | 2521041 (2219132-2861462) | 16903.94 (15062.7-19041.16) | -2.83 (-3.38--2.27) |
| Region of the Americas | 13160399 (5167328-25328828) | 1792.01 (701.04-3455.62) | 18689018 (8733840-32812671) | 1841.06 (862.32-3222.99) | 0.96 (0.42-1.51) |
| South-East Asia Region | 54543773 (28283015-87945277) | 4078.68 (2186.52-6371.02) | 38509652 (7807269-71607211) | 1861.5 (375.21-3467.31) | -2.27 (-2.46--2.07) |
| South Asia | 53828891 (26906368-88258203) | 4767.75 (2509.73-7492.11) | 40364422 (7982405-75444582) | 2181.14 (426.81-4083.24) | -2.27 (-2.47--2.07) |
| South Asia - WB | 55642917 (28641235-90163753) | 4812.69 (2602.94-7481.36) | 41287403 (9112213-76136863) | 2168.66 (474.72-4004.18) | -2.31 (-2.51--2.11) |
| Southeast Asia | 9706478 (5930266-15272128) | 2061.7 (1292.73-3194.25) | 8180948 (6199569-11830368) | 1174.02 (888.84-1694.4) | -1.27 (-1.69--0.85) |
| Southern Africa | 18357967 (14943679-22276788) | 14503.67 (11853.98-17319.89) | 26456299 (19249958-35710163) | 11744.9 (8604.43-15838.02) | -1.17 (-1.43--0.91) |
| Southern Latin America | 97828 (55657-191399) | 193.72 (109.26-382.55) | 106047 (43309-212597) | 158.32 (65.01-316.28) | 0.01 (-0.66-0.68) |
| Southern Sub-Saharan Africa | 935940 (676645-1454041) | 1648 (1218.43-2461.85) | 640576 (494207-830476) | 774.51 (598.98-1001.99) | -0.28 (-1.64-1.1) |
| Sub-Saharan Africa - WB | 163727428 (137117792-197851154) | 22903.55 (19255.03-27393.41) | 235932956 (186208888-301253960) | 15557.47 (12249.77-19469.49) | -1.58 (-1.77--1.39) |
| Tropical Latin America | 9191953 (2559715-20407928) | 5860.82 (1570.59-13188.83) | 13775360 (4687172-27053822) | 6098.34 (2080.34-11958.45) | 0.84 (0.34-1.34) |
| Western Africa | 78286301 (63157029-94626249) | 31960.11 (26086.93-38784.48) | 128097436 (100253875-163001380) | 22110.69 (17437.27-28252.3) | -1.39 (-1.56--1.22) |
| Western Europe | 4541 (3852-5390) | 1.17 (0.99-1.4) | 4001 (3472-4633) | 0.84 (0.73-0.98) | -0.81 (-0.91--0.71) |
| Western Pacific Region | 5036319 (3299821-7859475) | 321.38 (209.34-507.13) | 6256195 (4613130-10287757) | 382.24 (283.82-625.38) | 0.68 (0.32-1.05) |
| Western Sub-Saharan Africa | 84781140 (68654948-101555553) | 31188.78 (25617.34-37455.44) | 139756542 (108312774-176522780) | 21439.08 (16894.56-27582.1) | -1.43 (-1.59--1.27) |
| World Bank High Income | 427326 (123237-933328) | 43.33 (12.63-94.3) | 714273 (220838-1471230) | 58.76 (18.64-119.85) | 1.84 (0.96-2.73) |
| World Bank Low Income | 83484307 (71238965-101010264) | 19376.32 (16630.61-23254.37) | 116553550 (96585786-143843072) | 13144.19 (10852.11-16040.46) | -1.41 (-1.58--1.25) |
| World Bank Lower Middle Income | 148204822 (113658142-189591057) | 6412.7 (4836.37-8201.27) | 172201004 (116913507-231171622) | 4936.04 (3329.75-6636.33) | -1.07 (-1.19--0.95) |
| World Bank Upper Middle Income | 14883346 (6632932-26840184) | 733.71 (324.35-1328.4) | 20139044 (10169788-34154207) | 844.01 (430.5-1428.12) | 1.52 (0.97-2.07) |
| Country |  |  |  |  |  |
| Afghanistan | 896062 (660559-1189523) | 8647.8 (6629.82-11043.22) | 422577 (336600-531469) | 1431.72 (1156.63-1798.6) | -4.04 (-5.14--2.93) |
| Albania | 301 (185-476) | 8.52 (5.37-13.21) | 83 (61-110) | 3.29 (2.35-4.62) | -4.55 (-5.47--3.63) |
| Algeria | 17948 (8728-34327) | 71.73 (35.15-139.52) | 44316 (29213-66132) | 102.27 (67.38-151.39) | 2.64 (0.34-4.99) |
| American Samoa | 972 (197-3447) | 2010.75 (406.62-7126.78) | 1140 (251-3839) | 2283.6 (502.85-7692.83) | 0.68 (-0.04-1.41) |
| Andorra | 0 (0-0) | 0.56 (0.44-0.7) | 1 (0-1) | 0.54 (0.43-0.68) | -0.13 (-0.16--0.11) |
| Angola | 3375347 (1998287-4915012) | 23215.45 (15030.52-32616.04) | 8026433 (4798072-12014825) | 17898.47 (11005.59-26804.01) | -1.95 (-2.57--1.32) |
| Antigua and Barbuda | 30 (2-91) | 49.28 (2.97-149.5) | 74 (9-267) | 82.99 (10.51-297.55) | 0.76 (-2.76-4.41) |
| Argentina | 83737 (42738-177003) | 249.75 (126.22-531.13) | 104211 (41436-210671) | 229.67 (91.85-462.87) | 0.37 (-0.24-0.99) |
| Armenia | 1017 (541-1891) | 30.18 (16.17-55.32) | 338 (275-409) | 10.64 (8.64-12.93) | -4.1 (-6.22--1.92) |
| Australia | 5817 (430-17111) | 34.67 (2.55-102.01) | 18491 (5681-43288) | 71.1 (21.88-167.92) | 3.73 (2.91-4.57) |
| Austria | 72 (49-126) | 0.94 (0.58-1.82) | 47 (33-81) | 0.53 (0.33-1.04) | -2.12 (-2.4--1.83) |
| Azerbaijan | 916 (571-1562) | 12.76 (7.93-21.2) | 513 (407-624) | 4.73 (3.73-5.78) | -10.92 (-14.93--6.71) |
| Bahamas | 3005 (9-10036) | 1160.93 (3.4-3877.24) | 5378 (62-18340) | 1393.03 (16.1-4752.14) | 0.81 (0.51-1.11) |
| Bahrain | 1 (1-1) | 0.22 (0.19-0.27) | 2 (1-2) | 0.1 (0.08-0.13) | -2.62 (-2.72--2.52) |
| Bangladesh | 1665448 (445921-4432742) | 1649.68 (453.08-4225.55) | 759792 (173313-1420991) | 461.16 (104.92-862.92) | -4.01 (-4.5--3.52) |
| Barbados | 3638 (187-10576) | 1424.54 (73-4140.92) | 5815 (726-14678) | 1950.75 (243.09-4927.69) | 2.23 (0.68-3.8) |
| Belarus | 749 (584-915) | 6.49 (5.11-7.84) | 717 (561-899) | 6.17 (4.9-7.57) | -0.17 (-0.21--0.13) |
| Belgium | 5 (3-7) | 0.04 (0.03-0.06) | 8 (6-11) | 0.06 (0.04-0.09) | 0.71 (-0.12-1.55) |
| Belize | 15423 (190-116700) | 6615.19 (111.71-45923.75) | 2553 (333-8146) | 592.94 (78.28-1888.34) | -8.14 (-10.41--5.82) |
| Benin | 2254346 (1668277-2804247) | 31406.8 (23656.71-38343.19) | 5256335 (3528016-6879263) | 27886.18 (19039.4-36564.29) | -0.34 (-0.58--0.09) |
| Bermuda | 0 (0-1) | 0.68 (0.54-0.84) | 1 (0-1) | 0.69 (0.52-0.95) | 2.23 (0.22-4.27) |
| Bhutan | 632969 (227798-1408645) | 76445.84 (29879.54-166825.43) | 470 (86-1185) | 62.1 (11.09-156.94) | -21.61 (-22.77--20.43) |
| Bolivia (Plurinational State of) | 196756 (106421-340346) | 3183.24 (1726.75-5464.42) | 115786 (48035-220344) | 980.77 (404.88-1869.62) | -3.46 (-3.95--2.96) |
| Bosnia and Herzegovina | 397 (312-503) | 8.75 (6.88-11.13) | 228 (183-278) | 6.22 (5.02-7.61) | -1.02 (-1.25--0.79) |
| Botswana | 854 (745-985) | 73.86 (64.54-85.2) | 2951 (2521-3433) | 122.39 (104.54-142.38) | -4.12 (-6.91--1.24) |
| Brazil | 9124014 (2540791-20322423) | 5975.27 (1602.11-13488.49) | 13593356 (4568145-26846552) | 6219.91 (2095.29-12271.35) | 0.85 (0.34-1.35) |
| Brunei Darussalam | 119 (8-396) | 46.3 (3.29-153.64) | 574 (151-1427) | 130.33 (34.5-322.67) | 4.67 (3.55-5.8) |
| Bulgaria | 747 (561-1056) | 9.06 (6.5-13.32) | 329 (296-370) | 4.44 (3.91-5.03) | -2.18 (-2.33--2.02) |
| Burkina Faso | 5854289 (4750522-7344709) | 40837.87 (32993.9-50804.6) | 8634755 (5441929-12419255) | 26936.79 (17991.7-37351.8) | -1.52 (-1.83--1.21) |
| Burundi | 2649021 (1788214-3226391) | 32137.44 (22835.74-39322.56) | 3828175 (2270578-5282454) | 21330.74 (13943.22-29835.14) | -2.8 (-3.69--1.9) |
| Cabo Verde | 21188 (11-111005) | 5894.22 (3.46-30877.9) | 43368 (153-246809) | 7761.43 (27.1-44171.85) | 1.06 (0.73-1.39) |
| Cambodia | 740084 (624393-870648) | 7416.42 (6349.42-8603.14) | 58472 (25490-108477) | 344.92 (150.22-640.2) | -6.54 (-7.56--5.51) |
| Cameroon | 4553372 (3663039-5471326) | 29925.57 (23469.43-36758.29) | 7907532 (5013679-11750120) | 19228.36 (12393.13-28004.71) | -1.92 (-2.25--1.59) |
| Canada | 17 (12-24) | 0.06 (0.04-0.08) | 22 (15-31) | 0.05 (0.04-0.08) | -0.49 (-0.83--0.14) |
| Central African Republic | 1187231 (726536-1557788) | 30559.7 (20126.74-40089.55) | 1684429 (915038-2669323) | 23528.82 (13264.91-36976.92) | -0.85 (-1.06--0.64) |
| Chad | 1857742 (1183673-2668007) | 21327.08 (14193.15-30147.13) | 3632410 (2115990-5870413) | 14202.4 (8578.58-21951.97) | -1.57 (-1.78--1.35) |
| Chile | 12947 (11404-14796) | 90.04 (79.61-102.96) | 1605 (1414-1848) | 7.77 (6.82-8.87) | -7.85 (-9.52--6.15) |
| China | 313932 (268708-402878) | 26.37 (22.57-33.94) | 54357 (33365-110536) | 3.77 (2.18-7.94) | -5.62 (-6.73--4.5) |
| Colombia | 650644 (363494-1115383) | 2050.96 (1170.4-3471.67) | 887311 (325035-1651726) | 1792.48 (652.42-3342.78) | 0.43 (-0.94-1.81) |
| Comoros | 162343 (21996-619907) | 29256.4 (4882.15-111795.25) | 100730 (10567-602300) | 13536.79 (1444.04-80521.63) | -2.44 (-3.33--1.54) |
| Congo | 869936 (621361-1184550) | 26684.1 (19359.35-35694.72) | 1187620 (760359-1837542) | 19138.53 (12171.64-29828) | -1.67 (-2.04--1.29) |
| Cook Islands | 53 (6-190) | 278.18 (29.05-987.55) | 19 (1-77) | 105.29 (3.65-431.69) | 3.86 (1.38-6.4) |
| Costa Rica | 84754 (17743-191135) | 2778.49 (586.95-6252.29) | 133712 (24356-321349) | 2814.37 (513.43-6760.91) | 1.13 (-0.31-2.6) |
| Croatia | 215 (175-257) | 4.08 (3.38-4.96) | 163 (140-190) | 3.32 (2.81-3.91) | -0.53 (-0.62--0.44) |
| Cuba | 10840 (811-36841) | 100.22 (7.49-340.64) | 24289 (6817-60441) | 214.21 (59.89-534.46) | 3.71 (1.82-5.64) |
| Cyprus | 8 (3-16) | 1.02 (0.41-2.22) | 9 (5-16) | 0.7 (0.38-1.3) | -1 (-1.33--0.67) |
| Czechia | 60 (46-76) | 0.53 (0.4-0.67) | 64 (49-82) | 0.5 (0.39-0.64) | -0.05 (-0.17-0.07) |
| C么te d'Ivoire | 6552440 (5287707-8183284) | 36170.25 (29544.56-45579) | 7632582 (3793521-11334799) | 21081.14 (10884.21-30813.47) | -2.26 (-2.79--1.73) |
| Democratic People's Republic of Korea | 165 (130-206) | 0.82 (0.65-1.01) | 7918 (6213-9927) | 27.64 (21.68-34.64) | 13.33 (4.17-23.29) |
| Democratic Republic of the Congo | 18216129 (14689545-22954247) | 32483.62 (26149.35-41135.51) | 26902504 (20542249-34862272) | 22749.22 (17336.48-28850.52) | -1.48 (-1.78--1.17) |
| Denmark | 1 (1-2) | 0.02 (0.01-0.03) | 1 (1-2) | 0.02 (0.01-0.03) | -0.71 (-1.56-0.14) |
| Djibouti | 54831 (13406-131138) | 13252.98 (3349.55-31518.99) | 135999 (119799-177885) | 10568.7 (9323.58-13918.64) | -4.62 (-8.3--0.79) |
| Dominica | 95 (4-301) | 129.19 (5.29-410.64) | 161 (17-441) | 238.84 (24.46-655.63) | 3.63 (0.15-7.24) |
| Dominican Republic | 27396 (2287-82732) | 382.83 (33.55-1150.39) | 62706 (13567-142782) | 571.31 (123.69-1300.74) | 3.05 (1.44-4.69) |
| Ecuador | 232494 (178118-297836) | 2450.09 (1897.04-3110.25) | 109656 (36724-210649) | 607.18 (203.43-1166.84) | -4.37 (-6.06--2.64) |
| Egypt | 74991 (34963-151024) | 134.71 (63.04-277.82) | 33302 (8176-144709) | 31.81 (7.93-137.71) | -4.3 (-5.35--3.23) |
| El Salvador | 105194 (48049-208213) | 2047.1 (968.68-3966.06) | 108506 (25426-242604) | 1664.33 (389.92-3717.93) | 2.41 (1.04-3.8) |
| Equatorial Guinea | 137426 (83332-186708) | 22278.23 (13872.29-29883.64) | 322920 (152329-505565) | 17169.08 (8145.81-26706.29) | -0.58 (-0.81--0.36) |
| Eritrea | 81579 (24312-218384) | 2546.78 (773.96-6388.43) | 45582 (42565-50573) | 734.02 (686.45-811.05) | -3.09 (-5.34--0.78) |
| Estonia | 25 (20-32) | 1.41 (1.11-1.77) | 21 (17-28) | 1.31 (1.02-1.67) | -0.26 (-0.29--0.23) |
| Eswatini | 9005 (818-35889) | 1217.11 (119.11-4549.62) | 1350 (1063-1672) | 122.37 (96.44-151.54) | -9.68 (-11.23--8.11) |
| Ethiopia | 1308395 (862448-2017625) | 2778.2 (1832.39-4219.52) | 3247644 (2662617-3827574) | 3087.86 (2549.33-3630.65) | 1.48 (-0.85-3.87) |
| Fiji | 8114 (866-22590) | 1064.91 (114.15-2963.93) | 15401 (4513-35136) | 1682.81 (494.88-3824.93) | 1.65 (1.13-2.17) |
| Finland | 1 (1-2) | 0.02 (0.01-0.04) | 1 (1-2) | 0.02 (0.01-0.03) | -0.98 (-1.33--0.63) |
| France | 223 (125-370) | 0.42 (0.24-0.69) | 157 (143-174) | 0.24 (0.21-0.28) | -1.73 (-1.86--1.59) |
| Gabon | 338194 (210587-484950) | 26510.88 (17843-37580.41) | 422968 (198884-735756) | 20382.89 (9731.96-35569.93) | -1.29 (-2.11--0.47) |
| Gambia | 152646 (119068-193161) | 12985.1 (10591.46-15962.61) | 166603 (136978-224715) | 6747.94 (5625-9212.12) | 0.09 (-1.44-1.65) |
| Georgia | 837 (538-1370) | 15.07 (9.62-24.78) | 339 (279-423) | 9.27 (7.37-11.97) | -1.21 (-3.24-0.86) |
| Germany | 17 (10-26) | 0.02 (0.01-0.03) | 33 (22-49) | 0.03 (0.02-0.05) | 1.92 (1.28-2.56) |
| Ghana | 7412338 (5602294-8928240) | 35596.01 (26711.81-42836.62) | 5951111 (4096352-8453029) | 14727.65 (10261.8-21249.47) | -2.64 (-3.17--2.1) |
| Greece | 162 (112-234) | 1.69 (1.11-2.51) | 197 (155-245) | 1.96 (1.48-2.57) | 1.07 (0.85-1.29) |
| Greenland | 0 (0-0) | 0.02 (0.01-0.04) | 0 (0-0) | 0.02 (0.01-0.03) | -0.03 (-0.16-0.1) |
| Grenada | 53 (2-168) | 60.42 (1.93-189) | 347 (112-902) | 341.52 (109.78-888) | 7.21 (5.32-9.14) |
| Guam | 3 (3-4) | 2.7 (2.31-3.19) | 3 (3-4) | 1.88 (1.67-2.13) | -1.07 (-1.29--0.86) |
| Guatemala | 210323 (157953-265116) | 2721.83 (2072.21-3384.19) | 47924 (17216-90797) | 302.34 (108.95-572.55) | -5.73 (-7.55--3.87) |
| Guinea | 2755971 (2127765-3558345) | 32217.28 (24976.4-41872.06) | 4439699 (2345229-6501761) | 24136.71 (13433.41-34455.92) | -0.87 (-1.12--0.62) |
| Guinea-Bissau | 428652 (263271-640960) | 29219.32 (19147.3-43437.86) | 226144 (72569-501097) | 8772.81 (2821.56-18975.1) | -5.91 (-6.99--4.83) |
| Guyana | 108738 (87454-133417) | 13397.02 (10990.87-16091.27) | 93972 (77458-113204) | 12135.79 (10019.75-14597.3) | -3.19 (-4.6--1.77) |
| Haiti | 77809 (43933-170236) | 1308.38 (754.6-2768.49) | 174832 (37568-435148) | 1383.36 (301.21-3399.37) | -2.55 (-3.87--1.22) |
| Honduras | 190516 (137806-252794) | 4227.82 (3131.66-5528.03) | 102565 (21953-264761) | 1013.39 (219.29-2611.28) | -4.1 (-5.62--2.56) |
| Hungary | 153 (120-186) | 1.34 (1.05-1.63) | 138 (108-170) | 1.24 (0.98-1.54) | -0.26 (-0.28--0.24) |
| Iceland | 0 (0-0) | 0.02 (0.01-0.03) | 0 (0-0) | 0.02 (0.01-0.03) | -0.72 (-1.41--0.02) |
| India | 44366523 (20566859-76124745) | 5060.31 (2444.94-8331.21) | 32606419 (3601581-64155234) | 2300.87 (252.13-4526.84) | -2.34 (-2.54--2.13) |
| Indonesia | 4646764 (1591540-9644716) | 2397.98 (853.01-4946.95) | 3678649 (2136493-6338554) | 1319.23 (759.46-2284.28) | -1.89 (-2.16--1.62) |
| Iran (Islamic Republic of) | 358339 (274106-472667) | 638.65 (497.83-841.52) | 59388 (40487-82930) | 71.63 (48.63-101.4) | -6.44 (-7.43--5.44) |
| Iraq | 41055 (31257-54281) | 221.36 (169.76-291.17) | 78469 (52756-109916) | 194.61 (131.55-275.1) | -1.91 (-4.79-1.07) |
| Ireland | 1 (0-1) | 0.02 (0.01-0.03) | 1 (0-1) | 0.02 (0.01-0.03) | -0.47 (-0.51--0.44) |
| Israel | 92 (54-164) | 1.85 (1.11-3.26) | 357 (270-461) | 3.67 (2.77-4.76) | 3.03 (2.68-3.38) |
| Italy | 2252 (1852-2730) | 3.76 (3.06-4.6) | 1692 (1454-1982) | 2.51 (2.13-3) | -1.48 (-1.62--1.34) |
| Jamaica | 2140 (33-7081) | 88.79 (1.41-293.72) | 4422 (1001-10118) | 156.03 (35.31-357.23) | 3.36 (-0.38-7.25) |
| Japan | 118 (71-185) | 0.08 (0.05-0.13) | 134 (84-216) | 0.08 (0.05-0.13) | -0.09 (-0.24-0.05) |
| Jordan | 888 (362-1957) | 23.43 (9.55-51.92) | 2922 (1047-7547) | 23.89 (8.67-61.49) | 3.11 (0.75-5.52) |
| Kazakhstan | 1255 (778-2095) | 7.86 (4.95-12.95) | 1063 (865-1315) | 5.56 (4.52-6.84) | -0.5 (-0.8--0.2) |
| Kenya | 6186823 (4662103-8054990) | 19030.37 (14343.88-25292.17) | 3327784 (2399439-4444913) | 5767.26 (4175.98-7723.34) | -5.47 (-6.27--4.66) |
| Kiribati | 4420 (390-16909) | 5968.46 (530.08-22812.58) | 7419 (532-29497) | 6138.83 (442.86-24397.71) | -0.17 (-0.45-0.11) |
| Kuwait | 1786 (304-5320) | 105.8 (18.21-312.15) | 2825 (420-9452) | 62.49 (8.79-210.36) | 0.65 (-1.68-3.03) |
| Kyrgyzstan | 1295 (704-2463) | 29.83 (16.54-56.19) | 813 (650-1048) | 11.98 (9.66-15.32) | -1.56 (-3.9-0.83) |
| Lao People's Democratic Republic | 79629 (60509-101556) | 2088.88 (1609.53-2632.44) | 32440 (14609-57794) | 443.32 (199.9-791.27) | -6.08 (-7.4--4.75) |
| Latvia | 95 (73-117) | 3.1 (2.43-3.8) | 71 (58-86) | 2.94 (2.38-3.5) | -0.14 (-0.19--0.09) |
| Lebanon | 1436 (700-3032) | 48.03 (23.68-100.15) | 2278 (582-6596) | 40.86 (10.35-119.26) | 1.87 (-0.25-4.04) |
| Lesotho | 10 (8-13) | 0.74 (0.62-0.91) | 11 (9-13) | 0.62 (0.51-0.74) | -0.47 (-0.63--0.3) |
| Liberia | 1161534 (677191-1599554) | 32557.52 (18988.44-44829.13) | 1963193 (1007824-2865881) | 28266.95 (14836.68-41234.36) | -0.72 (-1.05--0.39) |
| Libya | 5702 (2449-12023) | 133.66 (56.74-275.21) | 10906 (6737-16402) | 168.38 (104.91-257.5) | 1.91 (0.64-3.2) |
| Lithuania | 188 (153-228) | 4.67 (3.79-5.66) | 165 (141-194) | 4.58 (3.88-5.43) | 0 (-0.14-0.13) |
| Luxembourg | 0 (0-0) | 0.02 (0.01-0.03) | 0 (0-0) | 0.02 (0.01-0.03) | -0.4 (-0.98-0.18) |
| Madagascar | 2733469 (2044545-3462607) | 16544.54 (12493.16-20974.09) | 2729962 (1498434-4100823) | 7838.66 (4372.03-11392.54) | -3.53 (-4.16--2.9) |
| Malawi | 4623717 (3673857-5692873) | 32275.25 (26278.37-39699) | 4294979 (2795867-7150683) | 17265.25 (11383.22-28202.26) | -2.5 (-2.82--2.19) |
| Malaysia | 270062 (122283-557611) | 1563.46 (729.02-3184.82) | 816294 (308355-2067888) | 2586.99 (977.08-6553.24) | 3.59 (2.55-4.64) |
| Maldives | 771 (3-3464) | 346.48 (1.9-1557.37) | 25898 (3-93395) | 5095.67 (0.46-18144.28) | 10.53 (8.98-12.09) |
| Mali | 4203849 (3370664-5393242) | 33131.44 (26863.23-42528.7) | 6780152 (4422678-10067836) | 19727.53 (13557.95-28843.02) | -1.66 (-1.97--1.36) |
| Malta | 13 (7-24) | 4 (1.94-7.08) | 8 (5-12) | 2.1 (1.28-3.3) | -2.35 (-2.76--1.94) |
| Marshall Islands | 4018 (420-23864) | 8676.5 (912.95-51292.45) | 5804 (603-27804) | 10341.75 (1077.13-49413.95) | 0.8 (0.36-1.25) |
| Mauritania | 56482 (42828-74761) | 3084.81 (2410.26-3971.05) | 111135 (90790-136499) | 2666.3 (2201.67-3237.56) | -0.69 (-2.76-1.44) |
| Mauritius | 3159 (324-11225) | 288.44 (29.55-1033.36) | 12716 (351-51037) | 1009.55 (27.82-4045.04) | 3.85 (3.39-4.31) |
| Mexico | 652046 (208383-1188914) | 769.57 (252.8-1395.96) | 1464441 (1061095-1898466) | 1135.68 (823.02-1472.61) | 3.04 (1.67-4.43) |
| Micronesia (Federated States of) | 501 (69-1490) | 484.97 (74.49-1424.16) | 466 (86-1355) | 455.34 (84.61-1321.06) | -0.37 (-0.68--0.05) |
| Monaco | 0 (0-0) | 0.46 (0.2-1) | 1 (0-4) | 5.27 (1.24-15.67) | 9.8 (7.6-12.05) |
| Mongolia | 70 (55-89) | 3.65 (2.93-4.5) | 105 (82-130) | 3.21 (2.55-3.89) | -0.5 (-0.54--0.45) |
| Montenegro | 14 (11-18) | 2.26 (1.78-2.82) | 14 (11-20) | 2.4 (1.7-3.6) | 0.08 (-0.35-0.51) |
| Morocco | 7886 (6097-10173) | 33.06 (25.41-42.99) | 23765 (11237-47763) | 65.27 (30.5-130.8) | 4.21 (2.92-5.52) |
| Mozambique | 6378972 (5266986-7673439) | 33482.62 (27684.1-39919.21) | 9995895 (7096454-13095179) | 23176.82 (16854.65-29837.39) | -1.56 (-1.74--1.38) |
| Myanmar | 923861 (748982-1119961) | 2371.49 (1932.95-2861.19) | 248119 (198592-313076) | 436.79 (349.07-552.07) | -2.84 (-4.69--0.95) |
| Namibia | 132822 (12460-583091) | 8257.49 (980.87-31902.2) | 24291 (19936-29330) | 1026.72 (843.57-1238.27) | -8.67 (-12.71--4.45) |
| Nauru | 4 (0-17) | 41.82 (4.97-172.96) | 118 (0-496) | 1070.75 (2.67-4469.5) | 14.55 (9.87-19.44) |
| Nepal | 245896 (122322-427604) | 1339.93 (696.73-2279.7) | 281396 (7166-818319) | 902.97 (23.58-2624.99) | -0.92 (-1.44--0.4) |
| Netherlands | 3 (2-5) | 0.02 (0.01-0.03) | 4 (2-6) | 0.02 (0.01-0.03) | -0.34 (-0.38--0.3) |
| New Zealand | 27 (21-32) | 0.73 (0.58-0.9) | 49 (39-60) | 0.76 (0.61-0.94) | 0 (-0.07-0.08) |
| Nicaragua | 118301 (88820-157103) | 3242.04 (2487.5-4232.3) | 249072 (79365-586611) | 3737.83 (1206.3-8754.41) | -1.82 (-3.81-0.21) |
| Niger | 3252260 (1753214-4696361) | 27450.66 (16216.87-38588.64) | 8015854 (4509692-11501286) | 21407.7 (13076.38-30338.14) | -0.43 (-0.81--0.06) |
| Nigeria | 39793369 (31045359-49489118) | 32398.28 (25685.11-40563.81) | 73023408 (51697262-98274464) | 23717.85 (17412.11-31364.69) | -1.32 (-1.54--1.11) |
| Niue | 2 (0-7) | 81.49 (15.06-289.6) | 2 (0-5) | 117.17 (29.35-328.53) | 1.55 (1.07-2.02) |
| North Macedonia | 71 (49-107) | 3.58 (2.48-5.47) | 52 (40-70) | 2.44 (1.84-3.37) | -1.22 (-1.44--0.99) |
| Northern Mariana Islands | 274 (38-858) | 616.87 (85.14-1928.29) | 361 (38-1376) | 758 (80.06-2896.34) | 0.65 (-0.54-1.86) |
| Norway | 26 (21-33) | 0.55 (0.43-0.7) | 37 (30-46) | 0.59 (0.46-0.73) | 0.19 (0.08-0.29) |
| Oman | 5179 (1151-16495) | 283.63 (62.04-899.78) | 1481 (218-6750) | 33.17 (4.85-144.29) | -7.64 (-11.25--3.89) |
| Pakistan | 6918056 (1393660-20807921) | 5650.68 (1279.18-15271.12) | 6716346 (3519714-11486231) | 2833.78 (1468.2-4864.94) | -1.74 (-2.27--1.21) |
| Palau | 512 (10-2922) | 3365.29 (64.24-19189.29) | 573 (2-3070) | 3260.74 (8.25-17475.52) | -1.02 (-1.98--0.05) |
| Palestine | 1016 (501-1846) | 49.11 (24.84-90.23) | 1927 (798-4819) | 37.8 (15.89-94.13) | 1.07 (-0.54-2.71) |
| Panama | 17028 (6024-38656) | 722.88 (262.88-1619.6) | 61157 (29038-152658) | 1417.15 (673.66-3533.75) | 2.84 (1.3-4.41) |
| Papua New Guinea | 2166756 (1014174-4611067) | 42508.45 (21947.96-84720.43) | 2144975 (1846663-2470729) | 19204.83 (16777.51-21810.59) | -3.23 (-3.79--2.67) |
| Paraguay | 67939 (14587-188610) | 1704.26 (388.54-4662.35) | 182004 (50544-382479) | 2531.74 (703.22-5330.77) | 1.58 (0.91-2.25) |
| Peru | 821519 (313904-2240074) | 3897.57 (1526.51-10147.32) | 345558 (170333-665789) | 950.54 (468.98-1837.58) | -3.63 (-4.73--2.52) |
| Philippines | 396421 (275514-557914) | 595.9 (382.89-864.56) | 1277615 (315022-4501071) | 1124.31 (278.64-3949.36) | 2.84 (1.89-3.79) |
| Poland | 685 (546-828) | 1.72 (1.37-2.09) | 826 (655-1015) | 1.73 (1.39-2.1) | 0.08 (-0.03-0.18) |
| Portugal | 176 (131-240) | 1.8 (1.3-2.46) | 105 (82-141) | 0.92 (0.71-1.3) | -1.83 (-2.11--1.56) |
| Puerto Rico | 66153 (910-262716) | 1827.94 (25.14-7259.36) | 57306 (233-230391) | 1674.07 (6.67-6730.5) | 0.71 (-0.4-1.83) |
| Qatar | 4 (4-6) | 1.08 (0.91-1.27) | 24 (19-30) | 0.74 (0.61-0.89) | -1.82 (-2.1--1.54) |
| Republic of Korea | 1904 (907-3486) | 4.19 (2.01-7.64) | 674 (315-1385) | 1.12 (0.53-2.37) | -5.94 (-7.99--3.84) |
| Republic of Moldova | 1213 (961-1504) | 26.65 (21.32-32.48) | 1149 (906-1429) | 25.93 (20.96-31.86) | -0.06 (-0.12-0.01) |
| Romania | 1597 (960-3186) | 7.02 (4-14.61) | 1106 (933-1307) | 5.07 (4.29-5.99) | -1.41 (-1.67--1.15) |
| Russian Federation | 3923 (3105-4811) | 2.35 (1.87-2.87) | 3350 (2652-4095) | 1.91 (1.51-2.33) | -0.52 (-0.6--0.43) |
| Rwanda | 1548059 (725825-2625770) | 15349.85 (7818.31-24633.1) | 562877 (395223-761000) | 3723.18 (2697.54-5113.95) | -5.38 (-6.42--4.33) |
| Saint Kitts and Nevis | 43 (1-244) | 102.75 (1.55-578.53) | 59 (3-394) | 101.47 (5.71-671.86) | 4.98 (1.35-8.73) |
| Saint Lucia | 162 (5-513) | 117.26 (4.16-370.83) | 440 (113-1064) | 250.51 (63.83-607.89) | 4.21 (2.18-6.28) |
| Saint Vincent and the Grenadines | 318 (10-993) | 284.01 (8.88-888.1) | 394 (20-1258) | 346.97 (17.24-1109.39) | 0.32 (-1.66-2.35) |
| Samoa | 299 (9-986) | 175.73 (5.95-576.78) | 1187 (367-3460) | 555.15 (171.93-1611.64) | 2.91 (1.68-4.15) |
| San Marino | 0 (0-0) | 0.04 (0.02-0.05) | 0 (0-0) | 0.02 (0.01-0.03) | -3.18 (-3.73--2.62) |
| Sao Tome and Principe | 24445 (2305-102697) | 16223.68 (2118.78-59166.01) | 6399 (4209-17448) | 3006.96 (2001.37-8065.49) | -7.1 (-9.34--4.79) |
| Saudi Arabia | 34949 (22305-56121) | 230.25 (150.82-356.16) | 7370 (4261-12361) | 20.8 (11.82-35.12) | -8.05 (-8.75--7.34) |
| Senegal | 724443 (301131-1525830) | 8630.57 (4129.05-15983.87) | 976326 (832731-1129859) | 6044.64 (5227.78-6899.44) | -2.76 (-3.73--1.77) |
| Serbia | 547 (361-940) | 5.77 (3.64-10.42) | 377 (306-453) | 3.58 (2.93-4.26) | -1.58 (-1.77--1.38) |
| Seychelles | 5205 (25-25697) | 7057.05 (33.55-34838.76) | 12844 (2397-54246) | 12418.34 (2318.4-52444.79) | 1.72 (1.46-1.98) |
| Sierra Leone | 1939071 (1111647-2501669) | 33278.79 (20040.47-43482.25) | 2957410 (1627663-4203635) | 25752.58 (14515.46-36840.36) | -0.63 (-0.82--0.43) |
| Singapore | 218943 (32165-572750) | 7258.16 (1064.86-18986.47) | 485144 (107236-1122962) | 8714.82 (1927.61-20177.52) | 1.33 (0.23-2.44) |
| Slovakia | 77 (61-93) | 1.4 (1.1-1.71) | 76 (60-93) | 1.24 (0.98-1.51) | -0.42 (-0.49--0.35) |
| Slovenia | 82 (58-126) | 4.16 (2.85-6.65) | 59 (47-73) | 2.53 (2.02-3.18) | -1.53 (-1.7--1.35) |
| Solomon Islands | 187560 (42241-511265) | 42199.49 (12182.51-103688.81) | 211871 (186274-238378) | 27628.3 (24630.74-30867.97) | -3.83 (-4.95--2.68) |
| Somalia | 1370094 (843301-2269664) | 12517.13 (7975-19649.24) | 1455692 (865306-2055130) | 5251.53 (3235-7233.06) | -4.84 (-5.87--3.8) |
| South Africa | 80478 (6534-334250) | 218.18 (18.34-876.82) | 25769 (19928-32500) | 44.55 (34.42-56.27) | -5.82 (-8--3.59) |
| South Sudan | 2401468 (1770397-3046012) | 31837.49 (24501.1-39516.23) | 2576537 (1507322-3760344) | 19844.41 (12019.09-28779.43) | -1.65 (-1.9--1.39) |
| Spain | 1359 (1069-1819) | 3.51 (2.72-4.72) | 1154 (921-1480) | 2.28 (1.81-2.99) | -0.87 (-1.22--0.51) |
| Sri Lanka | 917193 (776513-1095381) | 5351.01 (4545.34-6378.99) | 474505 (130251-1564912) | 2142.93 (587.46-7059.71) | -3.96 (-6.09--1.78) |
| Sudan | 2409782 (1699753-3151997) | 9554.31 (7199.83-12461.14) | 3641010 (1782876-6433488) | 7122.6 (3514.64-12565.75) | -2.92 (-4--1.83) |
| Suriname | 24993 (2725-121326) | 6196.24 (730.96-28087.75) | 3457 (740-7164) | 592.65 (127.17-1233.64) | -7.99 (-10.09--5.83) |
| Sweden | 6 (5-9) | 0.06 (0.04-0.09) | 9 (6-13) | 0.08 (0.05-0.12) | 0.58 (0-1.16) |
| Switzerland | 7 (5-9) | 0.08 (0.06-0.11) | 10 (7-13) | 0.08 (0.06-0.11) | -0.59 (-1.96-0.79) |
| Syrian Arab Republic | 46658 (19975-99871) | 368.56 (154.71-796.61) | 272558 (206827-351153) | 1978 (1492.17-2543.48) | 5.91 (4.9-6.94) |
| Taiwan (Province of China) | 33287 (4804-111532) | 163.3 (23.8-546.72) | 35165 (2712-126129) | 149.89 (11.65-537.76) | -0.07 (-0.33-0.19) |
| Tajikistan | 42366 (8398-151620) | 855.73 (171.66-2995.49) | 1242 (983-1562) | 12.66 (10.12-15.76) | -17.55 (-21.77--13.09) |
| Thailand | 1067635 (860509-1299934) | 1878.56 (1515.15-2286.2) | 423333 (121584-924795) | 639.41 (183.45-1390.15) | -0.78 (-1.99-0.45) |
| Timor-Leste | 76549 (6620-379845) | 8745.66 (921.48-38764.19) | 3153 (854-8036) | 223.69 (60.97-569.25) | -11.97 (-15.68--8.09) |
| Togo | 1779906 (1257256-2298846) | 33034.47 (24429.77-42090.58) | 2030496 (1105743-3090619) | 19760.02 (10929.99-28934.95) | -1.65 (-2.03--1.27) |
| Tokelau | 0 (0-1) | 13.99 (3.52-48.32) | 1 (0-2) | 40.83 (1.41-169.47) | 4.96 (2.99-6.97) |
| Tonga | 985 (103-3898) | 985.88 (104.09-3900.49) | 15325 (146-54322) | 14364.12 (137.3-50917.37) | 16.96 (12.7-21.38) |
| Trinidad and Tobago | 6711 (507-20021) | 554.57 (41.95-1654.45) | 8129 (418-24354) | 587.38 (30.02-1760.2) | 0.63 (-0.19-1.46) |
| Tunisia | 8536 (3731-17881) | 102.1 (44.92-212.56) | 24336 (16614-36608) | 207.35 (139.29-313.72) | 2.21 (0.34-4.11) |
| Turkey | 28358 (17838-44143) | 49.43 (31.52-77.12) | 13313 (6130-25322) | 16.46 (7.43-31.56) | -4.78 (-5.51--4.05) |
| Turkmenistan | 9181 (6590-12657) | 218.89 (157.13-301.18) | 1609 (975-2592) | 31.59 (19.02-50.99) | -6.1 (-7.22--4.98) |
| Tuvalu | 7 (1-28) | 78.52 (17.51-304.2) | 57 (1-243) | 459.17 (6.36-1971.89) | 7.46 (5.29-9.68) |
| Uganda | 9357603 (7543461-11563436) | 39046.27 (32412.4-47872.77) | 10296634 (7720101-13034131) | 17204.21 (12975.68-21440.81) | -2.55 (-2.78--2.32) |
| Ukraine | 4051 (3199-4951) | 6.78 (5.4-8.3) | 3357 (2651-4152) | 6.22 (4.92-7.61) | -0.3 (-0.37--0.22) |
| United Arab Emirates | 18 (15-22) | 1.19 (1.02-1.37) | 77 (60-95) | 0.65 (0.55-0.76) | -1.74 (-1.9--1.58) |
| United Kingdom | 112 (81-152) | 0.18 (0.13-0.24) | 166 (120-226) | 0.23 (0.17-0.32) | 0.52 (0.35-0.68) |
| United Republic of Tanzania | 9625202 (7409395-11990241) | 26274.61 (20162.45-33000.8) | 7363116 (4701875-10440647) | 10179.62 (6584.23-13867.17) | -4.3 (-4.8--3.8) |
| United States of America | 1034 (701-1774) | 0.39 (0.26-0.69) | 2314 (863-8123) | 0.65 (0.24-2.32) | 3.06 (2.29-3.83) |
| United States Virgin Islands | 230 (4-1346) | 219.32 (3.49-1282.79) | 164 (4-919) | 190.95 (4.52-1071.63) | 1.16 (-0.85-3.21) |
| Uruguay | 1139 (992-1316) | 37.6 (32.76-43.57) | 226 (180-273) | 6.18 (5.01-7.63) | -6.47 (-7.88--5.03) |
| Uzbekistan | 4731 (2273-9204) | 22.92 (11.36-43.99) | 6044 (4559-7981) | 17.91 (13.43-23.65) | -0.04 (-0.82-0.74) |
| Vanuatu | 70857 (63917-79215) | 37163.36 (33961.26-40915.51) | 3133 (1395-7901) | 1015.03 (460.13-2540.19) | -9.06 (-10.99--7.09) |
| Venezuela (Bolivarian Republic of) | 308604 (161144-572193) | 1683.57 (904.41-3078.69) | 792563 (409930-1327510) | 2943.65 (1508.46-4956.88) | 3.95 (3.03-4.89) |
| Viet Nam | 565105 (152398-1217982) | 839.32 (230.78-1788.67) | 1105499 (411299-2672493) | 1119.82 (416.72-2702.76) | 0.38 (0.07-0.68) |
| Yemen | 1845992 (511574-4844754) | 11459.79 (4018.46-25859.31) | 2243444 (1745351-2821213) | 6526.31 (5207.03-8011.76) | -2.67 (-4.35--0.95) |
| Zambia | 3043991 (2312070-3931229) | 26316.18 (20257.34-33433.81) | 3498417 (2070129-4941250) | 13885.46 (8401.68-19373.67) | -2.82 (-3.39--2.24) |
| Zimbabwe | 712772 (573767-870858) | 6646.62 (5516.28-7885.04) | 586204 (439343-772308) | 3827.1 (2922.32-4945.86) | 0.35 (-1.04-1.76) |

**Table S2.** The number of prevalence cases and the age-standardized prevalence rate of neglected tropical diseases and malaria in 1990 and 2021, and its trends from 1990 to 2021 globally.

|  | Number of prevalence cases (95% UI) in 1990 | The age-standardized prevalence rate/100000 (95% UI) in 1990 | Number of prevalence cases (95% UI) in 2021 | The age-standardized prevalence rate/100000 (95% UI) in 2021 | EAPC (95% CI) |
| --- | --- | --- | --- | --- | --- |
| Global | 1915551162 (1816268530-2023083395) | 34451.84 (32668.32-36407.73) | 1111497287 (1049604638-1189217544) | 14454.15 (13659.74-15452.2) | -2.98 (-3.05--2.91) |
| Sex |  |  |  |  |  |
| Female | 934259567 (885432686-987256086) | 33955.1 (32174.29-35907.12) | 555133483 (525006124-592326546) | 14650.04 (13859.41-15635.52) | -2.89 (-2.96--2.83) |
| Male | 981291594 (929884533-1036347947) | 35019.5 (33205.08-37001.16) | 556363804 (523730912-596555694) | 14276.26 (13443.47-15298.13) | -3.06 (-3.14--2.99) |
| Age |  |  |  |  |  |
| <5 years | 210613401 (200352009-220870415) | 33973.3 (32318.08-35627.83) | 104956349 (100383550-110612643) | 15946.65 (15251.88-16806.05) | -2.75 (-2.9--2.6) |
| 5-9 years | 315108078 (297848374-331986934) | 54000.26 (51042.45-56892.8) | 152965576 (143898408-164702776) | 22264 (20944.28-23972.33) | -2.98 (-3.11--2.85) |
| 10-14 years | 273055559 (257291888-288379084) | 50973.38 (48030.66-53833.94) | 144220872 (133863148-159234664) | 21634.13 (20080.4-23886.3) | -2.83 (-2.91--2.74) |
| 15-19 years | 226391598 (213599000-239774629) | 43585.17 (41122.33-46161.69) | 124126946 (113965330-136959075) | 19892.78 (18264.26-21949.28) | -2.64 (-2.7--2.58) |
| 20-24 years | 183878545 (172534226-196002416) | 37367.01 (35061.67-39830.77) | 102960077 (93491607-113853622) | 17241.68 (15656.09-19065.91) | -2.68 (-2.76--2.6) |
| 25-29 years | 152007753 (142376586-162468402) | 34342.75 (32166.8-36706.1) | 91055983 (83027343-100425755) | 15476.68 (14112.06-17069.25) | -2.82 (-2.91--2.74) |
| 30-34 years | 120406147 (113217104-128457031) | 31240.04 (29374.8-33328.89) | 80384770 (73063680-88247649) | 13298.16 (12087.03-14598.93) | -2.91 (-2.98--2.85) |
| 35-39 years | 100129624 (93879897-107026536) | 28426.17 (26651.91-30384.16) | 65054669 (59877986-71506356) | 11598.97 (10675.99-12749.27) | -3.02 (-3.08--2.95) |
| 40-44 years | 74658426 (70303274-79397487) | 26060.46 (24540.24-27714.69) | 52522499 (48115413-58697405) | 10499.23 (9618.26-11733.6) | -3.16 (-3.27--3.06) |
| 45-49 years | 60990869 (57621026-64798974) | 26267.03 (24815.74-27907.08) | 45784524 (42541411-49766448) | 9669.29 (8984.37-10510.23) | -3.41 (-3.5--3.32) |
| 50-54 years | 53464564 (50493165-57061657) | 25151.34 (23753.5-26843.52) | 40234356 (37446500-43384002) | 9042.99 (8416.4-9750.9) | -3.58 (-3.71--3.45) |
| 55-59 years | 44673496 (42190021-47639079) | 24121.76 (22780.79-25723.05) | 33017362 (30706895-35789338) | 8343.45 (7759.6-9043.92) | -3.72 (-3.86--3.58) |
| 60-64 years | 35638400 (33668685-38125143) | 22189.51 (20963.11-23737.83) | 23953342 (22244969-26220735) | 7484.3 (6950.51-8192.75) | -3.88 (-4.02--3.73) |
| 65-69 years | 27544941 (25947785-29513766) | 22283.85 (20991.75-23876.62) | 20253426 (18941409-21917898) | 7342.4 (6866.76-7945.81) | -3.83 (-3.93--3.73) |
| 70-74 years | 18238628 (17165503-19473645) | 21543.09 (20275.54-23001.87) | 13702019 (12822788-14810067) | 6656.66 (6229.52-7194.97) | -3.81 (-3.88--3.74) |
| 75-79 years | 10879829 (10229401-11576381) | 17674.82 (16618.17-18806.41) | 8429927 (7837682-9082808) | 6391.91 (5942.84-6886.95) | -3.7 (-3.84--3.55) |
| 80-84 years | 5365193 (5048818-5691829) | 15166.23 (14271.9-16089.56) | 4741216 (4435809-5108603) | 5413.39 (5064.69-5832.87) | -3.6 (-3.76--3.44) |
| 85-89 years | 1927194 (1822222-2045494) | 12753.52 (12058.85-13536.39) | 2134416 (1998533-2294572) | 4668.27 (4371.07-5018.55) | -3.6 (-3.77--3.43) |
| 90-94 years | 481141 (455119-509518) | 11228.01 (10620.75-11890.22) | 779729 (730012-837625) | 4358.62 (4080.7-4682.25) | -3.38 (-3.52--3.24) |
| 95+ years | 97776 (92156-104933) | 9603.9 (9051.89-10306.9) | 219229 (201171-240797) | 4022.32 (3691.01-4418.04) | -2.95 (-3.08--2.81) |
| SDI region | |  |  |  |  |
| High-middle SDI | 206131329 (170260831-245066982) | 19275.42 (15942.05-22903.08) | 72482610 (62953513-84740046) | 5865 (5034.09-6955.43) | -3.96 (-4.44--3.48) |
| High SDI | 26664354 (23188651-30814475) | 3205.24 (2787.43-3703.68) | 17500461 (15466902-19537758) | 1696.42 (1482.81-1920.46) | -1.63 (-2.03--1.23) |
| Low-middle SDI | 628587660 (593309760-662149731) | 52084.14 (49113.98-54868.34) | 362873536 (328711487-404161134) | 18359.65 (16652.27-20418) | -3.68 (-3.91--3.46) |
| Low SDI | 360426308 (349862427-370440984) | 70314.54 (68139.34-72426.81) | 377357975 (355206316-400137718) | 31872.95 (29927.13-33898.59) | -2.69 (-2.96--2.43) |
| Middle SDI | 692470064 (635781266-750702630) | 38717.14 (35523.24-41932.66) | 280531305 (258611547-307326582) | 11709.22 (10785.39-12827.83) | -4.11 (-4.29--3.92) |
| GBD region | |  |  |  |  |
| Advanced Health System | 29827487 (27036580-33007691) | 2393.61 (2163.45-2658.2) | 23902484 (21763867-26454723) | 1699.14 (1522.6-1900.99) | -1.08 (-1.18--0.97) |
| Africa | 396952786 (386793415-407121719) | 61590.78 (59874.82-63402.36) | 481000990 (448861792-515483550) | 32964.25 (30755.43-35388.82) | -2.18 (-2.4--1.96) |
| African Region | 356816172 (347709595-366101940) | 68794.09 (66878.89-70783.34) | 442714874 (413619553-475292146) | 36578.41 (34046.88-39333.19) | -2.16 (-2.37--1.94) |
| America | 123485171 (115102321-133501798) | 16666.46 (15545.34-18000.04) | 118504660 (107260777-131675252) | 12215.81 (11045.85-13590.25) | -0.83 (-0.99--0.68) |
| Andean Latin America | 11709027 (10554053-13005197) | 29854.13 (27040.6-33071.05) | 22923103 (20311491-25505385) | 34400.62 (30459.26-38263.97) | 0.75 (0.5-0.99) |
| Asia | 1379720011 (1278370063-1488616285) | 41785.36 (38735.01-45125.58) | 499774087 (447832321-565832243) | 11098.17 (9941.97-12595.12) | -4.51 (-4.6--4.42) |
| Australasia | 80081 (64492-116340) | 431.42 (335.62-662.99) | 83646 (70728-113853) | 310.59 (246.65-469.82) | -1.02 (-1.16--0.89) |
| Basic Health System | 856165203 (770396340-951777793) | 35813.01 (32241.33-39734.47) | 324051587 (295981016-356720686) | 10650.51 (9708.05-11714.09) | -4.08 (-4.4--3.76) |
| Caribbean | 13258156 (11745886-15515322) | 36682.26 (32299.85-43336.83) | 7132584 (6338801-8062364) | 15290.06 (13616.74-17233.09) | -3.66 (-4.17--3.14) |
| Central Africa | 54230127 (52758017-55537464) | 80311.03 (77960.65-82542.98) | 85557004 (79609671-91515778) | 49505.33 (46108.65-52777.42) | -1.47 (-1.69--1.24) |
| Central Asia | 7734337 (6829013-8860647) | 10434.08 (9225.34-11962.15) | 5186268 (4556962-6057694) | 5394.67 (4741.08-6303.27) | -1.91 (-2.14--1.67) |
| Central Europe | 1644444 (1555913-1745185) | 1393.01 (1321.03-1478.49) | 994635 (932999-1067606) | 964.67 (908.24-1042.86) | -1.25 (-1.3--1.19) |
| Central Latin America | 54109953 (49346933-59221377) | 30415.11 (27774.26-33240.01) | 43924827 (38304689-51423705) | 17447.05 (15224.43-20416.36) | -1.7 (-1.82--1.58) |
| Central Sub-Saharan Africa | 45298735 (43880414-46722627) | 81832.95 (79062.69-84687.15) | 71425883 (65652268-77688285) | 50673.43 (46718.33-54969.05) | -1.45 (-1.66--1.24) |
| Commonwealth High Income | 713919 (635167-802706) | 716.14 (639.85-804.01) | 579897 (514421-660225) | 462.08 (403.32-527.87) | -1.57 (-1.93--1.2) |
| Commonwealth Low Income | 136051362 (129013060-142702240) | 62388.16 (59199.71-65643.42) | 121081076 (110645381-132614420) | 29175.28 (26643.34-32024.87) | -2.6 (-2.92--2.29) |
| Commonwealth Middle Income | 680941951 (622900067-739022112) | 55852.46 (51192.83-60475.28) | 383854910 (334285744-449603353) | 17774.66 (15476.15-20793.68) | -4.05 (-4.31--3.79) |
| East Asia | 464515592 (376195555-562085142) | 37386.1 (30375.05-45101.09) | 113170033 (92384113-141056672) | 7914.95 (6279.03-10099.51) | -5.17 (-5.84--4.5) |
| East Asia & Pacific - WB | 731570781 (644091574-829007706) | 38107.75 (33652.83-43121.56) | 193459770 (170220325-222888398) | 8515.83 (7440.72-9880.99) | -5.14 (-5.57--4.71) |
| Eastern Africa | 115322140 (110673790-120086403) | 64276.72 (61405.35-67460.59) | 128301123 (117592412-140214960) | 31900.31 (29095.46-34824.43) | -2.45 (-2.69--2.2) |
| Eastern Europe | 4167938 (3880764-4476082) | 1802.52 (1684.91-1921.13) | 3482902 (3190453-3801118) | 1542.07 (1430.2-1663.58) | -0.5 (-0.56--0.44) |
| Eastern Mediterranean Region | 126908058 (117247528-135406331) | 32146.07 (29607.6-34466.37) | 105748181 (93463140-120610993) | 13389.11 (11858.82-15265.26) | -3.34 (-3.6--3.08) |
| Eastern Sub-Saharan Africa | 137122834 (132017098-142083484) | 70730.77 (67938.06-73625.92) | 151053666 (139481832-163809996) | 34023.35 (31350.35-36978.03) | -2.56 (-2.82--2.3) |
| Europe | 14038889 (12525232-16257941) | 1869.8 (1663.35-2172.52) | 11414703 (9994360-13461115) | 1513.68 (1294.82-1814.84) | -1.1 (-1.27--0.93) |
| Europe & Central Asia - WB | 19872952 (18067144-22230097) | 2502.42 (2270.01-2810.87) | 15702930 (13985432-17768111) | 1960.33 (1732.2-2234.11) | -1.06 (-1.15--0.96) |
| European Region | 19929811 (18124873-22286987) | 2486.01 (2255.44-2791.47) | 15763588 (14041858-17829596) | 1937.61 (1713.28-2207.37) | -1.07 (-1.17--0.98) |
| High-income Asia Pacific | 7867836 (6120067-10155477) | 4772.39 (3673.27-6198.05) | 5117809 (4186049-6399104) | 2999.1 (2336.32-3937.18) | -0.58 (-1.12--0.04) |
| High-income North America | 1202383 (1100432-1320414) | 412.21 (378.69-451.53) | 1646428 (1485648-1848435) | 407.81 (370.05-452.25) | 0.17 (0.1-0.25) |
| Latin America & Caribbean - WB | 122339405 (113938116-132363326) | 26363.27 (24599.06-28456.91) | 116892675 (105679566-130144113) | 18025.37 (16296.22-20069.9) | -1.05 (-1.2--0.9) |
| Limited Health System | 925694580 (865151693-982953459) | 56872.88 (53214.62-60306.92) | 607579943 (550387712-677282400) | 20333.97 (18468.68-22644.47) | -3.63 (-3.88--3.37) |
| Middle East & North Africa - WB | 46436514 (43468909-50129977) | 17765.08 (16639.3-19312.69) | 45301979 (40560669-50553384) | 9388.75 (8422.19-10473.14) | -2.71 (-3.05--2.37) |
| Minimal Health System | 102592443 (100345464-104956205) | 75010.61 (73225.75-76868.18) | 155211873 (146727816-163038418) | 43917.7 (41572.28-46099.54) | -1.67 (-1.89--1.45) |
| North Africa and Middle East | 60799985 (57072086-65053024) | 17427.01 (16345.49-18780.39) | 64408540 (58727151-70734095) | 10127.62 (9245.79-11103.1) | -2.38 (-2.7--2.05) |
| North America | 1206598 (1103954-1323994) | 413.78 (380.24-453.42) | 1648103 (1487309-1850345) | 408.3 (370.44-452.74) | 0.16 (0.09-0.24) |
| Northern Africa | 30709318 (28670129-33729356) | 25342.68 (23681.2-28223.96) | 21896745 (18683326-26122052) | 10210.34 (8736.8-12159.99) | -3.52 (-3.98--3.06) |
| Oceania | 4341026 (3788810-4920698) | 66252.03 (56711.14-76121.98) | 4421448 (3671889-5469166) | 30980.05 (25372.61-39083.44) | -2.79 (-2.92--2.65) |
| Region of the Americas | 123485171 (115102321-133501798) | 16666.46 (15545.34-18000.04) | 118504660 (107260777-131675252) | 12215.81 (11045.85-13590.25) | -0.83 (-0.99--0.68) |
| South-East Asia Region | 722702932 (662881618-782829822) | 53491.55 (49141.04-57807.37) | 277034475 (229814208-339655342) | 13458.59 (11164.91-16509.62) | -4.76 (-5.02--4.49) |
| South Asia | 614832992 (555223947-671443150) | 54006.74 (48878-58904) | 269596038 (221193898-334220995) | 14454.51 (11876.47-17902.31) | -4.55 (-4.88--4.21) |
| South Asia - WB | 626252184 (566992570-683281165) | 53636.81 (48666.38-58403.07) | 278060159 (230157639-342849460) | 14463.25 (11983.16-17817.88) | -4.53 (-4.85--4.2) |
| Southeast Asia | 263607727 (242165268-286516805) | 54528.61 (49732.67-60111.16) | 72779599 (63742030-84308146) | 10535.33 (9224.65-12197.53) | -5.92 (-6.17--5.67) |
| Southern Africa | 60557023 (57784493-63516351) | 61733.35 (58703.77-65143.92) | 52703959 (47983734-58230324) | 26846.12 (24380.6-29725.08) | -2.91 (-3.06--2.75) |
| Southern Latin America | 8870297 (7243952-10665958) | 17694.61 (14479.68-21224.44) | 5309534 (4242332-6608140) | 8085.16 (6438.94-10156.18) | -1.74 (-2.27--1.21) |
| Southern Sub-Saharan Africa | 28765117 (26477130-30766125) | 51207.48 (47010.97-54918.91) | 15395925 (13295208-17427959) | 18580.5 (16133-20968.9) | -3.53 (-3.64--3.42) |
| Sub-Saharan Africa - WB | 366572422 (356980511-375769319) | 70353.34 (68322.46-72462.49) | 459660370 (429275919-492609528) | 37367.5 (34901.89-40256.4) | -2.16 (-2.37--1.95) |
| Tropical Latin America | 34845849 (28598813-42961774) | 21588.79 (17901.48-26468.14) | 37846163 (30105676-47450656) | 17092.56 (13504.92-21498.66) | -0.27 (-0.52--0.01) |
| Western Africa | 136134177 (131356913-140971284) | 77831.69 (74715.33-81250.88) | 192542158 (175063463-209590776) | 42598.4 (38700.83-46526.42) | -2.1 (-2.32--1.87) |
| Western Europe | 1600969 (1464293-1783048) | 466.92 (425.65-518.75) | 1280009 (1200592-1406148) | 328.37 (304.49-362.59) | -1.1 (-1.21--0.99) |
| Western Pacific Region | 560412446 (472993389-656505121) | 35478.06 (29966.32-41535.17) | 149699519 (127228265-179103077) | 8129.89 (6809.69-9777.57) | -4.98 (-5.5--4.46) |
| Western Sub-Saharan Africa | 149175883 (144276160-154073165) | 76935.23 (73976.45-80062.23) | 214318246 (196750430-231735983) | 42092.76 (38557.48-45646.19) | -2.08 (-2.3--1.85) |
| World Bank High Income | 21208691 (18998961-23881083) | 2210.31 (1975.72-2494.71) | 16129128 (14703465-17740475) | 1421.24 (1284.56-1571.19) | -1.05 (-1.31--0.79) |
| World Bank Low Income | 196629328 (191529148-202064456) | 61959.43 (60131.09-63901.38) | 249841531 (237472804-263215368) | 34264.27 (32541.33-36203.25) | -1.98 (-2.18--1.78) |
| World Bank Lower Middle Income | 1058782206 (998831132-1118030088) | 50937.26 (47984.45-53802.88) | 593676634 (538994592-660884003) | 16929.69 (15383.26-18836.54) | -3.89 (-4.11--3.67) |
| World Bank Upper Middle Income | 637656534 (552062395-733723275) | 30964.31 (26852.52-35673.1) | 251097501 (224822320-282410268) | 10441.63 (9326.23-11775.78) | -3.55 (-3.94--3.16) |
| Country |  |  |  |  |  |
| Afghanistan | 3374164 (2556594-4184708) | 31704.17 (24158.34-39257.46) | 6884802 (5246071-8765627) | 20225.73 (15604.28-25486.62) | -1.7 (-2.01--1.38) |
| Albania | 63622 (54138-78270) | 1789.1 (1539.61-2174.83) | 24100 (21725-27703) | 1038.52 (923.88-1259.79) | -2.05 (-2.24--1.86) |
| Algeria | 2198396 (1626721-3090573) | 7928.68 (5929.34-11072.57) | 3307747 (2422270-4354662) | 7422.38 (5438.56-9769.14) | -0.91 (-1.23--0.58) |
| American Samoa | 4581 (4019-5228) | 10772.54 (9438.94-12273.52) | 2785 (1583-5392) | 5608.13 (3246.06-10767.32) | -5.42 (-7.19--3.62) |
| Andorra | 163 (142-198) | 351.16 (304.85-429.06) | 201 (178-228) | 275.89 (239.15-320.32) | -0.67 (-0.81--0.53) |
| Angola | 7589577 (6924578-8331804) | 71758.38 (65112.73-79452.37) | 12430808 (10253904-14961534) | 35756.02 (29750.86-42766.96) | -2.52 (-2.69--2.35) |
| Antigua and Barbuda | 12656 (9327-16240) | 20256.2 (15039.76-25854.26) | 11699 (7186-17035) | 13250.82 (8518.26-18959.75) | -1.64 (-2.2--1.06) |
| Argentina | 6657229 (5151598-8354465) | 19940.32 (15466.98-24980.96) | 3995212 (2975407-5310391) | 9005.77 (6659.54-12016.15) | -1.77 (-2.29--1.24) |
| Armenia | 271600 (201272-379483) | 7766.77 (5758.39-10866.76) | 86650 (66173-115136) | 3141.01 (2397.64-4157.19) | -2.63 (-3.1--2.16) |
| Australia | 64546 (49617-99155) | 417.21 (303.89-686.08) | 67899 (55931-98799) | 297.91 (229.6-496.44) | -1.05 (-1.2--0.9) |
| Austria | 33263 (28250-40717) | 466.81 (392.23-598.73) | 29049 (25687-33603) | 322.76 (283.73-373.42) | -1.22 (-1.31--1.13) |
| Azerbaijan | 781315 (574376-1071856) | 10107.28 (7411.43-13894) | 437905 (327296-609881) | 4355.2 (3266-6057.71) | -2.59 (-2.81--2.37) |
| Bahamas | 35048 (25491-47846) | 12707.15 (9277.76-17284.64) | 27956 (20626-38200) | 7513.56 (5533.39-10201.72) | -2.19 (-3.12--1.25) |
| Bahrain | 30476 (22464-43199) | 5635.94 (4108.65-8025.73) | 91831 (66079-126250) | 6184.36 (4470.79-8501.66) | -0.7 (-1.09--0.3) |
| Bangladesh | 59793528 (53460304-65818743) | 50847.41 (45485.85-56004.3) | 41614978 (33453763-50530718) | 24636.97 (19820.68-29821.81) | -2.62 (-2.94--2.3) |
| Barbados | 32391 (24146-43345) | 12865.86 (9607.33-17175.67) | 19422 (13885-26364) | 7583.59 (5390.95-10303.22) | -2 (-2.89--1.1) |
| Belarus | 117175 (106905-128665) | 1161.78 (1060.06-1276.39) | 73090 (66651-80885) | 828.4 (751.09-939.68) | -1.24 (-1.35--1.12) |
| Belgium | 33290 (29526-38393) | 390.43 (338.05-466.62) | 27644 (24675-31421) | 282.61 (245.39-329.39) | -1.08 (-1.15--1.02) |
| Belize | 43863 (33985-56347) | 21037.02 (16267-27069.98) | 30922 (23727-39959) | 6933.48 (5352.78-8890.04) | -4.04 (-5.15--2.9) |
| Benin | 3696510 (3455551-3903265) | 76206.61 (71715.89-80616.8) | 6276677 (5322289-7325028) | 44154.44 (37825.02-51651.97) | -1.36 (-1.66--1.06) |
| Bermuda | 4518 (3423-5895) | 8058.93 (6125.58-10503.57) | 1870 (1368-2514) | 3687.77 (2654.78-5027.72) | -2.91 (-3.79--2.03) |
| Bhutan | 307703 (233152-470256) | 44937.64 (33696.16-69764.54) | 52654 (39803-71960) | 7143.27 (5449.26-9717.84) | -6.03 (-6.35--5.71) |
| Bolivia (Plurinational State of) | 1990630 (1794816-2223140) | 32278.82 (29430.05-35597.93) | 2523066 (2102333-3044410) | 21326.78 (17835.09-25702.86) | -1.2 (-1.51--0.9) |
| Bosnia and Herzegovina | 61956 (55408-69175) | 1416.85 (1272.56-1590.5) | 31026 (27906-35601) | 1053.44 (941.68-1214.37) | -1.21 (-1.35--1.07) |
| Botswana | 664788 (567537-757483) | 47047.43 (40550.74-53703.04) | 601478 (485914-731721) | 23907.5 (19408.69-28833.8) | -1.85 (-2.29--1.4) |
| Brazil | 33714296 (27494575-41769373) | 21468.94 (17751.54-26440.56) | 36849100 (29253048-46388690) | 17209.4 (13509.06-21765.34) | -0.22 (-0.48-0.03) |
| Brunei Darussalam | 5780 (2241-31567) | 2374.15 (858.91-13647.11) | 6543 (2989-26541) | 1418.49 (675.12-5616.58) | -2.82 (-3.6--2.04) |
| Bulgaria | 97569 (87907-111052) | 1273.45 (1138.55-1469.21) | 58664 (53096-65815) | 1057.88 (943.12-1222.82) | -0.64 (-0.72--0.57) |
| Burkina Faso | 8382633 (8052155-8771753) | 86682.87 (82302.78-92442.32) | 7595831 (5277070-10137102) | 30833.77 (21429.45-41250.8) | -3.68 (-4.28--3.08) |
| Burundi | 3964716 (3369775-4478630) | 68558.53 (58810.14-77224.57) | 5532351 (4454324-6845263) | 39063.6 (31439.4-48371.4) | -2.2 (-2.53--1.86) |
| Cabo Verde | 99022 (78060-120608) | 25103.16 (19795.87-30734.55) | 49952 (37799-65713) | 8945.86 (6795.46-11758.85) | -3.15 (-3.46--2.84) |
| Cambodia | 5568168 (4893242-6364631) | 50507.18 (43875.69-60622.11) | 1441386 (1114744-1853927) | 8330.15 (6443.45-10693.15) | -6.47 (-6.85--6.09) |
| Cameroon | 8557573 (8250273-8848974) | 80712.73 (77784.22-83638.09) | 12729645 (10807022-14835917) | 38389.23 (32648.45-44810.4) | -2.56 (-2.84--2.28) |
| Canada | 80139 (47992-133754) | 285.63 (181.4-449.7) | 83165 (59243-140463) | 233.16 (164.36-414.82) | -0.59 (-0.7--0.47) |
| Central African Republic | 2349680 (2154493-2502915) | 86423.26 (79585.11-93218.7) | 3176878 (2656453-3982510) | 56730.22 (47807.89-70690.99) | -1.35 (-1.5--1.2) |
| Chad | 3937865 (3541511-4320789) | 63390.5 (56938.15-70061.27) | 8252229 (6944886-9736633) | 42921.27 (36034.55-50749.85) | -0.74 (-0.98--0.49) |
| Chile | 2145599 (1605361-2732928) | 15732.69 (11869.21-19972.44) | 1274472 (935547-1702844) | 6980.59 (5020.99-9486.85) | -1.87 (-2.4--1.33) |
| China | 454654226 (366900468-551194805) | 37873.76 (30591.08-45815.59) | 110109106 (89167484-137839619) | 7949.82 (6266.79-10207.15) | -5.2 (-5.86--4.53) |
| Colombia | 10201480 (7921576-12640493) | 29152.03 (22760.97-36193.8) | 7649683 (5573613-10400945) | 16078.64 (11707.59-21852.9) | -1.98 (-2.07--1.88) |
| Comoros | 194968 (133820-326807) | 41161.87 (27268.8-73207.84) | 110655 (73962-206036) | 14578.53 (9565.31-28135.28) | -3.24 (-3.52--2.95) |
| Congo | 1643176 (1481444-1802966) | 65752.73 (59230.82-72970.21) | 1896322 (1529013-2408036) | 33783.39 (27298.52-42936.57) | -1.95 (-2.09--1.81) |
| Cook Islands | 2098 (1588-2750) | 10357.89 (7868.92-13537) | 852 (638-1141) | 5211.03 (3904.64-6961.36) | -2.04 (-2.27--1.82) |
| Costa Rica | 820643 (644139-1028781) | 24956.64 (19718.08-31237.43) | 712779 (509480-940210) | 15587.92 (11095.82-20588.97) | -1.51 (-1.6--1.41) |
| Croatia | 54496 (49032-60020) | 1166.36 (1053.58-1301.3) | 33117 (29813-37078) | 830.97 (755.17-918.09) | -1.18 (-1.24--1.12) |
| Cuba | 5742341 (5000111-6440938) | 53148.63 (46342.18-59633.52) | 2134314 (1666175-2688591) | 21422.15 (16748.76-26886.94) | -3.65 (-4.14--3.17) |
| Cyprus | 3020 (2473-3982) | 412.96 (336.29-540.79) | 2963 (2557-3387) | 251.38 (214.29-297.45) | -1.92 (-2.07--1.76) |
| Czechia | 98209 (88447-112760) | 1073.02 (962.54-1256.93) | 64107 (57834-73845) | 718.97 (641.58-840.43) | -1.22 (-1.35--1.08) |
| C么te d'Ivoire | 10259338 (9551649-11091581) | 85647.29 (78525.12-94286.74) | 11070105 (8324177-14873474) | 38826.35 (29326.36-51841.56) | -2.5 (-2.94--2.06) |
| Democratic People's Republic of Korea | 5919782 (4394521-7500070) | 28604.82 (21195.77-36328.97) | 1832060 (1362874-2467836) | 7578.78 (5638.21-10190.5) | -4.68 (-5.31--4.04) |
| Democratic Republic of the Congo | 32764205 (31713702-33742656) | 85962.23 (83022.82-88839.67) | 52895761 (48439826-57415383) | 57406.61 (52676.16-62029.3) | -1.14 (-1.38--0.89) |
| Denmark | 18307 (15687-22613) | 422.55 (353.04-547.97) | 14207 (12361-18427) | 289.57 (247.32-377.77) | -1.39 (-1.47--1.3) |
| Djibouti | 151576 (125399-178222) | 33278.72 (27332.97-39322.89) | 187884 (154465-229846) | 14241.14 (11716.78-17402.02) | -2.98 (-3.3--2.66) |
| Dominica | 10744 (8219-13592) | 14012.97 (10740.29-17746.15) | 4028 (2986-5450) | 6332.43 (4698.38-8546.75) | -2.92 (-3.98--1.85) |
| Dominican Republic | 2806827 (2300356-3595049) | 38205.55 (31013.75-51422.25) | 2169102 (1698850-2742207) | 19238.37 (15040.5-24226.55) | -3 (-3.59--2.41) |
| Ecuador | 3597185 (2897640-4304471) | 34236.56 (27898.61-40721.61) | 5223352 (3961489-6513089) | 28548.95 (21649.84-35609.91) | -0.66 (-1.01--0.32) |
| Egypt | 23369417 (21811603-26243318) | 42983.9 (40067.48-49571.82) | 12091910 (9365299-15614672) | 11233.86 (8768.67-14371.07) | -4.97 (-5.6--4.34) |
| El Salvador | 2285687 (1887185-2669884) | 39805.29 (32881.68-46387.95) | 1093022 (789642-1453058) | 16628.39 (12038.4-22081.05) | -2.92 (-3.04--2.8) |
| Equatorial Guinea | 316253 (286194-350141) | 73275.28 (65601.98-84283.47) | 444784 (302333-597740) | 28101.85 (19395.96-37651.47) | -3.28 (-3.47--3.08) |
| Eritrea | 1392407 (1172331-1635149) | 38405.21 (32230.85-45361.87) | 1078979 (876192-1355158) | 15216.81 (12394.2-19097.01) | -1.91 (-2.37--1.46) |
| Estonia | 16958 (15486-18571) | 1116.83 (1013.38-1238.02) | 9963 (8953-10981) | 786.32 (711.04-868.72) | -1.31 (-1.4--1.22) |
| Eswatini | 426344 (356139-486004) | 47541.63 (39903.34-54087.61) | 203794 (163640-262302) | 16415.41 (13218.93-21063.1) | -3.39 (-4.07--2.71) |
| Ethiopia | 35657965 (32546390-38581955) | 68383.45 (62545.49-74360.32) | 37219944 (31474568-43039973) | 34569.44 (29597.81-39760.81) | -2.54 (-2.73--2.35) |
| Fiji | 304397 (187128-529055) | 41320.47 (24105.1-75397.56) | 175794 (110434-305688) | 18991.97 (11852.75-33279.45) | -3.03 (-3.62--2.45) |
| Finland | 17785 (15791-20263) | 401.2 (353.77-461.56) | 11977 (10611-13554) | 265.25 (231.25-309.07) | -1.44 (-1.56--1.31) |
| France | 206144 (182662-242497) | 401.37 (346.76-484.23) | 154942 (138740-174065) | 270.32 (238.42-309.94) | -1.38 (-1.49--1.27) |
| Gabon | 635845 (559492-714022) | 62710.78 (54991.84-71262.01) | 581330 (401441-888386) | 30813.69 (21237.26-47027.89) | -1.93 (-2.16--1.69) |
| Gambia | 424645 (367177-489906) | 40171.31 (34557.72-46076.96) | 499648 (412371-625891) | 19181.95 (15849.06-23917.52) | -1.41 (-1.78--1.03) |
| Georgia | 355571 (260252-489398) | 6634.9 (4863.61-9112.7) | 127260 (93653-182265) | 3820.47 (2816.53-5475.82) | -0.98 (-1.48--0.48) |
| Germany | 324002 (220143-486976) | 470.83 (335.27-664.81) | 205682 (150425-327493) | 293.04 (215.81-459.53) | -1.09 (-1.42--0.77) |
| Ghana | 11765456 (10901073-12596744) | 77554.74 (72133.56-83165.58) | 12953015 (11085182-15012548) | 36765.76 (31500.26-42186.26) | -2.11 (-2.51--1.7) |
| Greece | 43339 (37732-51129) | 449.17 (385.81-540.93) | 36873 (32345-42376) | 369.25 (319.94-436.83) | -0.64 (-0.76--0.52) |
| Greenland | 275 (224-363) | 480.09 (396.81-619.33) | 169 (149-196) | 312.65 (272.4-367.65) | -1.5 (-1.57--1.43) |
| Grenada | 17635 (13786-22148) | 18825.68 (14697.56-23678.45) | 6465 (4876-8748) | 6605.07 (4985.66-8918.09) | -3.57 (-4.54--2.58) |
| Guam | 13561 (10033-18010) | 9426.23 (6986.83-12556.74) | 9922 (7098-13391) | 6653.24 (4722.09-9013.72) | -1.15 (-1.37--0.94) |
| Guatemala | 4423584 (3841878-5000903) | 48520.28 (42082.03-55084.86) | 3223816 (2491134-4157821) | 19295.19 (15002.53-24801.36) | -3.07 (-3.24--2.9) |
| Guinea | 4591548 (4272141-4938402) | 75093.04 (69535.21-81122.01) | 6483871 (5221437-7725484) | 45046.23 (36182.4-53710.01) | -1.32 (-1.54--1.1) |
| Guinea-Bissau | 732548 (630044-855244) | 71769.7 (61034.48-89613.12) | 663127 (528312-819124) | 29584.4 (23467.92-36774.54) | -2.68 (-2.99--2.36) |
| Guyana | 424488 (321816-553026) | 57907.12 (42911.07-76752.93) | 206566 (131930-316072) | 27028.54 (17207.13-41491.2) | -2.91 (-3.2--2.62) |
| Haiti | 2912899 (1982419-4490471) | 46713.76 (30147.11-76906.78) | 1839438 (1471926-2293211) | 13531.49 (10850.87-16873.5) | -5.28 (-5.83--4.72) |
| Honduras | 2237094 (1887926-2602363) | 43406.34 (36546.34-50568.84) | 2673959 (2036185-3417371) | 25242.84 (19232.75-32266.29) | -1.6 (-1.69--1.52) |
| Hungary | 120641 (109593-134516) | 1269.32 (1147.55-1432.93) | 75440 (67175-85578) | 873.26 (780.79-994.08) | -1.18 (-1.25--1.1) |
| Iceland | 799 (701-926) | 332.78 (289.57-387.78) | 740 (653-839) | 238.73 (207.06-277.71) | -1.11 (-1.21--1.01) |
| India | 472588576 (415514742-530300698) | 53599.65 (47367.22-59971.81) | 185221591 (139691766-249784928) | 13112.72 (9907.03-17659.25) | -4.8 (-5.15--4.44) |
| Indonesia | 103545467 (86251953-123696222) | 53650.16 (43996.09-66374.94) | 25352015 (19395852-36007931) | 9178.51 (7001.31-13048.3) | -6.21 (-6.55--5.86) |
| Iran (Islamic Republic of) | 4575110 (3392993-6149948) | 7456.71 (5568.71-9994.02) | 5078925 (3805068-6682858) | 6119.88 (4578.44-8049.77) | -1.19 (-1.46--0.91) |
| Iraq | 2629572 (2145855-3282338) | 13994.94 (11476.03-17263.02) | 5499511 (4385051-6854491) | 12822.04 (10279.27-15899.85) | -0.91 (-1.18--0.65) |
| Ireland | 13773 (11705-17490) | 405.34 (342.77-516.03) | 12149 (10837-13758) | 265.7 (233.5-315.43) | -1.67 (-1.81--1.52) |
| Israel | 29583 (25508-36085) | 599.59 (518.04-734.01) | 40873 (35746-48449) | 420.58 (366.38-500.25) | -1.18 (-1.24--1.13) |
| Italy | 162724 (135135-201267) | 351.21 (281.65-449.19) | 117189 (100314-143242) | 245.42 (200.43-328.79) | -1.14 (-1.24--1.04) |
| Jamaica | 359334 (280134-457095) | 14041.52 (10965.46-17867.69) | 192341 (141169-250544) | 7164.47 (5301.8-9293.07) | -2.6 (-3.55--1.63) |
| Japan | 736920 (601170-920405) | 598.46 (478.9-762.57) | 717030 (614245-848166) | 522.66 (433.83-636.42) | -0.22 (-0.45-0.02) |
| Jordan | 270528 (196273-375586) | 6390.68 (4628.81-8852.73) | 908934 (663713-1216090) | 6963.07 (5111.39-9263.31) | -0.76 (-1.16--0.35) |
| Kazakhstan | 1530925 (1148690-2155258) | 9037.34 (6776.57-12735.14) | 731367 (557548-987047) | 3862.26 (2932.47-5226.24) | -2.68 (-2.85--2.5) |
| Kenya | 13497919 (12146290-15287409) | 58240.75 (51764.07-67658.8) | 17044951 (14169100-20048828) | 32651.7 (27486.58-38380.22) | -2.37 (-2.61--2.13) |
| Kiribati | 40764 (23268-55653) | 58185.22 (30944.35-81309.2) | 19671 (14260-26695) | 15526.08 (11272.6-21232.76) | -4.6 (-5.12--4.07) |
| Kuwait | 23215 (21136-25682) | 1229.53 (1125.48-1352.77) | 40452 (35863-47058) | 901.61 (806.82-1049.04) | -0.91 (-1.11--0.72) |
| Kyrgyzstan | 409914 (276292-583316) | 8572.7 (5769.1-12182.26) | 345595 (257318-467733) | 4832.56 (3595.26-6536.63) | -1.61 (-1.8--1.42) |
| Lao People's Democratic Republic | 2426626 (2165085-2707082) | 57913.56 (52009.34-64882.37) | 1744614 (1449650-2080635) | 23327.97 (19429.19-27746) | -2.53 (-2.99--2.07) |
| Latvia | 28908 (26502-31768) | 1128.68 (1026.25-1241.96) | 15404 (13908-17184) | 877.01 (787.5-974.7) | -0.85 (-0.93--0.78) |
| Lebanon | 181375 (135623-246843) | 5739.87 (4289.06-7824.13) | 322378 (226541-456443) | 6022.48 (4237.58-8514.98) | -0.71 (-1.06--0.36) |
| Lesotho | 845474 (725507-964536) | 50236.15 (42993.54-57531.35) | 269247 (190972-362515) | 13329.87 (9456.9-17985.92) | -4.24 (-5.22--3.25) |
| Liberia | 2143894 (1969155-2320829) | 88567.68 (81833.5-95569.18) | 3617583 (3117790-4178742) | 66566.23 (58390.63-75788.69) | -0.85 (-0.93--0.77) |
| Libya | 627876 (513637-761450) | 14517.63 (12107.26-17491.44) | 1008559 (806308-1289596) | 14006.71 (11167.85-17565.52) | -0.5 (-0.62--0.37) |
| Lithuania | 40554 (36710-44884) | 1136.37 (1023.21-1266.55) | 23087 (20957-25377) | 894.55 (808.78-997.34) | -0.73 (-0.76--0.7) |
| Luxembourg | 1325 (1131-1691) | 402.88 (337.71-523.92) | 1416 (1241-1634) | 250.25 (214.47-295.06) | -1.58 (-1.71--1.45) |
| Madagascar | 6780118 (6018017-7659557) | 56908.92 (49743.78-65933.01) | 7005585 (5919023-8125741) | 23441.62 (19732.95-27228.77) | -3.52 (-3.96--3.07) |
| Malawi | 6572856 (5818380-7645484) | 67242.8 (58329.19-82449.87) | 5142256 (3863029-7242924) | 24347.94 (18229.24-34341.85) | -3.73 (-4.19--3.27) |
| Malaysia | 7984969 (6869880-9334922) | 42371.43 (36086.69-51166.57) | 2367645 (1770906-3324713) | 7415.99 (5547.39-10359.85) | -6.74 (-7.31--6.18) |
| Maldives | 111749 (95606-127737) | 46012.31 (39266.84-52779.34) | 22414 (16884-29687) | 4554.8 (3455.07-5956.08) | -8.62 (-9.51--7.72) |
| Mali | 6874922 (6309484-7498612) | 80663.25 (72433.33-90510.82) | 10324521 (8496635-12297389) | 39342.97 (32216.43-47327.12) | -2.34 (-2.69--1.99) |
| Malta | 1649 (1395-2065) | 474.36 (399.99-604.85) | 1146 (1003-1325) | 310.93 (263.23-368.28) | -1.37 (-1.43--1.3) |
| Marshall Islands | 15559 (12619-18894) | 30209.16 (24466.3-36761.19) | 6440 (4993-8328) | 11073.75 (8594.54-14305.79) | -3.22 (-3.32--3.12) |
| Mauritania | 480530 (404367-569273) | 22052.26 (18499.46-26091.91) | 744009 (590759-952338) | 15503.19 (12262.95-19816.06) | -0.33 (-0.74-0.07) |
| Mauritius | 569611 (463406-670903) | 49781.7 (40867.99-58675.31) | 451671 (333968-585613) | 34332.82 (25399.54-44248.67) | -1.17 (-1.37--0.97) |
| Mexico | 22589834 (18842789-26871518) | 24458.44 (20442.56-28975.39) | 19137939 (14224454-25530263) | 14878.95 (11062.33-19849.82) | -1.14 (-1.45--0.82) |
| Micronesia (Federated States of) | 50667 (40671-63245) | 47142.16 (36946.79-62106.84) | 16863 (12289-28836) | 16124.11 (11686.25-28098.06) | -3.35 (-3.62--3.08) |
| Monaco | 102 (87-123) | 326.19 (279.54-392.65) | 115 (96-138) | 268.87 (233.03-315.11) | -0.68 (-0.72--0.65) |
| Mongolia | 464236 (342833-622712) | 19447.91 (14345.9-26111.21) | 226272 (164945-318370) | 6598.79 (4805.75-9298.59) | -3.51 (-3.87--3.15) |
| Montenegro | 6510 (5842-7349) | 1073.46 (959.57-1218.29) | 5201 (4640-5868) | 919.97 (814.27-1035.03) | -0.72 (-0.82--0.63) |
| Morocco | 3403675 (2560130-4440971) | 12495.33 (9391.52-16267.54) | 3845969 (2782480-5185514) | 10379.21 (7498.73-14006.06) | -1.25 (-1.59--0.91) |
| Mozambique | 11721897 (11082567-12330760) | 88715.39 (83138.97-94473.48) | 13261050 (11243846-15300272) | 39328.83 (33315.92-45611.98) | -2.86 (-3.18--2.54) |
| Myanmar | 28703714 (25327405-33487437) | 68601.52 (59469.72-82993.07) | 10363983 (7862394-13428185) | 18141.3 (13777.5-23507.26) | -5.28 (-5.9--4.65) |
| Namibia | 752330 (657353-876223) | 51231.66 (44765.79-59285.5) | 729801 (609184-860078) | 28657.49 (24054.95-33694.87) | -1.54 (-1.66--1.42) |
| Nauru | 2175 (1667-2778) | 19574.06 (14973.71-25097.04) | 1147 (886-1520) | 9668.98 (7477.17-12798.68) | -2.18 (-2.32--2.05) |
| Nepal | 14770824 (14134649-15324977) | 73246.63 (69847.67-76239.59) | 6048892 (4605657-7924670) | 19050.44 (14509.21-24935.64) | -4.52 (-5.09--3.96) |
| Netherlands | 49587 (43411-56425) | 375.65 (325.27-436.62) | 40020 (35355-45341) | 274.75 (238.03-317.55) | -1.18 (-1.27--1.08) |
| New Zealand | 15535 (11996-23380) | 495.72 (368.6-788.56) | 15747 (11908-23835) | 371.57 (255.6-618.23) | -0.87 (-0.94--0.79) |
| Nicaragua | 2313507 (2029918-2613831) | 54184.16 (47375.41-61481.89) | 2380490 (1967428-2777381) | 34323.95 (28467.63-39983.77) | -1.5 (-1.66--1.34) |
| Niger | 5103149 (4315945-6022286) | 64557.3 (53819.17-77730.01) | 10983899 (8533627-13769922) | 39840.54 (30756.96-50364.2) | -1.16 (-1.37--0.95) |
| Nigeria | 70605855 (67117876-74160636) | 78154.81 (73821.03-83062.67) | 108978231 (96453125-122706760) | 45996.82 (40691-51652.09) | -2.13 (-2.34--1.92) |
| Niue | 449 (334-644) | 19023.3 (13977.46-28188.07) | 134 (98-199) | 8247 (6035.51-11929.98) | -2.73 (-3.13--2.33) |
| North Macedonia | 25903 (23536-28779) | 1325.75 (1203.78-1476.47) | 18807 (17151-20904) | 966.32 (878.35-1084.98) | -1.17 (-1.25--1.08) |
| Northern Mariana Islands | 3883 (2879-5116) | 8308.57 (6194.34-10903.1) | 2622 (1944-3458) | 5720.71 (4253.4-7571.97) | -0.9 (-1.12--0.68) |
| Norway | 13206 (11245-16156) | 363.8 (302.88-465.82) | 10595 (9121-12740) | 231.34 (194.74-291.38) | -1.51 (-1.66--1.37) |
| Oman | 203453 (163348-255961) | 11276.39 (9270.89-13698.39) | 332571 (257064-444831) | 7020.85 (5470.58-9326.37) | -1.83 (-1.9--1.75) |
| Pakistan | 67372361 (58466469-74991493) | 56087.24 (48332.78-62885.8) | 36657923 (26018593-50180592) | 14487.85 (10287.19-19815.73) | -4.83 (-5.11--4.54) |
| Palau | 1952 (1487-2538) | 12302.32 (9407.36-15949.04) | 1147 (864-1492) | 7021.36 (5262.11-9110.38) | -1.64 (-1.88--1.41) |
| Palestine | 233109 (179929-305197) | 10046.9 (7754.43-13195.68) | 476709 (326635-645994) | 8384.19 (5773.31-11340.79) | -1.25 (-1.56--0.94) |
| Panama | 660200 (508524-832480) | 25894.39 (20147.68-32539.59) | 709378 (525984-940626) | 16532.21 (12260.85-21921.3) | -1.46 (-1.56--1.36) |
| Papua New Guinea | 3227266 (2752395-3713638) | 79280.56 (66056.72-92230.06) | 3629037 (2919412-4637420) | 34145.49 (27110.01-44517.53) | -3.07 (-3.2--2.95) |
| Paraguay | 1131553 (891922-1390712) | 25785.37 (20399.09-31606.7) | 997063 (736590-1317945) | 13542.79 (10020.51-17832.77) | -1.67 (-1.94--1.4) |
| Peru | 6121212 (5165203-7231887) | 27224.88 (23211.81-31949.5) | 15176686 (13053978-17369525) | 41829.49 (35969.88-47868.94) | 1.88 (1.28-2.48) |
| Philippines | 29794699 (23712993-37386960) | 45653.98 (36234.97-59340.24) | 10215275 (7993150-13052115) | 8833.69 (6934.93-11277.78) | -5.77 (-6.21--5.33) |
| Poland | 544895 (483865-613082) | 1491.6 (1323.61-1679.66) | 352970 (311537-406401) | 1020.72 (890.75-1198.01) | -1.23 (-1.29--1.17) |
| Portugal | 199492 (173427-227430) | 1884.6 (1637.01-2152.24) | 129711 (106221-156551) | 1073.76 (884.52-1293.75) | -2.4 (-2.75--2.04) |
| Puerto Rico | 54621 (45701-68847) | 1546.57 (1294.22-1965.14) | 33295 (26913-44615) | 1134.84 (928.9-1483.25) | -1.1 (-1.18--1.02) |
| Qatar | 20230 (14967-27276) | 4411 (3236.78-5997.75) | 153536 (104304-219795) | 5389.13 (3666.47-7722.16) | -0.38 (-0.81-0.06) |
| Republic of Korea | 7085313 (5331128-9297733) | 15433.81 (11733.63-20124.76) | 4344433 (3434674-5633995) | 8869.08 (6540.29-12043.56) | -0.89 (-1.47--0.31) |
| Republic of Moldova | 68560 (61418-78954) | 1542.91 (1381.71-1774.53) | 37757 (34801-41188) | 1086.75 (1003.08-1204.27) | -1.19 (-1.28--1.1) |
| Romania | 334814 (305688-370245) | 1508.51 (1378.63-1687.49) | 177107 (160758-198927) | 1037.88 (925.77-1187.84) | -1.3 (-1.36--1.24) |
| Russian Federation | 2972886 (2749180-3215549) | 1923.62 (1780.03-2070.93) | 2206523 (2013584-2414401) | 1419.48 (1312.55-1544.2) | -1.15 (-1.28--1.01) |
| Rwanda | 4860107 (4236405-5341163) | 63674.76 (55449.72-70334.87) | 4598214 (3931210-5372886) | 32225.71 (27604.07-37577.8) | -1.82 (-2.19--1.45) |
| Saint Kitts and Nevis | 930 (817-1105) | 2222.51 (1978.57-2593.89) | 828 (731-948) | 1564.39 (1371.06-1828.28) | -1.12 (-1.28--0.96) |
| Saint Lucia | 24392 (18989-31428) | 16391.62 (12778.46-21114.77) | 10161 (7540-13712) | 6354.13 (4699.02-8548.58) | -3.44 (-4.44--2.43) |
| Saint Vincent and the Grenadines | 22882 (18198-28335) | 19015.26 (15105.25-23563.28) | 7592 (5575-10096) | 7061.18 (5201.29-9375.07) | -3.57 (-4.63--2.5) |
| Samoa | 52495 (34662-89096) | 32256.74 (19730.29-58631) | 32221 (20656-63243) | 15176.11 (9385.83-31697.38) | -1.96 (-2.49--1.42) |
| San Marino | 81 (71-94) | 377.51 (327.09-439.83) | 99 (87-114) | 310.11 (270.51-365.5) | -0.61 (-0.73--0.49) |
| Sao Tome and Principe | 60814 (46530-88295) | 46762.52 (34989.46-69863.79) | 47704 (35198-69972) | 20800.6 (15194.98-32209.9) | -2.96 (-3.62--2.3) |
| Saudi Arabia | 1560187 (1173410-2086423) | 9202.19 (6934.73-12213.59) | 2636168 (2023415-3358643) | 6883.7 (5300.42-8764.08) | -1.62 (-1.92--1.32) |
| Senegal | 5087521 (4673515-5521515) | 66847.09 (61131.93-74052.84) | 6027646 (5145718-6913222) | 35767.87 (30793.28-41200.37) | -1.61 (-1.91--1.31) |
| Serbia | 127037 (115078-143511) | 1379.26 (1250.04-1558.29) | 81024 (72841-90608) | 988.71 (880.05-1130.16) | -1.2 (-1.25--1.16) |
| Seychelles | 29855 (26580-33163) | 38299.68 (34084.84-42724.07) | 6930 (5117-9884) | 6990.02 (5165.56-9884.22) | -5.84 (-6.26--5.41) |
| Sierra Leone | 3494262 (3114141-3877703) | 84849.78 (74803.24-95930.25) | 3977576 (2979132-5310610) | 43579.03 (32843.4-57670.79) | -2.15 (-2.46--1.84) |
| Singapore | 39822 (22831-63715) | 1229.52 (699.64-2005.16) | 49802 (20730-94056) | 895.76 (378.76-1664.57) | -0.45 (-1.04-0.14) |
| Slovakia | 65179 (58530-73772) | 1291.8 (1157.58-1466.78) | 47672 (42408-53897) | 941.98 (842.08-1087.03) | -1.06 (-1.09--1.03) |
| Slovenia | 17315 (15680-19779) | 980.58 (875.91-1125.09) | 10925 (9838-12400) | 635.66 (569.36-731.62) | -1.61 (-1.7--1.52) |
| Solomon Islands | 212725 (178622-273070) | 57601.67 (47866.15-75779.97) | 213371 (179970-255249) | 29132.73 (24569.26-34850.29) | -2.51 (-2.64--2.38) |
| Somalia | 6089764 (5769996-6374392) | 72257.68 (68174.89-76227.61) | 12923305 (11215672-14564132) | 54832.04 (47727.89-61769.61) | -0.98 (-1.04--0.93) |
| South Africa | 19981736 (17759088-21916698) | 50890.09 (45149.83-55938.51) | 9997488 (8296074-11875659) | 17240.17 (14325.14-20441.48) | -4.2 (-4.5--3.91) |
| South Sudan | 4122174 (3727917-4579337) | 70091.92 (63876.43-77168.76) | 4901015 (4252617-5785459) | 51700.98 (45639.56-59578.35) | -0.97 (-1.03--0.9) |
| Spain | 181390 (158544-214255) | 504.46 (440.05-637.06) | 194535 (173867-215882) | 398.45 (356.26-463.04) | -0.48 (-0.64--0.32) |
| Sri Lanka | 7933278 (6720194-9110276) | 43967.31 (37094.22-50648.34) | 1556905 (1172871-2138777) | 7304.53 (5468.7-10061.6) | -6.7 (-7.04--6.36) |
| Sudan | 5864883 (4702447-7571461) | 28675.69 (22654.2-39016.7) | 7329939 (5579107-9965643) | 15920.78 (12063.09-21853.76) | -2.32 (-2.54--2.1) |
| Suriname | 121889 (99852-154555) | 30230.61 (24731.39-38543.73) | 105283 (82847-130164) | 18173.33 (14283.36-22543.73) | -1.98 (-2.61--1.35) |
| Sweden | 29189 (25071-36069) | 385.47 (325.29-487.19) | 25049 (21478-30736) | 278.14 (234.55-347.13) | -1.11 (-1.2--1.03) |
| Switzerland | 24962 (17240-38051) | 412.12 (301.01-604.27) | 24888 (18004-39071) | 320.69 (231.71-502.24) | -0.46 (-0.72--0.2) |
| Syrian Arab Republic | 1465702 (1104337-1955302) | 11179.47 (8296.31-14870.93) | 1752675 (1438176-2086584) | 12453.07 (10175.65-14788.24) | -0.17 (-0.38-0.04) |
| Taiwan (Province of China) | 3941584 (2884936-5147141) | 18667.28 (13691.21-24426.18) | 1228868 (815334-1756761) | 6395.62 (4204.06-9160.82) | -3.13 (-3.81--2.44) |
| Tajikistan | 848305 (646929-1126376) | 14415.9 (11064.94-19182.86) | 864575 (633283-1225512) | 8136.01 (5948.25-11530.92) | -1.62 (-1.72--1.53) |
| Thailand | 28441332 (24266433-34620304) | 49618.76 (42419.09-60869.47) | 4712227 (3838202-6089626) | 6999.23 (5667.75-8960.3) | -6.77 (-7.06--6.49) |
| Timor-Leste | 586979 (472623-710061) | 76469.9 (58849.76-94784.69) | 256756 (140837-670057) | 18835.46 (9716.42-53181.41) | -6.2 (-6.99--5.4) |
| Togo | 2872875 (2652292-3059781) | 76555.64 (70522.71-82580.9) | 3040474 (2522572-3677655) | 34128.87 (28288.64-41390.5) | -2.25 (-2.71--1.79) |
| Tokelau | 408 (321-509) | 23770.08 (18695.21-29750.6) | 107 (82-137) | 7785.92 (5991.05-9877.23) | -3.58 (-3.81--3.34) |
| Tonga | 23824 (17692-39288) | 22395.76 (16329.62-40174.81) | 10793 (8004-13935) | 9679.5 (7143.3-12576.55) | -2.55 (-2.71--2.39) |
| Trinidad and Tobago | 180068 (133481-236666) | 14027.26 (10429.24-18405.24) | 88978 (65513-120282) | 7056.13 (5198.72-9478.95) | -2.82 (-3.83--1.79) |
| Tunisia | 629425 (478449-810961) | 7156.95 (5498.84-9258.11) | 898550 (704083-1157800) | 7808.37 (6069.29-10030.11) | -0.32 (-0.61--0.03) |
| Turkey | 5273972 (3889829-7290803) | 8582.72 (6342.01-11832.83) | 5061650 (3632083-7029716) | 6349.61 (4558.69-8815.52) | -1.89 (-2.23--1.55) |
| Turkmenistan | 498540 (371484-682896) | 12649.87 (9483.49-17214.5) | 307143 (228325-420850) | 5956.84 (4441.45-8141.25) | -2.15 (-2.48--1.82) |
| Tuvalu | 2930 (2283-3634) | 29842.04 (23177.64-37119.7) | 1262 (965-1664) | 9967.09 (7623.84-13126.8) | -3.29 (-3.6--2.99) |
| Uganda | 14544434 (13906331-15177683) | 84036.64 (80039.12-88275.54) | 16248534 (14366040-18291968) | 35259.56 (31237.6-39677.49) | -2.51 (-2.95--2.07) |
| Ukraine | 922897 (848950-1003857) | 1707.45 (1580.76-1851.39) | 1117078 (993371-1249811) | 2231.51 (2025.58-2449.63) | 1.41 (1.09-1.74) |
| United Arab Emirates | 113407 (77555-164559) | 5758.88 (3887.54-8405.1) | 480147 (343469-702434) | 5848.31 (4163.27-8542.46) | -0.73 (-1.18--0.28) |
| United Kingdom | 212480 (185648-240006) | 438.26 (377.99-501.76) | 196819 (169881-233861) | 359.92 (302.08-443.78) | -0.61 (-0.74--0.49) |
| United Republic of Tanzania | 21566359 (20726721-22300014) | 82379.57 (78492.41-85941.85) | 19193517 (16251016-22133239) | 30915.88 (26313.69-35681.84) | -3.15 (-3.59--2.71) |
| United States of America | 1121941 (1030946-1222358) | 425.63 (391.37-461.94) | 1563068 (1406578-1742103) | 428.06 (387.07-474.87) | 0.23 (0.15-0.31) |
| United States Virgin Islands | 1692 (1489-2048) | 1572.17 (1386.81-1902.64) | 953 (840-1101) | 1301.94 (1142.28-1563.87) | -0.61 (-0.68--0.54) |
| Uruguay | 67047 (56571-80013) | 2135.12 (1777.77-2577.43) | 39557 (33749-47151) | 1088.87 (883.34-1393.52) | -2.22 (-2.27--2.17) |
| Uzbekistan | 2573931 (1898106-3504719) | 11132.81 (8104.69-15248.82) | 2059501 (1545988-2873898) | 5951.36 (4453.03-8332.3) | -1.77 (-2.1--1.43) |
| Vanuatu | 104358 (83572-137279) | 67821.42 (51478.71-94240.71) | 98770 (75322-137566) | 29823.71 (22516.23-42940.13) | -2.97 (-3.21--2.73) |
| Venezuela (Bolivarian Republic of) | 8577925 (7576464-9609311) | 42616.19 (37737.49-47622.91) | 6343763 (5011798-7761437) | 24555.94 (19303.37-30106.73) | -2.27 (-2.87--1.67) |
| Viet Nam | 47529946 (44105747-53176012) | 66408.58 (60803.88-76769.79) | 14186265 (10129650-19576538) | 14417.75 (10258-19821.42) | -5.6 (-5.87--5.33) |
| Yemen | 4718556 (3865669-5879036) | 34479.96 (28391.17-42187.85) | 6145502 (4765613-7964926) | 16846 (13211.2-21629.17) | -3.13 (-3.45--2.81) |
| Zambia | 5907577 (5409802-6385458) | 73099.69 (66430.6-80982.32) | 6473920 (5378966-7637201) | 31481.91 (26126.73-37228.95) | -2.83 (-3.14--2.52) |
| Zimbabwe | 6094445 (5638491-6572908) | 53747.25 (49491.93-58336.83) | 3594116 (2927629-4322768) | 21510.93 (17549.98-25929.29) | -1.92 (-2.32--1.53) |

**Table S3.** The number of deaths cases and the age-standardized deaths rate of neglected tropical diseases and malaria in 1990 and 2021, and its trends from 1990 to 2021 globally.

|  | Number of deaths cases (95% UI) in 1990 | The age-standardized deaths rate/100000 (95% UI) in 1990 | Number of deaths cases (95% UI) in 2021 | The age-standardized deaths rate/100000 (95% UI) in 2021 | EAPC (95% CI) |
| --- | --- | --- | --- | --- | --- |
| Global | 917420 (558500-1685655) | 16.29 (10.01-29.98) | 847472 (375547-1617956) | 11.8 (5.22-22.48) | -1.49 (-1.8--1.17) |
| Sex |  |  |  |  |  |
| Female | 426708 (256771-792082) | 15.27 (9.26-28.38) | 396719 (175506-750456) | 11.29 (4.95-21.34) | -1.45 (-1.77--1.13) |
| Male | 490712 (302434-888125) | 17.41 (10.74-31.53) | 450754 (200548-874972) | 12.33 (5.5-23.86) | -1.53 (-1.84--1.23) |
| Age |  |  |  |  |  |
| <5 years | 551158 (326338-980694) | 88.91 (52.64-158.19) | 446925 (195623-813005) | 67.9 (29.72-123.52) | -1.39 (-1.78--0.99) |
| 5-9 years | 64437 (37779-124181) | 11.04 (6.47-21.28) | 36234 (16804-68854) | 5.27 (2.45-10.02) | -2.25 (-2.52--1.99) |
| 10-14 years | 33002 (20749-61131) | 6.16 (3.87-11.41) | 21112 (9368-43232) | 3.17 (1.41-6.49) | -2.12 (-2.38--1.85) |
| 15-19 years | 26531 (16225-50515) | 5.11 (3.12-9.73) | 24226 (10302-47253) | 3.88 (1.65-7.57) | -1.27 (-1.46--1.09) |
| 20-24 years | 24014 (14205-45656) | 4.88 (2.89-9.28) | 24969 (10633-48991) | 4.18 (1.78-8.2) | -1.05 (-1.37--0.74) |
| 25-29 years | 20152 (11618-37527) | 4.55 (2.62-8.48) | 21449 (9240-42510) | 3.65 (1.57-7.23) | -1.12 (-1.49--0.76) |
| 30-34 years | 17979 (10247-32911) | 4.66 (2.66-8.54) | 20329 (8776-40850) | 3.36 (1.45-6.76) | -1.24 (-1.51--0.97) |
| 35-39 years | 18567 (10480-35460) | 5.27 (2.98-10.07) | 21947 (9381-45693) | 3.91 (1.67-8.15) | -1.15 (-1.34--0.95) |
| 40-44 years | 17712 (10402-33780) | 6.18 (3.63-11.79) | 23436 (9912-48186) | 4.68 (1.98-9.63) | -1.28 (-1.51--1.05) |
| 45-49 years | 18266 (11131-34810) | 7.87 (4.79-14.99) | 24518 (10794-49891) | 5.18 (2.28-10.54) | -1.69 (-1.92--1.46) |
| 50-54 years | 20509 (12474-39870) | 9.65 (5.87-18.76) | 27620 (11996-58406) | 6.21 (2.7-13.13) | -1.93 (-2.18--1.68) |
| 55-59 years | 22096 (13245-43835) | 11.93 (7.15-23.67) | 30571 (13708-63989) | 7.73 (3.46-16.17) | -1.82 (-2.11--1.53) |
| 60-64 years | 24409 (14226-49754) | 15.2 (8.86-30.98) | 32705 (14045-70021) | 10.22 (4.39-21.88) | -1.91 (-2.32--1.5) |
| 65-69 years | 21789 (12668-43305) | 17.63 (10.25-35.03) | 29438 (12910-62690) | 10.67 (4.68-22.73) | -1.87 (-2.33--1.41) |
| 70-74 years | 21572 (12704-41965) | 25.48 (15.01-49.57) | 32467 (14339-67251) | 15.77 (6.97-32.67) | -1.51 (-1.73--1.28) |
| 75-79 years | 9206 (6314-15974) | 14.96 (10.26-25.95) | 16077 (8269-32114) | 12.19 (6.27-24.35) | -2.07 (-2.71--1.43) |
| 80-84 years | 3763 (2903-5797) | 10.64 (8.21-16.39) | 7966 (5243-12933) | 9.1 (5.99-14.77) | -1.77 (-2.61--0.94) |
| 85-89 years | 1619 (1406-1904) | 10.71 (9.3-12.6) | 3557 (2882-4486) | 7.78 (6.3-9.81) | -1.33 (-1.82--0.84) |
| 90-94 years | 495 (404-577) | 11.55 (9.42-13.46) | 1356 (1085-1585) | 7.58 (6.06-8.86) | -0.94 (-1.16--0.73) |
| 95+ years | 145 (111-174) | 14.28 (10.86-17.05) | 568 (420-702) | 10.43 (7.7-12.88) | -0.23 (-0.56-0.1) |
| SDI region | |  |  |  |  |
| High-middle SDI | 6049 (4617-11318) | 0.63 (0.49-1.16) | 3780 (2995-4551) | 0.28 (0.22-0.34) | -2.4 (-2.57--2.23) |
| High SDI | 640 (544-1001) | 0.07 (0.06-0.12) | 830 (699-920) | 0.05 (0.04-0.05) | -0.96 (-1.22--0.71) |
| Low-middle SDI | 242354 (136217-510966) | 19.82 (11.41-40.63) | 206205 (98535-402438) | 11.68 (5.7-22.7) | -2.09 (-2.43--1.76) |
| Low SDI | 560541 (346894-932905) | 90.83 (55.84-154.31) | 556497 (226355-1086527) | 50.04 (19.99-100.02) | -2.37 (-2.59--2.14) |
| Middle SDI | 107387 (64747-214642) | 6.75 (4.27-12.99) | 79830 (46166-134090) | 3.64 (2.09-6.01) | -2.2 (-2.51--1.89) |
| GBD region | |  |  |  |  |
| Advanced Health System | 1991 (1374-5966) | 0.15 (0.1-0.49) | 1308 (1140-1504) | 0.06 (0.05-0.07) | -3.08 (-3.35--2.8) |
| Africa | 685504 (414589-1092881) | 92.37 (56.81-149.65) | 749223 (307888-1443663) | 56.09 (22.76-112.5) | -2.23 (-2.57--1.88) |
| African Region | 665899 (401362-1064550) | 110.48 (67.31-179.49) | 740278 (303328-1426640) | 67.6 (27.19-136.6) | -2.19 (-2.54--1.83) |
| America | 24877 (20215-32925) | 3.76 (3.14-4.85) | 13781 (11481-17392) | 1.15 (0.93-1.52) | -3.99 (-4.21--3.76) |
| Andean Latin America | 2771 (1561-5278) | 9.14 (5.69-15.92) | 704 (509-964) | 1.18 (0.85-1.61) | -6.73 (-7.16--6.31) |
| Asia | 205022 (98165-530211) | 6.29 (3.09-16.24) | 83481 (51543-163060) | 1.91 (1.16-3.72) | -3.35 (-3.76--2.93) |
| Australasia | 7 (6-8) | 0.03 (0.03-0.04) | 8 (6-9) | 0.01 (0.01-0.02) | -2.56 (-3.23--1.89) |
| Basic Health System | 56577 (39952-116462) | 2.92 (2.22-5.59) | 32917 (27249-39376) | 1.1 (0.9-1.34) | -2.92 (-3.05--2.8) |
| Caribbean | 1091 (557-2264) | 3.15 (1.65-6.49) | 968 (275-2704) | 2.02 (0.57-5.66) | -3.72 (-5.06--2.36) |
| Central Africa | 134256 (83185-205028) | 147.62 (91.55-224.77) | 129501 (59591-245534) | 78.21 (34.77-153.96) | -2.87 (-3.31--2.42) |
| Central Asia | 433 (137-1054) | 0.51 (0.19-1.18) | 141 (88-408) | 0.15 (0.1-0.43) | -3.91 (-4.42--3.38) |
| Central Europe | 262 (99-1417) | 0.22 (0.08-1.24) | 64 (49-106) | 0.04 (0.03-0.09) | -6.51 (-6.91--6.11) |
| Central Latin America | 4053 (3650-4896) | 3.27 (2.98-3.71) | 2638 (1984-3380) | 1.09 (0.82-1.38) | -3.7 (-4.22--3.17) |
| Central Sub-Saharan Africa | 109608 (68898-164339) | 144.96 (92.49-215.79) | 94331 (42998-175450) | 65.63 (28.98-130.63) | -3.46 (-3.93--2.99) |
| Commonwealth High Income | 53 (46-66) | 0.04 (0.04-0.05) | 52 (44-60) | 0.02 (0.02-0.03) | -1.73 (-1.92--1.53) |
| Commonwealth Low Income | 190711 (115170-335213) | 77.13 (44.15-142.78) | 126204 (47298-239491) | 32.38 (12.18-62.97) | -4.37 (-4.87--3.87) |
| Commonwealth Middle Income | 344736 (191839-705766) | 27.24 (15.13-55.28) | 345665 (141349-708880) | 17.94 (7.45-36.68) | -1.48 (-1.73--1.22) |
| East Asia | 7772 (5444-19388) | 0.76 (0.56-1.7) | 2314 (1701-3095) | 0.13 (0.09-0.19) | -5.04 (-5.64--4.43) |
| East Asia & Pacific - WB | 36617 (22288-95899) | 2.07 (1.3-5.33) | 21499 (17035-26096) | 1.06 (0.81-1.3) | -1.38 (-1.96--0.81) |
| Eastern Africa | 164377 (116220-247696) | 78.89 (57.31-118.04) | 108908 (45046-201658) | 27.55 (11.83-51.47) | -4.8 (-5.34--4.26) |
| Eastern Europe | 252 (203-303) | 0.11 (0.1-0.13) | 159 (137-180) | 0.07 (0.06-0.08) | -2.1 (-2.33--1.86) |
| Eastern Mediterranean Region | 42632 (19109-117041) | 11.37 (5.06-34.22) | 29439 (9977-68217) | 4.27 (1.51-9.94) | -3.55 (-4.2--2.9) |
| Eastern Sub-Saharan Africa | 238254 (160408-366451) | 104.45 (70.49-161.13) | 162596 (65542-308411) | 39.6 (15.9-78.03) | -4.45 (-4.93--3.97) |
| Europe | 1552 (956-5521) | 0.19 (0.11-0.76) | 649 (549-941) | 0.05 (0.04-0.1) | -4.1 (-4.26--3.93) |
| Europe & Central Asia - WB | 1945 (1178-5928) | 0.24 (0.14-0.77) | 740 (617-1178) | 0.06 (0.05-0.12) | -4.35 (-4.62--4.09) |
| European Region | 1951 (1180-5967) | 0.24 (0.14-0.77) | 752 (628-1202) | 0.06 (0.05-0.13) | -4.33 (-4.6--4.06) |
| High-income Asia Pacific | 74 (68-79) | 0.04 (0.04-0.05) | 128 (65-155) | 0.03 (0.01-0.03) | -1.32 (-1.53--1.12) |
| High-income North America | 113 (102-122) | 0.04 (0.03-0.04) | 386 (346-418) | 0.07 (0.06-0.08) | 3.18 (2.69-3.67) |
| Latin America & Caribbean - WB | 24780 (20115-32833) | 7.45 (6.55-9.2) | 13401 (11113-16995) | 1.97 (1.63-2.55) | -4.4 (-4.62--4.18) |
| Limited Health System | 601590 (352747-1149335) | 34.7 (20.48-66.06) | 535802 (220217-1070233) | 19.85 (8.32-39.76) | -2.28 (-2.57--1.98) |
| Middle East & North Africa - WB | 9941 (4033-22787) | 4.39 (1.99-9.69) | 10414 (2989-22921) | 2.37 (0.71-5.3) | -2.13 (-3.06--1.2) |
| Minimal Health System | 256812 (154730-418083) | 145.74 (87.14-238.43) | 277113 (126606-517779) | 86.13 (36.92-169.62) | -2.1 (-2.33--1.87) |
| North Africa and Middle East | 18718 (8698-43125) | 5.88 (2.9-12.09) | 15298 (5413-32612) | 2.62 (0.95-5.59) | -3.2 (-3.9--2.49) |
| North America | 113 (102-122) | 0.04 (0.03-0.04) | 386 (346-418) | 0.07 (0.06-0.08) | 3.18 (2.69-3.67) |
| Northern Africa | 2177 (1409-6554) | 2.5 (1.8-5.53) | 1132 (765-1600) | 0.66 (0.47-0.92) | -3.28 (-3.94--2.62) |
| Oceania | 2783 (484-8833) | 50.19 (8.48-168.31) | 2507 (1066-4772) | 20.68 (8.85-39.81) | -2.45 (-3.27--1.62) |
| Region of the Americas | 24877 (20215-32925) | 3.76 (3.14-4.85) | 13781 (11481-17392) | 1.15 (0.93-1.52) | -3.99 (-4.21--3.76) |
| South-East Asia Region | 165598 (82763-390462) | 11.82 (6.05-27.63) | 54629 (37927-100454) | 3 (2.13-5.38) | -3.78 (-4.27--3.28) |
| South Asia | 158558 (71867-402721) | 13.28 (6.25-33.52) | 51938 (27990-111687) | 3.22 (1.87-6.64) | -4.06 (-4.53--3.59) |
| South Asia - WB | 160079 (72526-405065) | 13.1 (6.16-32.92) | 52340 (28357-112077) | 3.15 (1.83-6.46) | -4.1 (-4.56--3.64) |
| Southeast Asia | 27105 (15364-70577) | 5.86 (3.3-16.16) | 16905 (12000-21548) | 2.68 (1.91-3.42) | -1.7 (-2.23--1.16) |
| Southern Africa | 80965 (50404-131909) | 75.89 (46.06-126.05) | 66081 (24604-138903) | 38.46 (13.96-83.2) | -3.33 (-3.73--2.92) |
| Southern Latin America | 1149 (810-1530) | 2.64 (1.84-3.45) | 487 (326-689) | 0.58 (0.38-0.82) | -4.54 (-4.81--4.27) |
| Southern Sub-Saharan Africa | 3717 (1631-7689) | 6.73 (3.06-14.32) | 2570 (1540-4658) | 3.38 (2.08-5.98) | -0.06 (-1.12-1) |
| Sub-Saharan Africa - WB | 683485 (412858-1090857) | 112.9 (69.22-183.25) | 748360 (306957-1442869) | 68.18 (27.51-138.13) | -2.23 (-2.57--1.88) |
| Tropical Latin America | 15753 (12947-20003) | 14.34 (12.78-16.99) | 8637 (6997-11616) | 3.54 (2.81-4.96) | -4.49 (-4.66--4.31) |
| Western Africa | 303729 (162058-518567) | 152.05 (80.87-263.06) | 443601 (179034-854442) | 114.14 (44.69-230.93) | -1.23 (-1.6--0.85) |
| Western Europe | 370 (283-767) | 0.08 (0.06-0.21) | 256 (210-366) | 0.03 (0.02-0.06) | -3.05 (-3.18--2.92) |
| Western Pacific Region | 15985 (10585-40272) | 1.13 (0.78-2.72) | 8225 (6919-9727) | 0.45 (0.38-0.54) | -2.3 (-2.76--1.85) |
| Western Sub-Saharan Africa | 324577 (173810-551889) | 147.05 (78.87-253.43) | 484439 (196147-939598) | 111.74 (43.53-226.48) | -1.24 (-1.62--0.86) |
| World Bank High Income | 1050 (813-2281) | 0.1 (0.07-0.24) | 1022 (873-1174) | 0.05 (0.04-0.06) | -2.01 (-2.29--1.73) |
| World Bank Low Income | 397450 (253343-630757) | 96.62 (62.74-153.32) | 370043 (158653-699078) | 50.74 (20.85-99.73) | -2.63 (-2.87--2.38) |
| World Bank Lower Middle Income | 481515 (262244-992800) | 23.02 (12.6-47.9) | 458732 (203019-905878) | 14.99 (6.72-29.49) | -1.71 (-2.01--1.41) |
| World Bank Upper Middle Income | 36948 (28512-59524) | 2.14 (1.75-3.23) | 17345 (14725-21571) | 0.61 (0.49-0.79) | -4.04 (-4.14--3.94) |
| Country |  |  |  |  |  |
| Afghanistan | 622 (112-3794) | 6.07 (1.11-34.68) | 179 (114-246) | 0.72 (0.45-0.99) | -6.12 (-8.31--3.88) |
| Albania | 25 (7-156) | 0.85 (0.28-4.53) | 5 (2-25) | 0.19 (0.05-1.06) | -5.85 (-6.87--4.82) |
| Algeria | 150 (87-542) | 0.79 (0.52-2.09) | 73 (54-114) | 0.22 (0.16-0.31) | -4.1 (-4.19--4.01) |
| American Samoa | 0 (0-0) | 0.62 (0.44-0.82) | 0 (0-0) | 0.66 (0.43-0.91) | 0.75 (0.51-0.99) |
| Andorra | 0 (0-0) | 0.04 (0.03-0.06) | 0 (0-0) | 0.01 (0.01-0.02) | -3.4 (-3.58--3.22) |
| Angola | 10482 (6051-17190) | 95.27 (55.47-152.02) | 19287 (8145-39486) | 66.18 (25.76-142.48) | -3.82 (-4.83--2.8) |
| Antigua and Barbuda | 0 (0-0) | 0.39 (0.2-0.63) | 0 (0-0) | 0.34 (0.25-0.45) | -0.81 (-1.28--0.34) |
| Argentina | 1073 (741-1450) | 3.57 (2.44-4.78) | 412 (263-605) | 0.76 (0.49-1.13) | -4.66 (-4.93--4.38) |
| Armenia | 2 (1-10) | 0.06 (0.02-0.27) | 3 (0-17) | 0.09 (0.01-0.64) | 0.77 (0.26-1.27) |
| Australia | 6 (5-7) | 0.03 (0.03-0.04) | 7 (6-8) | 0.02 (0.01-0.02) | -2.3 (-3.03--1.56) |
| Austria | 4 (1-24) | 0.06 (0.01-0.37) | 5 (3-15) | 0.04 (0.02-0.19) | -0.81 (-1.18--0.45) |
| Azerbaijan | 12 (7-31) | 0.19 (0.11-0.43) | 11 (3-53) | 0.11 (0.03-0.55) | -2.29 (-2.56--2.02) |
| Bahamas | 0 (0-0) | 0.08 (0.05-0.12) | 0 (0-0) | 0.08 (0.06-0.1) | 0.13 (-0.25-0.52) |
| Bahrain | 0 (0-0) | 0.06 (0.05-0.08) | 0 (0-0) | 0.04 (0.02-0.04) | -4.07 (-5.06--3.07) |
| Bangladesh | 16865 (2594-54376) | 16.35 (2.44-57.23) | 1051 (652-1642) | 0.72 (0.49-1.07) | -8.47 (-9.75--7.16) |
| Barbados | 0 (0-0) | 0.1 (0.07-0.14) | 2 (1-2) | 0.39 (0.28-0.54) | 5.42 (4.22-6.64) |
| Belarus | 6 (4-8) | 0.05 (0.04-0.08) | 4 (3-5) | 0.04 (0.03-0.04) | -1.75 (-2.02--1.49) |
| Belgium | 14 (8-21) | 0.09 (0.06-0.14) | 4 (3-5) | 0.02 (0.01-0.02) | -2.79 (-3.87--1.71) |
| Belize | 1 (1-2) | 0.89 (0.61-1.48) | 1 (1-1) | 0.23 (0.18-0.27) | -4.25 (-4.47--4.03) |
| Benin | 6098 (3511-9251) | 103.25 (58.02-156.96) | 16395 (8029-28852) | 137.47 (62.31-261.21) | 0.9 (0.36-1.44) |
| Bermuda | 0 (0-0) | 0.07 (0.05-0.1) | 0 (0-0) | 0.02 (0.01-0.03) | -4.49 (-4.71--4.28) |
| Bhutan | 618 (104-1704) | 82.74 (13.54-250.06) | 6 (3-11) | 0.91 (0.47-1.61) | -15.48 (-16.77--14.17) |
| Bolivia (Plurinational State of) | 763 (497-1191) | 21.76 (14.7-31.31) | 438 (287-679) | 5.08 (3.32-7.81) | -4.56 (-4.61--4.5) |
| Bosnia and Herzegovina | 10 (3-60) | 0.24 (0.07-1.4) | 2 (1-4) | 0.04 (0.02-0.1) | -6.14 (-6.87--5.4) |
| Botswana | 38 (27-51) | 4.72 (3.15-6.44) | 36 (26-50) | 1.95 (1.4-2.66) | -3.87 (-4.57--3.16) |
| Brazil | 15646 (12844-19906) | 14.61 (13.01-17.34) | 8547 (6938-11473) | 3.59 (2.85-5.05) | -4.52 (-4.7--4.34) |
| Brunei Darussalam | 0 (0-1) | 0.4 (0.14-0.99) | 0 (0-1) | 0.32 (0.08-0.88) | 0.39 (-0.34-1.13) |
| Bulgaria | 42 (11-257) | 0.5 (0.11-3.44) | 13 (9-18) | 0.12 (0.08-0.17) | -5.12 (-5.67--4.57) |
| Burkina Faso | 21704 (11792-37186) | 204.41 (108.56-363.82) | 37323 (17605-65409) | 179.82 (83.53-325.46) | -0.77 (-1.29--0.24) |
| Burundi | 14623 (7595-22475) | 185.11 (97.96-298.65) | 14098 (6407-26503) | 96.6 (43.47-198.02) | -4.28 (-5.17--3.39) |
| Cabo Verde | 7 (3-11) | 1.68 (0.8-2.97) | 5 (2-9) | 1.04 (0.47-1.91) | -1.6 (-2.06--1.13) |
| Cambodia | 1430 (745-2969) | 12.78 (6.55-26.46) | 145 (79-220) | 1.01 (0.58-1.46) | -6.03 (-7.52--4.51) |
| Cameroon | 14210 (8099-22595) | 128.7 (71.86-209.74) | 30336 (12689-59782) | 121.73 (50.7-250.07) | -1.28 (-1.96--0.6) |
| Canada | 7 (6-8) | 0.02 (0.02-0.03) | 21 (18-24) | 0.03 (0.03-0.04) | 0.54 (0.05-1.04) |
| Central African Republic | 3503 (2225-5411) | 119.3 (78.02-175.63) | 5515 (2285-12120) | 111.99 (46.02-246.96) | -0.34 (-0.92-0.24) |
| Chad | 6246 (3412-11216) | 86.31 (46.93-152.94) | 10008 (3820-19650) | 62.1 (24.25-124.91) | -1.88 (-2.19--1.58) |
| Chile | 61 (47-76) | 0.63 (0.48-0.8) | 68 (45-98) | 0.27 (0.18-0.38) | -2.29 (-2.5--2.09) |
| China | 7726 (5394-19353) | 0.79 (0.58-1.76) | 2235 (1640-3001) | 0.13 (0.09-0.19) | -5.16 (-5.78--4.53) |
| Colombia | 1054 (608-1864) | 3.24 (1.94-5.59) | 396 (313-493) | 0.79 (0.62-0.98) | -5.01 (-5.81--4.19) |
| Comoros | 188 (9-906) | 44.16 (2.06-220.68) | 36 (18-55) | 5.3 (2.65-8.04) | -3.54 (-6.75--0.23) |
| Congo | 3456 (2116-5192) | 158.37 (96.36-237.3) | 2655 (1122-4982) | 59.12 (24.91-113.66) | -4.28 (-4.85--3.7) |
| Cook Islands | 7 (6-8) | 36.66 (33.33-40.38) | 0 (0-0) | 0.18 (0.08-0.26) | -4.75 (-7.55--1.86) |
| Costa Rica | 8 (7-10) | 0.32 (0.26-0.39) | 3 (2-3) | 0.05 (0.04-0.08) | -6.4 (-7.02--5.78) |
| Croatia | 4 (2-12) | 0.07 (0.04-0.28) | 1 (1-3) | 0.02 (0.01-0.08) | -4.94 (-5.47--4.4) |
| Cuba | 19 (13-25) | 0.21 (0.15-0.29) | 4 (3-5) | 0.03 (0.02-0.04) | -6.83 (-8.01--5.63) |
| Cyprus | 1 (0-5) | 0.19 (0.03-0.75) | 1 (0-2) | 0.04 (0-0.18) | -5.31 (-5.6--5.03) |
| Czechia | 4 (3-5) | 0.03 (0.02-0.04) | 3 (2-4) | 0.01 (0.01-0.02) | -1.89 (-2.39--1.39) |
| C么te d'Ivoire | 21414 (11508-34704) | 174.65 (89.59-296.64) | 28883 (12348-53163) | 127.47 (52.74-247.53) | -1.55 (-2.28--0.81) |
| Democratic People's Republic of Korea | 35 (21-49) | 0.18 (0.11-0.25) | 49 (28-71) | 0.17 (0.1-0.25) | -0.1 (-0.44-0.24) |
| Democratic Republic of the Congo | 90415 (56846-133956) | 159.45 (102.49-234.73) | 65086 (30348-114368) | 63.64 (29.2-117.55) | -3.51 (-3.97--3.05) |
| Denmark | 2 (1-2) | 0.02 (0.02-0.03) | 3 (3-4) | 0.03 (0.02-0.03) | 0.38 (-0.34-1.09) |
| Djibouti | 172 (100-288) | 40.21 (22.94-72.21) | 205 (118-306) | 18.31 (10.55-27.24) | -3.18 (-6.42-0.17) |
| Dominica | 0 (0-0) | 0.23 (0.15-0.32) | 0 (0-0) | 0.18 (0.09-0.25) | -0.38 (-0.68--0.07) |
| Dominican Republic | 72 (58-86) | 1.35 (1.06-1.7) | 62 (46-97) | 0.59 (0.44-0.92) | -2.61 (-3.12--2.1) |
| Ecuador | 626 (350-1083) | 6.95 (4.28-11.38) | 80 (64-103) | 0.5 (0.4-0.64) | -8.78 (-10.18--7.34) |
| Egypt | 1411 (847-4824) | 4.01 (2.78-9.35) | 404 (310-533) | 0.61 (0.47-0.79) | -4.79 (-5.17--4.4) |
| El Salvador | 60 (49-71) | 1.35 (1.12-1.59) | 39 (30-50) | 0.61 (0.47-0.78) | -2.27 (-2.55--1.99) |
| Equatorial Guinea | 622 (316-1125) | 116.11 (59.59-211.36) | 941 (401-1958) | 79.75 (32.05-182.92) | -2.39 (-2.78--1.99) |
| Eritrea | 815 (504-1309) | 26.06 (17.64-42.36) | 382 (268-540) | 7.77 (5.78-10.42) | -3.25 (-3.94--2.56) |
| Estonia | 0 (0-0) | 0.02 (0.01-0.02) | 0 (0-0) | 0.01 (0.01-0.02) | 0.02 (-1.54-1.6) |
| Eswatini | 47 (18-136) | 7.4 (3.03-20.78) | 21 (14-31) | 2.45 (1.6-3.55) | -3.36 (-4.11--2.6) |
| Ethiopia | 23480 (17754-29905) | 48.35 (38.48-60.23) | 9918 (5218-17776) | 9.08 (5.56-14.53) | -4.81 (-5.69--3.92) |
| Fiji | 5 (3-10) | 1.34 (0.76-4.25) | 6 (4-16) | 1.06 (0.59-3.69) | -0.55 (-0.86--0.24) |
| Finland | 0 (0-1) | 0.01 (0.01-0.01) | 1 (0-1) | 0.01 (0-0.01) | -1.76 (-3.48--0.01) |
| France | 59 (45-126) | 0.09 (0.06-0.22) | 28 (22-39) | 0.02 (0.02-0.04) | -4.69 (-5.04--4.35) |
| Gabon | 1129 (637-1801) | 115.41 (65.79-186.2) | 847 (321-1949) | 55.41 (21-129.66) | -2.41 (-2.75--2.06) |
| Gambia | 869 (366-1578) | 104.19 (41.76-190.27) | 650 (440-989) | 41.61 (27.53-63.9) | -1.19 (-2.64-0.29) |
| Georgia | 15 (5-72) | 0.27 (0.1-1.37) | 33 (17-94) | 0.78 (0.37-2.76) | 3.59 (2.82-4.37) |
| Germany | 32 (25-41) | 0.03 (0.02-0.04) | 44 (37-51) | 0.02 (0.02-0.03) | -0.55 (-1.2-0.1) |
| Ghana | 16716 (8582-28033) | 96.52 (48.29-167.23) | 16943 (8321-30744) | 58.22 (28.06-111.55) | -1.26 (-2.17--0.34) |
| Greece | 16 (6-70) | 0.14 (0.05-0.76) | 14 (6-51) | 0.09 (0.02-0.58) | -0.93 (-1.29--0.58) |
| Greenland | 0 (0-0) | 0.13 (0.07-0.16) | 0 (0-0) | 0.13 (0.05-0.18) | 0.87 (0.6-1.14) |
| Grenada | 0 (0-0) | 0.45 (0.35-0.57) | 0 (0-0) | 0.27 (0.23-0.32) | 0.53 (-0.28-1.35) |
| Guam | 0 (0-0) | 0.07 (0.05-0.09) | 0 (0-0) | 0.03 (0.02-0.05) | -1.32 (-1.7--0.94) |
| Guatemala | 576 (522-634) | 7.47 (6.81-8.13) | 135 (95-185) | 1.03 (0.73-1.39) | -6.55 (-7.43--5.66) |
| Guinea | 8876 (5021-16621) | 104.25 (59.68-192.53) | 13693 (6167-25551) | 99.89 (43.46-198.48) | 0.29 (-0.19-0.78) |
| Guinea-Bissau | 1392 (831-2503) | 110.82 (69.06-195.19) | 693 (234-1435) | 40.62 (14.91-84.37) | -4.71 (-5.41--4.01) |
| Guyana | 210 (87-475) | 29.75 (12.37-68.66) | 64 (29-107) | 8.42 (3.99-14.02) | -5.89 (-7.36--4.4) |
| Haiti | 674 (311-1351) | 10.5 (4.51-21.64) | 767 (141-2396) | 6.5 (1.24-20.28) | -4.55 (-6.19--2.89) |
| Honduras | 90 (61-112) | 2.01 (1.55-2.37) | 95 (48-153) | 1.25 (0.73-1.89) | -1.12 (-1.65--0.59) |
| Hungary | 6 (4-8) | 0.05 (0.04-0.06) | 6 (5-8) | 0.04 (0.03-0.05) | -1.74 (-2.33--1.14) |
| Iceland | 0 (0-0) | 0.02 (0.01-0.02) | 0 (0-0) | 0.02 (0.02-0.02) | 0.06 (-1.7-1.85) |
| India | 124306 (65370-270342) | 13.19 (7.32-27.41) | 39196 (23113-83167) | 3.21 (1.95-6.59) | -3.91 (-4.45--3.36) |
| Indonesia | 13767 (6877-42149) | 7.56 (3.82-24.78) | 11889 (8107-15623) | 5.06 (3.44-6.61) | -0.89 (-1.21--0.57) |
| Iran (Islamic Republic of) | 725 (122-3864) | 1.28 (0.24-6.87) | 81 (59-149) | 0.11 (0.08-0.19) | -7.02 (-8.03--5.99) |
| Iraq | 481 (131-3086) | 2.93 (1.1-14.55) | 107 (67-285) | 0.38 (0.25-0.79) | -7.75 (-8.27--7.22) |
| Ireland | 1 (1-1) | 0.03 (0.02-0.03) | 1 (1-1) | 0.01 (0.01-0.01) | -3.44 (-4.39--2.48) |
| Israel | 2 (1-6) | 0.04 (0.02-0.12) | 11 (9-13) | 0.08 (0.07-0.1) | 1.92 (0.95-2.89) |
| Italy | 78 (66-92) | 0.12 (0.1-0.14) | 61 (52-69) | 0.06 (0.05-0.06) | -3.61 (-4.1--3.12) |
| Jamaica | 9 (5-15) | 0.5 (0.28-0.81) | 13 (10-17) | 0.43 (0.32-0.57) | 2.51 (1.27-3.76) |
| Japan | 38 (35-40) | 0.03 (0.03-0.03) | 46 (37-53) | 0.01 (0.01-0.01) | -2.27 (-3.35--1.19) |
| Jordan | 18 (7-99) | 0.7 (0.35-2.38) | 7 (5-11) | 0.1 (0.07-0.14) | -6.57 (-6.86--6.27) |
| Kazakhstan | 14 (10-23) | 0.09 (0.06-0.14) | 16 (10-28) | 0.09 (0.06-0.15) | -0.45 (-1.03-0.14) |
| Kenya | 12740 (8078-21101) | 44.33 (26.3-76.99) | 8414 (5292-12775) | 19.19 (12.03-28.73) | -5.7 (-6.9--4.48) |
| Kiribati | 1 (1-1) | 2.11 (1.61-2.84) | 1 (1-2) | 2.45 (1.47-3.28) | 0.6 (0.43-0.76) |
| Kuwait | 1 (0-1) | 0.06 (0.03-0.09) | 0 (0-0) | 0.01 (0.01-0.01) | -6.42 (-7.73--5.09) |
| Kyrgyzstan | 17 (11-29) | 0.35 (0.24-0.58) | 21 (11-64) | 0.3 (0.17-0.88) | -0.91 (-1.23--0.59) |
| Lao People's Democratic Republic | 191 (131-321) | 4.78 (3.33-7.84) | 58 (37-84) | 0.95 (0.62-1.34) | -5.38 (-6.83--3.91) |
| Latvia | 2 (2-2) | 0.09 (0.08-0.11) | 1 (1-2) | 0.05 (0.04-0.07) | -1.66 (-2.05--1.26) |
| Lebanon | 15 (4-81) | 0.55 (0.18-2.6) | 5 (3-7) | 0.08 (0.05-0.13) | -5.77 (-6.15--5.4) |
| Lesotho | 13 (9-19) | 1.04 (0.7-1.47) | 17 (11-23) | 1.11 (0.72-1.52) | 0.86 (0.48-1.24) |
| Liberia | 5549 (2602-9902) | 182.61 (84.94-332.26) | 5893 (2103-11439) | 142.13 (49.51-286.67) | -1.74 (-2.49--0.99) |
| Libya | 30 (15-111) | 1.05 (0.58-2.69) | 24 (15-53) | 0.43 (0.27-0.91) | -2.48 (-2.92--2.04) |
| Lithuania | 1 (1-1) | 0.03 (0.02-0.03) | 2 (1-2) | 0.04 (0.03-0.04) | 1.31 (0.7-1.91) |
| Luxembourg | 0 (0-0) | 0.02 (0.02-0.02) | 0 (0-1) | 0.04 (0.03-0.05) | 2.33 (0.35-4.35) |
| Madagascar | 6120 (3758-9505) | 46.86 (28.94-71.61) | 7144 (1984-15055) | 29.44 (9.11-61.68) | -1.87 (-2.6--1.14) |
| Malawi | 23562 (14952-37800) | 183.2 (111.05-299.19) | 9011 (3630-18240) | 55.39 (21.98-117.29) | -5.03 (-5.47--4.59) |
| Malaysia | 252 (184-371) | 1.58 (1.2-2.26) | 236 (123-379) | 0.77 (0.41-1.21) | -2.71 (-3.33--2.1) |
| Maldives | 4 (2-7) | 1.18 (0.61-2.29) | 2 (1-4) | 0.57 (0.27-1) | -1.48 (-2.24--0.71) |
| Mali | 20632 (11185-36052) | 175.85 (96.51-313.05) | 24955 (11847-49493) | 97.35 (45.04-203.03) | -1.78 (-2.05--1.52) |
| Malta | 1 (0-10) | 0.42 (0.04-3) | 0 (0-3) | 0.11 (0-0.88) | -5.05 (-5.62--4.47) |
| Marshall Islands | 0 (0-0) | 0.57 (0.25-0.77) | 0 (0-0) | 0.54 (0.19-0.8) | -0.2 (-0.41-0.02) |
| Mauritania | 331 (126-836) | 17.42 (7.18-43.91) | 474 (162-955) | 13.65 (4.56-27.78) | -0.9 (-2.84-1.08) |
| Mauritius | 8 (5-12) | 1.07 (0.62-1.55) | 6 (4-8) | 0.36 (0.25-0.48) | -3.49 (-3.85--3.12) |
| Mexico | 932 (851-1022) | 1.2 (1.11-1.3) | 342 (295-392) | 0.28 (0.24-0.32) | -3.81 (-4.54--3.08) |
| Micronesia (Federated States of) | 0 (0-1) | 0.81 (0.46-1.16) | 0 (0-0) | 0.62 (0.26-0.91) | -0.85 (-0.89--0.8) |
| Monaco | 0 (0-0) | 0.05 (0.03-0.06) | 0 (0-0) | 0.42 (0.31-0.55) | 7.8 (5.8-9.85) |
| Mongolia | 7 (2-18) | 0.28 (0.1-0.66) | 3 (1-6) | 0.08 (0.03-0.19) | -4.63 (-4.91--4.35) |
| Montenegro | 1 (0-6) | 0.12 (0.02-0.98) | 1 (0-4) | 0.09 (0.01-0.77) | -0.91 (-1.99-0.18) |
| Morocco | 201 (150-375) | 1.06 (0.84-1.65) | 133 (94-233) | 0.4 (0.28-0.67) | -2.97 (-3.08--2.86) |
| Mozambique | 33397 (20363-55982) | 223.31 (128.34-384.69) | 28206 (9392-61174) | 119.38 (38.59-267.5) | -2.82 (-3.13--2.52) |
| Myanmar | 4840 (2650-10576) | 11.61 (6.27-25.94) | 1062 (652-1813) | 1.98 (1.21-3.33) | -2.82 (-5.12--0.47) |
| Namibia | 231 (40-786) | 19.37 (4.64-62.13) | 68 (36-193) | 3.43 (1.97-8.67) | -6.34 (-8.2--4.43) |
| Nauru | 0 (0-0) | 0.64 (0.34-0.89) | 0 (0-0) | 0.57 (0.24-0.83) | -0.26 (-0.65-0.14) |
| Nepal | 2285 (951-5723) | 12.17 (5.4-26.81) | 909 (613-1326) | 3.59 (2.46-5.04) | -4.14 (-4.28--4) |
| Netherlands | 6 (5-6) | 0.03 (0.03-0.04) | 14 (12-17) | 0.04 (0.03-0.05) | 1.2 (0.67-1.74) |
| New Zealand | 1 (1-1) | 0.03 (0.02-0.03) | 1 (0-1) | 0.01 (0.01-0.01) | -4.68 (-5.05--4.3) |
| Nicaragua | 58 (47-74) | 1.61 (1.37-1.93) | 101 (15-169) | 1.63 (0.26-2.7) | -4.24 (-5.35--3.13) |
| Niger | 19030 (8528-36622) | 180.59 (75.89-354.87) | 35184 (13152-62651) | 146.57 (50.9-278.66) | -0.5 (-0.82--0.18) |
| Nigeria | 160423 (87993-271785) | 158.57 (85.63-267.78) | 237966 (86012-484805) | 116.84 (41.05-245.94) | -1.42 (-1.79--1.05) |
| Niue | 0 (0-0) | 0.46 (0.24-0.75) | 0 (0-0) | 0.49 (0.24-0.75) | -0.74 (-1.06--0.43) |
| North Macedonia | 3 (1-23) | 0.17 (0.04-1.22) | 1 (0-9) | 0.07 (0.01-0.51) | -3.02 (-3.27--2.78) |
| Northern Mariana Islands | 0 (0-0) | 0.31 (0.09-0.42) | 0 (0-0) | 0.29 (0.07-0.4) | -0.13 (-0.6-0.33) |
| Norway | 2 (1-2) | 0.02 (0.02-0.02) | 6 (5-7) | 0.05 (0.04-0.06) | 3.26 (2.83-3.69) |
| Oman | 30 (14-81) | 2.52 (1.42-4.91) | 14 (9-22) | 0.9 (0.58-1.44) | -4.61 (-6--3.21) |
| Pakistan | 14485 (2397-72172) | 13.25 (2.36-72.38) | 10775 (2576-27389) | 4.92 (1.44-12.16) | -3.17 (-4.28--2.05) |
| Palau | 0 (0-0) | 0.44 (0.11-0.65) | 0 (0-0) | 0.33 (0.08-0.47) | -0.68 (-0.81--0.56) |
| Palestine | 13 (3-83) | 0.71 (0.24-3.27) | 3 (1-13) | 0.1 (0.05-0.27) | -5.53 (-5.96--5.1) |
| Panama | 20 (16-28) | 0.91 (0.74-1.23) | 10 (8-13) | 0.24 (0.18-0.32) | -3.59 (-4.07--3.11) |
| Papua New Guinea | 2270 (388-7296) | 62.73 (10.5-213.66) | 2213 (904-4231) | 24.32 (9.72-47.75) | -2.5 (-3.31--1.68) |
| Paraguay | 107 (79-149) | 4.18 (3.27-5.35) | 90 (60-132) | 1.49 (1.03-2.17) | -1.84 (-2.47--1.21) |
| Peru | 1382 (437-3331) | 7.04 (2.45-16.61) | 185 (123-314) | 0.53 (0.35-0.89) | -8.62 (-9.48--7.74) |
| Philippines | 2425 (1783-4804) | 3.88 (2.9-8.43) | 2680 (2020-3274) | 2.52 (1.91-3.06) | -0.44 (-0.77--0.11) |
| Poland | 36 (30-41) | 0.11 (0.09-0.12) | 12 (9-14) | 0.02 (0.01-0.02) | -6.83 (-7.39--6.27) |
| Portugal | 36 (26-78) | 0.31 (0.22-0.8) | 14 (11-21) | 0.06 (0.05-0.15) | -5.62 (-6.21--5.02) |
| Puerto Rico | 15 (9-23) | 0.53 (0.31-0.85) | 6 (4-7) | 0.1 (0.08-0.12) | -3.5 (-4.43--2.56) |
| Qatar | 0 (0-0) | 0.04 (0.02-0.05) | 0 (0-0) | 0.01 (0-0.01) | -4.5 (-4.9--4.1) |
| Republic of Korea | 32 (29-38) | 0.1 (0.09-0.12) | 81 (20-103) | 0.09 (0.02-0.11) | -0.71 (-1.45-0.03) |
| Republic of Moldova | 4 (2-5) | 0.09 (0.05-0.13) | 1 (1-2) | 0.02 (0.01-0.03) | -4.29 (-4.58--4) |
| Romania | 97 (27-621) | 0.43 (0.11-2.88) | 11 (8-14) | 0.04 (0.03-0.06) | -8.48 (-9.11--7.85) |
| Russian Federation | 209 (163-251) | 0.14 (0.12-0.17) | 136 (118-152) | 0.08 (0.07-0.09) | -2.28 (-2.53--2.03) |
| Rwanda | 9900 (5196-19222) | 122.45 (64.08-238.92) | 3288 (1922-5611) | 27.07 (15.83-46.72) | -6.57 (-7.51--5.63) |
| Saint Kitts and Nevis | 0 (0-0) | 0.2 (0.12-0.3) | 0 (0-0) | 0.13 (0.1-0.16) | -1.12 (-2.08--0.14) |
| Saint Lucia | 0 (0-0) | 0.35 (0.25-0.47) | 1 (1-1) | 0.5 (0.4-0.61) | 0.8 (-0.14-1.76) |
| Saint Vincent and the Grenadines | 1 (1-1) | 0.85 (0.73-0.99) | 0 (0-1) | 0.38 (0.32-0.45) | -0.08 (-1.01-0.85) |
| Samoa | 1 (0-1) | 0.65 (0.38-0.92) | 1 (0-1) | 0.52 (0.21-0.74) | -0.58 (-0.64--0.51) |
| San Marino | 0 (0-0) | 0.16 (0.01-0.24) | 0 (0-0) | 0.06 (0-0.1) | -2.74 (-3.26--2.22) |
| Sao Tome and Principe | 51 (16-117) | 44.75 (11.83-113.03) | 14 (8-20) | 6.96 (4.42-9.67) | -7.45 (-9.08--5.8) |
| Saudi Arabia | 175 (93-434) | 1.66 (1.01-2.95) | 60 (44-77) | 0.27 (0.2-0.35) | -6.54 (-7.17--5.9) |
| Senegal | 6342 (2366-14822) | 85.51 (31.29-200.35) | 5469 (2677-7922) | 44.69 (22.3-65.46) | -3.86 (-4.9--2.81) |
| Serbia | 28 (5-195) | 0.31 (0.05-2.29) | 7 (3-10) | 0.05 (0.02-0.08) | -6.59 (-7.31--5.87) |
| Seychelles | 0 (0-0) | 0.24 (0.13-0.47) | 0 (0-0) | 0.14 (0.05-0.39) | -0.95 (-1.16--0.74) |
| Sierra Leone | 10213 (4321-18207) | 216.56 (88-390.38) | 13442 (4689-25726) | 175.52 (59.24-339.68) | -1.16 (-1.64--0.69) |
| Singapore | 3 (3-3) | 0.11 (0.1-0.13) | 1 (0-1) | 0.01 (0-0.02) | -7.69 (-9.85--5.49) |
| Slovakia | 2 (1-2) | 0.03 (0.02-0.04) | 1 (0-1) | 0.01 (0-0.01) | -4.17 (-4.38--3.96) |
| Slovenia | 2 (1-14) | 0.11 (0.03-0.78) | 0 (0-1) | 0.01 (0-0.07) | -8.42 (-9.13--7.7) |
| Solomon Islands | 259 (31-850) | 131.63 (15.28-445.91) | 171 (98-305) | 43.71 (25.39-77.26) | -4.31 (-5.51--3.1) |
| Somalia | 10062 (6180-16530) | 113.73 (70.46-185.35) | 3385 (1778-6491) | 23.04 (13.36-43.11) | -6.35 (-7.12--5.57) |
| South Africa | 483 (296-951) | 1.48 (0.93-2.79) | 363 (307-562) | 0.72 (0.62-1.06) | -2.38 (-3.01--1.74) |
| South Sudan | 9182 (6797-13156) | 128.07 (92.94-187.77) | 8569 (4304-15759) | 77.31 (36.91-145.93) | -1.82 (-2.33--1.31) |
| Spain | 83 (49-289) | 0.2 (0.11-0.82) | 28 (18-66) | 0.04 (0.02-0.16) | -4.58 (-5.38--3.77) |
| Sri Lanka | 894 (516-1816) | 5.98 (3.48-12.12) | 221 (123-350) | 0.99 (0.55-1.56) | -6.49 (-7.82--5.14) |
| Sudan | 7674 (3988-14401) | 37.25 (19.12-65.31) | 4770 (1942-10289) | 11.69 (4.7-25.96) | -4.97 (-5.55--4.39) |
| Suriname | 48 (8-257) | 12.97 (2.3-69.12) | 6 (4-9) | 0.98 (0.61-1.44) | -8.5 (-10.32--6.63) |
| Sweden | 3 (2-3) | 0.02 (0.02-0.02) | 7 (6-9) | 0.03 (0.03-0.04) | 1.36 (0.94-1.78) |
| Switzerland | 2 (2-2) | 0.02 (0.02-0.02) | 4 (4-5) | 0.02 (0.02-0.03) | 1.67 (0.85-2.5) |
| Syrian Arab Republic | 68 (46-139) | 0.85 (0.59-1.28) | 32 (19-64) | 0.27 (0.17-0.51) | -4.15 (-4.58--3.71) |
| Taiwan (Province of China) | 12 (10-13) | 0.07 (0.07-0.08) | 30 (17-47) | 0.07 (0.04-0.11) | 2.69 (1.23-4.18) |
| Tajikistan | 333 (48-948) | 3.74 (0.62-10.77) | 18 (8-53) | 0.17 (0.08-0.47) | -10.57 (-11.76--9.37) |
| Thailand | 1884 (1009-3414) | 3.45 (1.89-6.25) | 233 (156-326) | 0.34 (0.23-0.48) | -6.01 (-6.48--5.55) |
| Timor-Leste | 101 (38-274) | 9.51 (3.52-25.91) | 11 (5-16) | 0.82 (0.41-1.21) | -7.9 (-12.07--3.52) |
| Togo | 4464 (2258-7679) | 118.49 (59.51-206.07) | 6109 (2276-11440) | 93.74 (35.09-183.05) | -0.35 (-0.83-0.13) |
| Tokelau | 0 (0-0) | 0.66 (0.34-0.95) | 0 (0-0) | 0.54 (0.25-0.77) | -1.17 (-1.38--0.97) |
| Tonga | 1 (0-2) | 1.87 (0.56-4.21) | 1 (1-2) | 1.51 (0.67-2.81) | -0.11 (-0.59-0.37) |
| Trinidad and Tobago | 4 (3-8) | 0.38 (0.25-0.71) | 9 (6-14) | 0.58 (0.36-0.86) | 1.93 (1.11-2.75) |
| Tunisia | 53 (28-210) | 0.79 (0.45-2.44) | 24 (15-45) | 0.2 (0.12-0.39) | -4.38 (-4.65--4.1) |
| Turkey | 645 (316-2928) | 1.34 (0.76-4.94) | 136 (106-174) | 0.16 (0.12-0.2) | -6.41 (-6.8--6.02) |
| Turkmenistan | 3 (1-14) | 0.08 (0.04-0.32) | 4 (1-27) | 0.07 (0.01-0.51) | -1.65 (-2.29--1) |
| Tuvalu | 0 (0-0) | 0.81 (0.45-1.13) | 0 (0-0) | 0.56 (0.19-0.84) | -0.88 (-0.98--0.78) |
| Uganda | 48816 (32322-73316) | 222.78 (140.95-340.79) | 46225 (15736-83320) | 96.43 (31.18-185.47) | -5.16 (-5.94--4.36) |
| Ukraine | 31 (25-38) | 0.06 (0.05-0.08) | 15 (11-19) | 0.04 (0.03-0.05) | -1.64 (-1.85--1.43) |
| United Arab Emirates | 1 (0-2) | 0.09 (0.05-0.16) | 1 (0-2) | 0.03 (0.02-0.06) | -2.2 (-2.59--1.81) |
| United Kingdom | 28 (26-30) | 0.04 (0.03-0.04) | 10 (9-11) | 0.01 (0.01-0.01) | -4.41 (-4.78--4.04) |
| United Republic of Tanzania | 35219 (18691-58723) | 98.12 (51.56-166.57) | 16566 (5952-33483) | 26.13 (9.84-54.36) | -5.72 (-6.46--4.97) |
| United States of America | 106 (96-115) | 0.04 (0.03-0.04) | 365 (327-395) | 0.08 (0.07-0.08) | 3.34 (2.83-3.87) |
| United States Virgin Islands | 0 (0-0) | 0.09 (0.05-0.13) | 0 (0-0) | 0.03 (0.01-0.04) | -3.34 (-3.54--3.15) |
| Uruguay | 15 (11-20) | 0.43 (0.31-0.57) | 7 (6-8) | 0.13 (0.11-0.16) | -3.38 (-3.84--2.91) |
| Uzbekistan | 31 (25-39) | 0.18 (0.14-0.22) | 33 (22-71) | 0.1 (0.07-0.21) | -1.66 (-1.92--1.4) |
| Vanuatu | 60 (23-127) | 64.09 (24.28-137.06) | 1 (0-1) | 0.51 (0.23-0.74) | -11.85 (-15.46--8.08) |
| Venezuela (Bolivarian Republic of) | 1254 (1028-1453) | 12.58 (9.81-15.12) | 1516 (1004-2262) | 5.54 (3.68-8.27) | -2.76 (-3.43--2.09) |
| Viet Nam | 1270 (557-3909) | 2.06 (0.89-6.45) | 338 (169-481) | 0.37 (0.19-0.52) | -6.29 (-6.9--5.67) |
| Yemen | 6393 (2002-14917) | 56.78 (17.53-138.67) | 9228 (1843-21752) | 38.03 (7.28-93.79) | -1.57 (-2.71--0.41) |
| Zambia | 9807 (6551-15609) | 110.82 (71.5-177.82) | 7007 (2038-15031) | 43.1 (12.52-96.44) | -4.44 (-5.15--3.72) |
| Zimbabwe | 2904 (1228-5787) | 25.01 (10.61-52.01) | 2066 (1091-3824) | 12.83 (7.36-22.73) | 0.48 (-0.82-1.79) |

**Table S4.** The number of DALYs cases and the age-standardized DALYs rate of neglected tropical diseases and malaria in 1990 and 2021, and its trends from 1990 to 2021 globally. Abbreviations: DALYs, disability-adjusted-life-years.

|  | Number of DALYs cases (95% UI) in 1990 | The age-standardized DALYs rate/100000 (95% UI) in 1990 | Number of DALYs cases (95% UI) in 2021 | The age-standardized DALYs rate/100000 (95% UI) in 2021 | EAPC (95% CI) |
| --- | --- | --- | --- | --- | --- |
| Global | 87421645 (59036484-141599541) | 1506.54 (1018.86-2423.16) | 71629913 (38735014-122926457) | 1020.27 (542.47-1756.51) | -1.66 (-1.94--1.38) |
| Sex |  |  |  |  |  |
| Female | 40600027 (27071519-66753649) | 1421.07 (955.67-2319.66) | 34484424 (18919364-57942089) | 1009.16 (541.15-1712.81) | -1.51 (-1.8--1.23) |
| Male | 46821618 (31720094-75346746) | 1594.02 (1092.3-2541.63) | 37145489 (19742580-64957948) | 1032.16 (540.1-1814.79) | -1.8 (-2.08--1.53) |
| Age |  |  |  |  |  |
| <5 years | 50682165 (30547921-88779156) | 8175.36 (4927.58-14320.65) | 40912262 (18772471-72969299) | 6216.05 (2852.21-11086.67) | -1.4 (-1.78--1.01) |
| 5-9 years | 7944471 (5437120-13126152) | 1361.45 (931.76-2249.44) | 4569048 (2875733-7283114) | 665.02 (418.56-1060.05) | -2.25 (-2.47--2.03) |
| 10-14 years | 4744638 (3478695-7149679) | 885.72 (649.39-1334.69) | 3012868 (2038661-4739053) | 451.95 (305.81-710.89) | -2.19 (-2.35--2.03) |
| 15-19 years | 3715281 (2701217-5527176) | 715.27 (520.04-1064.1) | 2884190 (1859176-4619745) | 462.22 (297.95-740.37) | -1.74 (-1.86--1.62) |
| 20-24 years | 3251865 (2349817-4789176) | 660.83 (477.52-973.24) | 2725598 (1754829-4363742) | 456.43 (293.86-730.75) | -1.66 (-1.89--1.43) |
| 25-29 years | 2713535 (1963878-3869570) | 613.06 (443.69-874.24) | 2304865 (1519716-3573998) | 391.76 (258.3-607.47) | -1.81 (-2.04--1.57) |
| 30-34 years | 2312112 (1686272-3212485) | 599.89 (437.51-833.5) | 2080960 (1373954-3227989) | 344.26 (227.3-534.01) | -1.96 (-2.11--1.82) |
| 35-39 years | 2145875 (1568773-3070922) | 609.2 (445.36-871.82) | 1979983 (1294389-3170874) | 353.02 (230.78-565.35) | -1.93 (-2.05--1.82) |
| 40-44 years | 1810611 (1364734-2554301) | 632.02 (476.38-891.61) | 1844939 (1202441-3007036) | 368.8 (240.37-601.11) | -2.05 (-2.2--1.89) |
| 45-49 years | 1610714 (1222033-2264980) | 693.69 (526.29-975.46) | 1721220 (1099157-2804654) | 363.51 (232.13-592.32) | -2.33 (-2.46--2.2) |
| 50-54 years | 1532086 (1130243-2228929) | 720.74 (531.7-1048.55) | 1668066 (1033139-2859901) | 374.91 (232.21-642.79) | -2.49 (-2.62--2.35) |
| 55-59 years | 1415133 (1044339-2088758) | 764.11 (563.9-1127.84) | 1595483 (997357-2713598) | 403.18 (252.03-685.72) | -2.38 (-2.57--2.19) |
| 60-64 years | 1267359 (913646-1969213) | 789.09 (568.86-1226.09) | 1402164 (848672-2467248) | 438.11 (265.17-770.9) | -2.39 (-2.66--2.11) |
| 65-69 years | 968860 (709305-1446963) | 783.81 (573.83-1170.59) | 1121931 (700837-1910972) | 406.73 (254.07-692.78) | -2.32 (-2.61--2.03) |
| 70-74 years | 738443 (540963-1122021) | 872.23 (638.97-1325.31) | 956450 (587996-1618307) | 464.66 (285.66-786.2) | -1.97 (-2.11--1.84) |
| 75-79 years | 342951 (266004-443766) | 557.14 (432.14-720.92) | 466057 (321734-701310) | 353.38 (243.95-531.76) | -2.29 (-2.63--1.94) |
| 80-84 years | 149739 (118029-185845) | 423.28 (333.64-525.34) | 230683 (179225-301667) | 263.39 (204.63-344.44) | -2.08 (-2.42--1.73) |
| 85-89 years | 56389 (45892-68755) | 373.16 (303.7-455) | 100958 (80607-124767) | 220.81 (176.3-272.88) | -1.87 (-2.07--1.66) |
| 90-94 years | 15353 (12197-19062) | 358.29 (284.63-444.83) | 37796 (28599-49203) | 211.28 (159.87-275.04) | -1.59 (-1.67--1.51) |
| 95+ years | 4065 (3073-5126) | 399.25 (301.85-503.54) | 14392 (10367-19332) | 264.06 (190.21-354.69) | -0.93 (-1.11--0.75) |
| SDI region | |  |  |  |  |
| High-middle SDI | 1733826 (1236792-2381383) | 165.41 (118.53-225.86) | 1042704 (755397-1437907) | 74.57 (54.92-100.62) | -2.46 (-2.67--2.24) |
| High SDI | 277167 (198860-371435) | 30.82 (22.32-41.28) | 298096 (212845-403832) | 22.21 (15.79-30.09) | -0.93 (-1--0.86) |
| Low-middle SDI | 23836816 (15749352-42401086) | 1770.33 (1203.04-3042.22) | 16949582 (9990131-28848944) | 898.01 (529.14-1530.22) | -2.54 (-2.81--2.27) |
| Low SDI | 49204865 (32261033-77330184) | 7031.82 (4746.86-10882.03) | 45665221 (22093748-81355171) | 3420.13 (1661.76-6177.45) | -2.7 (-2.92--2.47) |
| Middle SDI | 12324377 (8825751-19842462) | 705.62 (517.68-1102.87) | 7644826 (5513179-10649711) | 351.51 (247.06-488.97) | -2.41 (-2.59--2.24) |
| GBD region | |  |  |  |  |
| Advanced Health System | 560909 (400789-841018) | 42.32 (29.68-67.66) | 477040 (345821-648164) | 26.26 (19.32-34.98) | -1.58 (-1.64--1.52) |
| Africa | 59250334 (38378081-90652290) | 6897.3 (4580.6-10387.61) | 59691029 (29181579-105904842) | 3687.93 (1808.2-6658.53) | -2.55 (-2.88--2.23) |
| African Region | 57411407 (36636850-88158497) | 8157.37 (5353.29-12308.15) | 58800552 (28587016-104587091) | 4330.21 (2105.48-7860.93) | -2.54 (-2.87--2.22) |
| America | 2153605 (1741020-2713357) | 305.99 (249.52-381.11) | 1556218 (1219681-2003089) | 145.58 (112.24-188.69) | -2.51 (-2.65--2.36) |
| Andean Latin America | 267057 (171671-432442) | 741.6 (505.62-1127.7) | 162786 (117520-216492) | 250.76 (182.06-332.19) | -3.66 (-3.93--3.39) |
| Asia | 25571255 (16306896-47322495) | 766.1 (495.82-1407.24) | 10094139 (7392576-14401328) | 231.7 (166.84-336.86) | -3.63 (-3.85--3.41) |
| Australasia | 1272 (838-1898) | 6.6 (4.25-10.09) | 1467 (948-2268) | 4.98 (3.18-8.01) | -0.74 (-0.92--0.56) |
| Basic Health System | 9103476 (6703256-13332003) | 406 (304.18-577.25) | 4755010 (3710733-6002431) | 155.58 (121.77-195.89) | -3.06 (-3.22--2.89) |
| Caribbean | 171098 (126097-249617) | 474.94 (352.73-682.8) | 119732 (75416-224295) | 255.86 (160.24-481.73) | -3.66 (-4.4--2.91) |
| Central Africa | 12092112 (7804582-17839642) | 12149.85 (8273.87-17441.05) | 11182025 (6001057-19367676) | 5607.42 (3108.74-9720.92) | -3.09 (-3.47--2.7) |
| Central Asia | 105738 (68584-159862) | 137.56 (92.79-198.83) | 72537 (51180-100653) | 75.21 (53.2-103.93) | -2.1 (-2.22--1.99) |
| Central Europe | 87364 (58281-165649) | 69.14 (45.51-141.28) | 50614 (34418-72781) | 36.35 (24.83-50.58) | -2.23 (-2.31--2.14) |
| Central Latin America | 534207 (438875-645686) | 344.87 (283-416.87) | 419308 (326908-531551) | 166.62 (130.72-210.52) | -2.28 (-2.52--2.03) |
| Central Sub-Saharan Africa | 9930169 (6442856-14563610) | 12217.76 (8440.22-17211.58) | 8486323 (4707786-14132194) | 5167.37 (2958.96-8758.09) | -3.42 (-3.83--3.02) |
| Commonwealth High Income | 12497 (8411-18568) | 12.4 (8.25-18.52) | 14436 (8623-23545) | 10.72 (6.54-17.43) | -0.14 (-0.49-0.22) |
| Commonwealth Low Income | 16480628 (10576414-27001801) | 5633.06 (3644.2-9393) | 10205357 (4526778-17777218) | 2366.84 (1061-4161.05) | -4.26 (-4.73--3.79) |
| Commonwealth Middle Income | 32727854 (20951805-58088159) | 2370.46 (1564.28-4066.72) | 28195057 (14355514-51916846) | 1384.86 (704.28-2558.39) | -1.85 (-2.05--1.65) |
| East Asia | 3032479 (1963236-4387637) | 256.7 (169.67-369.48) | 1260884 (818872-1923685) | 73.03 (48.29-112.57) | -3.84 (-4.19--3.49) |
| East Asia & Pacific - WB | 7316007 (5177487-11027526) | 394.75 (279.49-589.22) | 3228198 (2478508-4087323) | 148.08 (116.04-182.7) | -2.92 (-3.07--2.76) |
| Eastern Africa | 14335433 (10337817-20934537) | 5896.44 (4411.33-8373.03) | 9617215 (4833964-16093815) | 2093.06 (1106.89-3509.05) | -4.5 (-4.97--4.02) |
| Eastern Europe | 173968 (125628-229377) | 72.87 (53.05-95.61) | 128890 (88206-175844) | 52.25 (37.53-70.08) | -1.25 (-1.34--1.16) |
| Eastern Mediterranean Region | 4034917 (2288022-8926598) | 945.77 (544.95-2076.15) | 2875385 (1618632-5159655) | 373.86 (213.02-670.49) | -3.39 (-3.79--2.98) |
| Eastern Sub-Saharan Africa | 20532064 (14255284-30817646) | 7590.25 (5475.84-11175.33) | 13637096 (6554872-23783903) | 2721.72 (1350.65-4749.97) | -4.44 (-4.88--4.01) |
| Europe | 397788 (276601-670317) | 50.12 (34.45-91.73) | 256137 (184112-339592) | 27.1 (19.7-35.95) | -2.12 (-2.19--2.06) |
| Europe & Central Asia - WB | 484465 (340549-761538) | 59.08 (41.21-97.39) | 314056 (226143-417940) | 32.79 (23.78-43.49) | -2.05 (-2.14--1.97) |
| European Region | 487170 (342395-766411) | 58.86 (41.06-97.09) | 317288 (228625-422497) | 32.62 (23.65-43.33) | -2.06 (-2.15--1.97) |
| High-income Asia Pacific | 47758 (34362-64376) | 26.25 (18.77-35.03) | 59408 (40451-84466) | 25.25 (16.85-35.65) | 0.14 (-0.03-0.32) |
| High-income North America | 68708 (44056-102839) | 21.8 (14.19-32.49) | 120205 (79514-180112) | 24.22 (16.46-35.02) | 0.47 (0.03-0.91) |
| Latin America & Caribbean - WB | 2088702 (1686241-2644359) | 512.64 (424.75-626.45) | 1438724 (1124338-1848006) | 215.23 (166.82-277.04) | -2.89 (-3.06--2.72) |
| Limited Health System | 55319140 (36193699-93789059) | 2883.7 (1936.69-4765.24) | 43670626 (22035181-78188951) | 1502.35 (761.07-2696.94) | -2.53 (-2.8--2.27) |
| Middle East & North Africa - WB | 1134743 (671976-2049033) | 451.47 (284.68-783.89) | 978083 (570230-1601118) | 209 (123.22-340.46) | -2.88 (-3.3--2.46) |
| Minimal Health System | 22393525 (14317042-34668499) | 11190.26 (7444.18-16990.75) | 22697753 (11863292-39166507) | 5448.87 (2868.02-9613.6) | -2.6 (-2.83--2.38) |
| North Africa and Middle East | 1873972 (1114705-3711286) | 531.67 (335.11-952.56) | 1483826 (898738-2401774) | 239.96 (146.82-387.55) | -3.14 (-3.53--2.76) |
| North America | 68750 (44084-102903) | 21.82 (14.2-32.51) | 120232 (79530-180167) | 24.22 (16.46-35.03) | 0.47 (0.03-0.91) |
| Northern Africa | 434497 (288520-762244) | 396.13 (273.97-621.62) | 213236 (155919-289215) | 104.77 (77.35-141.4) | -4.62 (-4.93--4.3) |
| Oceania | 232177 (99976-560138) | 3595.65 (1559.45-8916.27) | 189817 (105112-318115) | 1375.48 (763.19-2333.13) | -2.92 (-3.5--2.33) |
| Region of the Americas | 2153605 (1741020-2713357) | 305.99 (249.52-381.11) | 1556218 (1219681-2003089) | 145.58 (112.24-188.69) | -2.51 (-2.65--2.36) |
| South-East Asia Region | 18790122 (11797886-34064484) | 1303.27 (843.98-2287.45) | 6020356 (4629162-8631774) | 311.39 (237.2-450.73) | -4.29 (-4.61--3.98) |
| South Asia | 17302057 (10230090-33868111) | 1388.66 (860.31-2610.13) | 5899887 (4019140-9447919) | 334.54 (227.46-538.32) | -4.33 (-4.63--4.04) |
| South Asia - WB | 17453592 (10325673-34044633) | 1368.44 (848.04-2565.41) | 6021550 (4122704-9577834) | 330.45 (226.15-527.51) | -4.33 (-4.62--4.04) |
| Southeast Asia | 4109639 (2873242-6809999) | 860.34 (605.6-1413.77) | 1755385 (1367265-2175938) | 270 (210.25-333.88) | -3.55 (-3.73--3.36) |
| Southern Africa | 6906839 (4564187-10842014) | 5620.52 (3750.67-8717.92) | 5059615 (2255227-9794864) | 2504.17 (1117.42-4915.27) | -3.61 (-3.99--3.24) |
| Southern Latin America | 77096 (59684-99318) | 162.57 (126.02-208.13) | 46038 (34405-61068) | 60.06 (44.83-79.22) | -3.03 (-3.16--2.9) |
| Southern Sub-Saharan Africa | 503846 (314887-798788) | 866.16 (566.79-1331.97) | 339829 (241972-505115) | 427.33 (305.49-630.05) | -0.88 (-1.52--0.23) |
| Sub-Saharan Africa - WB | 58828932 (38019454-90075268) | 8298.86 (5474.74-12496.98) | 59498677 (29003013-105733253) | 4318.6 (2106.72-7840.45) | -2.6 (-2.91--2.29) |
| Tropical Latin America | 1045091 (842686-1312287) | 769.32 (647.11-924.09) | 694948 (485914-992157) | 299 (204.41-437.27) | -2.92 (-3.13--2.71) |
| Western Africa | 25481454 (14929916-41439749) | 10628.52 (6451.6-17033.49) | 33618937 (15757647-60070609) | 6512.54 (3030.76-12067.72) | -1.8 (-2.14--1.47) |
| Western Europe | 52076 (37270-77704) | 14.44 (10.19-23.04) | 41328 (27414-57803) | 8.86 (6.1-12.36) | -1.57 (-1.62--1.52) |
| Western Pacific Region | 4478734 (3102580-6324333) | 294.1 (205.5-411.19) | 2021583 (1462425-2772138) | 102.65 (76.29-136.56) | -3.25 (-3.47--3.03) |
| Western Sub-Saharan Africa | 27273811 (16035045-44122226) | 10288.4 (6274.19-16404.31) | 36659606 (17198370-66398958) | 6344.96 (2965.31-11843.38) | -1.82 (-2.16--1.48) |
| World Bank High Income | 307408 (224741-416808) | 30.12 (21.73-41.61) | 321821 (228117-443042) | 21.75 (15.81-29.46) | -1.01 (-1.07--0.95) |
| World Bank Low Income | 34541685 (23306786-52462636) | 7352.6 (5145.43-10949.16) | 30712579 (15516847-52918824) | 3509.36 (1788.74-6118.45) | -2.82 (-3.06--2.58) |
| World Bank Lower Middle Income | 46367144 (29508959-81623964) | 2046.35 (1344.11-3529.65) | 37416061 (19985686-66207976) | 1162.82 (620.11-2065.54) | -2.13 (-2.38--1.89) |
| World Bank Upper Middle Income | 6175371 (4567332-8128847) | 309.01 (232.29-403.74) | 3217992 (2439910-4216990) | 116.95 (88.98-153.87) | -3.09 (-3.28--2.9) |
| Country |  |  |  |  |  |
| Afghanistan | 66673 (25806-300126) | 625.84 (260.13-2556.23) | 95340 (68034-132066) | 320.87 (231.1-445.51) | -2.05 (-3.17--0.92) |
| Albania | 2764 (1237-12128) | 79.13 (36.74-328.41) | 664 (359-1859) | 28.44 (14.54-89.02) | -4.12 (-4.75--3.49) |
| Algeria | 29982 (19476-60533) | 122.29 (81.93-210.8) | 29795 (20136-42687) | 68.25 (46.37-97.36) | -1.78 (-1.89--1.67) |
| American Samoa | 117 (77-174) | 284.19 (191.72-418.13) | 73 (47-117) | 149.1 (96.97-237.2) | -4.58 (-5.87--3.27) |
| Andorra | 3 (2-5) | 7.55 (4.89-10.76) | 3 (2-5) | 4.81 (3.04-7.19) | -1.42 (-1.61--1.23) |
| Angola | 851441 (517878-1367359) | 6425.71 (4152.1-9755.28) | 1476596 (714747-2809236) | 3847.64 (1833.77-7555.13) | -3.92 (-4.8--3.02) |
| Antigua and Barbuda | 62 (32-117) | 101.54 (52.99-192.03) | 74 (37-154) | 82.19 (41.41-165.8) | -0.76 (-0.85--0.67) |
| Argentina | 62970 (48610-82077) | 195.42 (151.67-252.38) | 32052 (23634-42505) | 64.43 (47.51-85.4) | -3.42 (-3.54--3.29) |
| Armenia | 2948 (1975-4086) | 87.72 (59.11-122.62) | 1703 (1023-2652) | 56.32 (34.67-95.38) | -1.74 (-1.84--1.64) |
| Australia | 1018 (654-1539) | 6.31 (3.99-9.81) | 1225 (803-1889) | 4.9 (3.14-7.85) | -0.59 (-0.8--0.38) |
| Austria | 2355 (1258-4059) | 27.28 (14.79-49.52) | 2228 (1093-4494) | 18.12 (9.19-33.98) | -1.46 (-1.57--1.36) |
| Azerbaijan | 5967 (4000-8365) | 76.43 (51.27-106.82) | 4814 (3076-7632) | 49.37 (31.27-80.86) | -1.91 (-2.1--1.73) |
| Bahamas | 257 (161-398) | 111.98 (70.25-172.7) | 315 (183-512) | 81.09 (48.17-130) | -1.17 (-1.24--1.1) |
| Bahrain | 170 (107-258) | 31.53 (20.41-47.5) | 232 (149-354) | 17.28 (11.3-26.3) | -2.29 (-2.41--2.16) |
| Bangladesh | 1596419 (570058-3907795) | 1292.37 (474.05-3350.21) | 239719 (166795-328153) | 146.13 (102.37-199.6) | -6.75 (-7.47--6.02) |
| Barbados | 280 (178-427) | 109.93 (70.72-166.03) | 316 (192-509) | 96.22 (59.99-155.01) | 0 (-0.46-0.46) |
| Belarus | 6400 (4061-9421) | 58.47 (37.66-84.97) | 3593 (2207-5731) | 33.23 (21.66-49.74) | -2.05 (-2.25--1.85) |
| Belgium | 776 (544-1091) | 8.88 (5.86-13.1) | 542 (354-768) | 5.59 (3.48-8.22) | -1.5 (-1.62--1.39) |
| Belize | 403 (275-733) | 215.42 (149.07-351.25) | 410 (280-567) | 102.3 (69.42-142.59) | -2.63 (-2.92--2.33) |
| Benin | 547258 (346923-802514) | 7966.37 (5306.43-11213.86) | 1229670 (667598-2033627) | 7503.18 (3905.21-12977.34) | -0.1 (-0.58-0.39) |
| Bermuda | 50 (31-75) | 81.74 (51.47-121.46) | 34 (17-59) | 39.19 (22.01-62.37) | -2.54 (-2.65--2.43) |
| Bhutan | 53592 (11279-137349) | 6322.21 (1383.7-16756.84) | 1174 (818-1616) | 170.92 (119.3-234.08) | -12.18 (-13.28--11.07) |
| Bolivia (Plurinational State of) | 52886 (36979-80742) | 1053.77 (769.55-1437.25) | 41130 (30140-54562) | 383.94 (284.21-503.68) | -3.14 (-3.27--3.02) |
| Bosnia and Herzegovina | 2891 (1753-6261) | 66.26 (40.19-147.09) | 1454 (860-2339) | 37.08 (23.5-56.36) | -2.22 (-2.43--2.01) |
| Botswana | 8277 (5747-12286) | 661.82 (472.12-957.46) | 7650 (5268-11570) | 328.94 (231.62-489.17) | -2.59 (-2.87--2.32) |
| Brazil | 1035864 (832735-1302879) | 783.38 (658.39-943.48) | 685043 (477841-978878) | 303.9 (206.79-445.33) | -2.94 (-3.15--2.73) |
| Brunei Darussalam | 125 (77-205) | 55.17 (35.41-88.04) | 183 (112-299) | 42.19 (26.71-66.13) | -1.1 (-1.31--0.9) |
| Bulgaria | 4402 (2045-18296) | 58.86 (25.47-263.44) | 1550 (1110-2114) | 25.57 (17.55-35.75) | -2.92 (-3.13--2.71) |
| Burkina Faso | 1791035 (1050542-2908537) | 13512.23 (8174.58-22128.13) | 2703649 (1364142-4523398) | 9431.99 (4696.68-16231.3) | -1.37 (-1.9--0.83) |
| Burundi | 1248360 (677302-1874314) | 13759.82 (7758.03-20790.26) | 1165981 (583309-2037715) | 6578.74 (3382.85-12054.18) | -4.3 (-5.09--3.51) |
| Cabo Verde | 1349 (794-2478) | 359.24 (215.41-658.39) | 1462 (715-3475) | 276.66 (134.4-638.54) | -0.98 (-1.16--0.81) |
| Cambodia | 146063 (93759-251236) | 1171.92 (759.97-1951.29) | 19928 (14440-26999) | 120 (87.75-160.88) | -6.26 (-7.26--5.26) |
| Cameroon | 1231670 (789354-1840834) | 9150.3 (6008.29-13469.77) | 2181508 (1023720-3935462) | 6509.59 (3040.07-12099.01) | -1.95 (-2.57--1.33) |
| Canada | 976 (592-1619) | 3.65 (2.25-5.89) | 1368 (922-2008) | 3.51 (2.26-5.42) | -0.11 (-0.27-0.06) |
| Central African Republic | 320318 (224620-470202) | 9616.63 (7061.25-13227.16) | 432792 (206902-874687) | 7076.45 (3517.17-14113.21) | -1.08 (-1.56--0.59) |
| Chad | 529486 (316237-906713) | 6072.78 (3835.84-9994.89) | 823357 (378120-1512419) | 3604.12 (1741.33-6532.24) | -2.19 (-2.43--1.95) |
| Chile | 12106 (8452-16978) | 103.11 (72.06-143.21) | 12100 (7741-18945) | 52.27 (33.64-81.48) | -1.95 (-2.07--1.83) |
| China | 2999423 (1942078-4348872) | 262.98 (173.14-379.63) | 1245310 (806610-1909068) | 74.6 (49.1-115.59) | -3.84 (-4.2--3.49) |
| Colombia | 114690 (78975-169059) | 336.45 (236.76-485.42) | 59924 (44656-78330) | 122.95 (91.27-159.8) | -3.51 (-4--3.03) |
| Comoros | 15262 (3048-61266) | 3036.61 (650.58-12138.72) | 3971 (2423-8998) | 529 (322.2-1195.29) | -3.16 (-5.62--0.64) |
| Congo | 252183 (161560-370483) | 9439.31 (6147.83-13576.23) | 186292 (90139-315249) | 3488.87 (1686.48-6052) | -4.23 (-4.72--3.73) |
| Cook Islands | 516 (470-568) | 2428.25 (2210.5-2671.71) | 8 (5-11) | 44.14 (30.21-63.35) | -3.44 (-5.64--1.2) |
| Costa Rica | 4304 (2838-6411) | 157.64 (104.59-232.83) | 5717 (3414-8936) | 115.53 (68.69-179.15) | -0.59 (-1.01--0.16) |
| Croatia | 3836 (2208-6433) | 70.54 (42.16-114.78) | 2199 (1155-3827) | 39.46 (22.79-63.96) | -1.96 (-2.13--1.8) |
| Cuba | 21873 (13363-34852) | 209.01 (127.82-332.9) | 7678 (4564-12526) | 78.5 (46.87-127.33) | -3.81 (-4.19--3.43) |
| Cyprus | 98 (41-385) | 14.05 (6.07-53.74) | 66 (36-162) | 5.88 (3.03-15.07) | -3.34 (-3.63--3.05) |
| Czechia | 2209 (1435-3367) | 23.91 (15.32-37.19) | 1217 (787-1871) | 13.3 (8.41-20.45) | -1.83 (-1.98--1.68) |
| C么te d'Ivoire | 1872977 (1127514-2874743) | 12228.92 (7683.66-18514.33) | 2057992 (981426-3507203) | 6728.82 (3162.27-11894.56) | -2.23 (-2.9--1.55) |
| Democratic People's Republic of Korea | 16039 (10907-22857) | 78.59 (53.47-112.43) | 9439 (6798-12860) | 36.79 (26.27-50.69) | -2.74 (-2.87--2.6) |
| Democratic Republic of the Congo | 8367033 (5550629-12105532) | 14242.18 (9817.28-19878.1) | 6261131 (3653432-10026851) | 5706.58 (3358.87-9061.57) | -3.34 (-3.72--2.95) |
| Denmark | 322 (207-482) | 7.94 (4.94-12.29) | 265 (179-378) | 5.28 (3.34-7.74) | -1.51 (-1.61--1.41) |
| Djibouti | 13338 (8095-21206) | 2677.15 (1596.23-4443.13) | 13040 (8247-18671) | 1017.76 (641.4-1450.53) | -3.78 (-6.5--0.99) |
| Dominica | 98 (68-136) | 146.34 (99.18-205.9) | 70 (44-103) | 101.07 (66.19-146.1) | -1.25 (-1.34--1.17) |
| Dominican Republic | 25862 (18429-35332) | 395.5 (287.19-533.72) | 24111 (16213-36706) | 217.68 (147.18-326.03) | -3.17 (-3.83--2.51) |
| Ecuador | 66710 (45991-97156) | 674.57 (476.69-948.15) | 32678 (22558-45586) | 184.24 (127.21-256.94) | -4.6 (-5.42--3.78) |
| Egypt | 331235 (209251-608764) | 695.46 (471.6-1107.87) | 103028 (72083-148053) | 110.26 (77.79-155.45) | -6.63 (-7.06--6.19) |
| El Salvador | 13036 (9781-17483) | 260.51 (192.49-353.76) | 7599 (5205-10740) | 120 (82.05-169.41) | -2.41 (-2.52--2.29) |
| Equatorial Guinea | 55085 (31050-93201) | 9117.86 (5478.16-15010.07) | 72568 (35662-130430) | 4854.26 (2379.24-9509.27) | -3.19 (-3.57--2.81) |
| Eritrea | 66996 (44662-102467) | 1748.61 (1216.62-2653.1) | 31460 (24225-41044) | 494.68 (384.66-644.76) | -3.31 (-3.89--2.72) |
| Estonia | 930 (568-1401) | 54.7 (34.12-80.07) | 643 (345-1127) | 38.42 (22.28-63.09) | -1.22 (-1.4--1.05) |
| Eswatini | 6175 (3757-12288) | 757.94 (479.81-1462.12) | 2855 (2144-3675) | 271.29 (206.91-348.69) | -3.42 (-3.78--3.07) |
| Ethiopia | 2055601 (1595485-2572597) | 3565.31 (2885.27-4365.02) | 1147085 (736244-1875613) | 989.91 (695.2-1466.77) | -3.64 (-4.45--2.81) |
| Fiji | 4710 (3349-6306) | 659.33 (475.04-875.18) | 3047 (2175-4150) | 334.73 (239.27-454.23) | -2.94 (-3.68--2.19) |
| Finland | 318 (197-494) | 7.62 (4.69-11.95) | 201 (127-299) | 4.68 (2.89-7.01) | -1.76 (-1.84--1.68) |
| France | 6187 (4200-10656) | 12.05 (7.94-21.48) | 3426 (2357-4861) | 5.97 (3.9-8.71) | -2.47 (-2.66--2.28) |
| Gabon | 84109 (50881-127428) | 7318.02 (4580-11064.97) | 56945 (26380-113176) | 3177.78 (1464.5-6538.1) | -2.69 (-3--2.38) |
| Gambia | 62931 (29860-110120) | 5364.59 (2466.18-9392.25) | 40636 (28544-57231) | 1858.42 (1296.03-2714) | -1.72 (-3.05--0.37) |
| Georgia | 5272 (3492-9221) | 95.1 (62.43-172.13) | 3845 (2467-7518) | 101.18 (64.2-232.08) | 0.42 (0.18-0.66) |
| Germany | 4836 (3085-7491) | 7.6 (4.72-12.01) | 3380 (2303-4992) | 4.71 (2.95-7.12) | -1.44 (-1.69--1.2) |
| Ghana | 1513233 (900836-2351721) | 7566.25 (4792.01-11627.93) | 1252009 (711857-2040612) | 3435.49 (1919.88-5829.85) | -2.17 (-2.93--1.4) |
| Greece | 3361 (1683-6698) | 29.96 (14.8-71.7) | 2794 (1303-5241) | 21.08 (9.52-52.22) | -1 (-1.13--0.86) |
| Greenland | 6 (4-9) | 11.71 (8.39-16.11) | 5 (3-6) | 8.89 (5.7-12.43) | -0.74 (-0.84--0.63) |
| Grenada | 162 (115-221) | 191.8 (132.44-263.27) | 96 (62-135) | 93.08 (60.76-129.43) | -2.32 (-2.53--2.12) |
| Guam | 77 (51-115) | 57.78 (38.76-83.72) | 71 (47-101) | 44.94 (29.38-65.11) | -0.85 (-0.99--0.71) |
| Guatemala | 81774 (68065-99618) | 894.39 (730.53-1107.9) | 32563 (23418-43697) | 220.82 (157.49-292.13) | -4.68 (-5.07--4.28) |
| Guinea | 781348 (470818-1407887) | 8348.89 (5299.22-14444.74) | 1087673 (534636-1901372) | 6161.73 (3076.22-11132.18) | -0.53 (-0.96--0.09) |
| Guinea-Bissau | 122311 (76980-212542) | 8358.84 (5406.94-13927.83) | 54120 (21221-105375) | 2296.46 (957.55-4544.59) | -5.38 (-6.04--4.71) |
| Guyana | 19470 (11682-35325) | 2540.84 (1557.51-4535.24) | 7107 (4921-9679) | 922.95 (640.88-1248.83) | -4.55 (-5.47--3.61) |
| Haiti | 83996 (58074-127214) | 1274.9 (880.69-1895.04) | 66212 (29029-160439) | 507.7 (230.29-1221.56) | -5.23 (-6.31--4.13) |
| Honduras | 20044 (15137-27194) | 419 (319.79-572.74) | 22422 (15807-31498) | 239.77 (168.97-332.21) | -1.61 (-1.84--1.38) |
| Hungary | 7193 (4385-10864) | 63.16 (40.21-91.18) | 4289 (2352-7267) | 35.42 (20.5-55.19) | -1.97 (-2.05--1.89) |
| Iceland | 15 (9-23) | 6.24 (3.88-9.6) | 14 (9-21) | 4.31 (2.64-6.77) | -1.31 (-1.45--1.17) |
| India | 13911399 (8949009-25060201) | 1449.61 (965.87-2469.37) | 4456938 (3108487-7021067) | 335.81 (231.05-535.12) | -4.33 (-4.69--3.98) |
| Indonesia | 1731436 (1100583-3534060) | 896.16 (574.52-1850.91) | 963904 (693032-1241602) | 387.71 (279.39-505.17) | -2.51 (-2.74--2.27) |
| Iran (Islamic Republic of) | 109852 (51617-311719) | 208.29 (106.82-532.16) | 63915 (43664-90892) | 76.4 (52.24-108.34) | -2.89 (-3.24--2.54) |
| Iraq | 57470 (24093-249711) | 301.95 (144.64-1086.85) | 55472 (34223-88948) | 139.52 (87.36-218.74) | -3.33 (-3.57--3.09) |
| Ireland | 278 (172-418) | 8.27 (5.13-12.37) | 202 (134-303) | 4.53 (2.94-6.94) | -2.32 (-2.51--2.12) |
| Israel | 1258 (776-1882) | 25.88 (15.97-38.89) | 2298 (1261-3850) | 21.58 (12.08-35.58) | -0.59 (-0.69--0.5) |
| Italy | 5872 (4755-7440) | 12.26 (9.56-15.99) | 3664 (2791-4693) | 6.94 (5.06-9.22) | -2.53 (-2.81--2.25) |
| Jamaica | 2828 (2022-3875) | 125.03 (88.68-169.03) | 2875 (1942-3984) | 104.45 (71.25-146.63) | -0.34 (-0.45--0.24) |
| Japan | 8722 (5676-12775) | 7.28 (4.82-11.03) | 8809 (5569-12778) | 5.88 (3.68-8.92) | -0.4 (-0.73--0.08) |
| Jordan | 3369 (1916-9636) | 82.04 (50.06-205.48) | 4933 (3261-7403) | 40.23 (26.85-59.71) | -2.34 (-2.46--2.21) |
| Kazakhstan | 16366 (11343-23560) | 99.68 (69.48-142.8) | 11767 (7974-17094) | 60.96 (41.28-88.42) | -2.05 (-2.28--1.81) |
| Kenya | 1187875 (814506-1830364) | 3633.39 (2517.57-5591.53) | 749715 (509935-1066990) | 1457.3 (1000.83-2056.99) | -5.52 (-6.52--4.5) |
| Kiribati | 691 (488-974) | 1045.57 (744.12-1472.76) | 318 (182-615) | 274.25 (163.32-512.78) | -4.78 (-5.27--4.29) |
| Kuwait | 558 (377-808) | 31.21 (21.23-45.25) | 969 (652-1408) | 21.79 (14.52-32.48) | -1.15 (-1.27--1.03) |
| Kyrgyzstan | 5018 (3665-7079) | 102.16 (75.21-144.12) | 5116 (3475-8100) | 71.04 (48.18-110.31) | -1.34 (-1.43--1.24) |
| Lao People's Democratic Republic | 40372 (29521-52912) | 1050.81 (753.92-1390.63) | 19038 (13474-25890) | 272.21 (194.21-369.33) | -4.45 (-5.12--3.78) |
| Latvia | 1613 (1037-2317) | 57.93 (38.44-81.26) | 933 (545-1551) | 41.36 (25.85-63.79) | -1.28 (-1.42--1.14) |
| Lebanon | 2426 (1356-7404) | 77.88 (45-225.26) | 1794 (1222-2516) | 33.19 (22.83-46.9) | -2.57 (-2.77--2.38) |
| Lesotho | 6669 (4440-9919) | 417.24 (287.01-618.07) | 2906 (2159-3762) | 171.72 (126.79-226.39) | -2.92 (-3.38--2.45) |
| Liberia | 503510 (272135-835434) | 14662.43 (8774.58-23275.87) | 486230 (237064-817054) | 8957.32 (4430.33-15274.18) | -2.41 (-2.97--1.84) |
| Libya | 7509 (4372-14054) | 190.31 (115.58-325.34) | 9646 (5969-15916) | 138.8 (87.05-225.49) | -0.88 (-1.06--0.7) |
| Lithuania | 2284 (1418-3667) | 58.73 (37.23-91.19) | 1457 (838-2335) | 42.29 (26.05-64.54) | -1.1 (-1.17--1.02) |
| Luxembourg | 22 (14-34) | 7.26 (4.46-11.21) | 28 (20-40) | 4.64 (3.15-7.06) | -1.51 (-1.77--1.25) |
| Madagascar | 557003 (363783-822030) | 3612.02 (2444.44-5118.54) | 567287 (196878-1126331) | 1827.98 (672.62-3551.99) | -2.77 (-3.41--2.13) |
| Malawi | 2001610 (1328693-3114691) | 12957.32 (8640.73-19874.69) | 696673 (319487-1298749) | 3281.63 (1502.69-6296.63) | -5.53 (-5.93--5.13) |
| Malaysia | 67357 (46892-91046) | 368.04 (257.92-492.55) | 44144 (31218-61266) | 138.73 (98.72-193.5) | -3.36 (-3.8--2.91) |
| Maldives | 750 (455-1132) | 271.1 (171.27-388.15) | 724 (366-1588) | 152.29 (79.72-314.09) | -2.28 (-3.29--1.25) |
| Mali | 1763509 (1013982-3042005) | 12888.28 (7643.26-21620.9) | 2025153 (1065667-3788446) | 5873.38 (3127.07-11238.4) | -2.36 (-2.67--2.05) |
| Malta | 117 (27-704) | 34.75 (7.93-211.48) | 40 (13-194) | 12.22 (3.67-64.55) | -3.94 (-4.34--3.54) |
| Marshall Islands | 156 (86-361) | 321.11 (185.87-755.55) | 122 (59-341) | 219.51 (109.4-607.9) | -1.23 (-1.41--1.04) |
| Mauritania | 26433 (12762-58593) | 1083.07 (559.27-2351.1) | 33924 (15475-60150) | 734.28 (326.23-1344.87) | -1.4 (-3.03-0.26) |
| Mauritius | 4427 (2351-8389) | 396.95 (218.16-746.27) | 4619 (2456-8868) | 341.97 (179.17-659.45) | -0.42 (-0.46--0.37) |
| Mexico | 188181 (153474-234680) | 242.89 (193.85-305.5) | 171827 (124932-231329) | 132.04 (97.07-177.25) | -1.54 (-1.92--1.16) |
| Micronesia (Federated States of) | 506 (351-709) | 535.95 (375.28-746.09) | 139 (94-196) | 143.06 (97.25-197.98) | -3.49 (-4.18--2.79) |
| Monaco | 10 (5-19) | 22.29 (12.74-38.55) | 17 (11-29) | 45.26 (32.73-61.56) | 2.2 (1.39-3.02) |
| Mongolia | 3421 (2380-4707) | 154.35 (109.64-211.15) | 3318 (2215-4527) | 98.9 (66.48-134.89) | -1.76 (-1.88--1.65) |
| Montenegro | 294 (167-663) | 47.69 (27.32-109.78) | 233 (126-445) | 35.08 (18.73-79.31) | -1.25 (-1.52--0.98) |
| Morocco | 29581 (21456-42219) | 116.8 (87.2-160.07) | 25205 (17442-35822) | 69.28 (48.17-98.45) | -1.51 (-1.59--1.43) |
| Mozambique | 2706307 (1752374-4349794) | 14892.12 (9602.02-23807.91) | 1980707 (781406-4008748) | 6060.89 (2314.51-12674.89) | -3.69 (-4--3.37) |
| Myanmar | 656185 (458356-1062385) | 1512.28 (1063.84-2388.76) | 139937 (100258-197859) | 252.19 (180.91-354.69) | -4.7 (-6.15--3.23) |
| Namibia | 21696 (8039-58058) | 1502.97 (603.8-3936.25) | 10450 (6809-19143) | 440.14 (296.98-777.05) | -4.28 (-5.45--3.09) |
| Nauru | 14 (9-20) | 135.15 (94.16-186.41) | 10 (7-15) | 94.88 (63.67-135.8) | -1.15 (-1.34--0.95) |
| Nepal | 323296 (206235-582522) | 1585.41 (1076.74-2595.35) | 95881 (69868-128829) | 322.2 (239.17-426.94) | -5.65 (-6.01--5.3) |
| Netherlands | 982 (659-1394) | 7.96 (5.26-11.72) | 869 (627-1178) | 5.67 (3.86-7.93) | -1.18 (-1.37--1) |
| New Zealand | 254 (159-456) | 7.94 (4.88-14.66) | 242 (146-421) | 5.36 (2.99-10.29) | -1.3 (-1.37--1.22) |
| Nicaragua | 13029 (9817-16825) | 337.22 (250.27-439.06) | 17017 (11261-22779) | 263.71 (177.55-352.03) | -1.78 (-2.06--1.5) |
| Niger | 1605497 (773199-2989701) | 12324.49 (6007.91-22765.48) | 2702190 (1102813-4637622) | 7835.28 (3072.29-13893.7) | -1.29 (-1.59--0.98) |
| Nigeria | 13196484 (7819813-21448195) | 10945.98 (6693.41-17289.84) | 18220693 (7908906-34285303) | 6730.94 (2942.71-13073.12) | -1.93 (-2.23--1.62) |
| Niue | 5 (3-7) | 212.62 (149.2-299.15) | 2 (1-3) | 116.21 (79.77-161.04) | -2.23 (-2.61--1.86) |
| North Macedonia | 1336 (821-2827) | 68.29 (41.85-146.15) | 898 (534-1467) | 38.34 (23.65-66.7) | -2.14 (-2.29--2) |
| Northern Mariana Islands | 27 (18-39) | 64.94 (44.64-92.29) | 25 (16-37) | 53.84 (35.9-79.86) | -0.42 (-0.58--0.26) |
| Norway | 250 (164-364) | 6.95 (4.38-10.41) | 255 (188-345) | 4.76 (3.17-7.04) | -1.24 (-1.39--1.09) |
| Oman | 4089 (2633-7684) | 315.62 (222.47-472.13) | 2436 (1584-3840) | 70.65 (48.4-103.12) | -5.49 (-6.34--4.64) |
| Pakistan | 1417351 (538975-5013627) | 1071.36 (417.8-3979.45) | 1106176 (536039-2209296) | 429.85 (213.9-850.75) | -2.98 (-3.72--2.23) |
| Palau | 21 (12-48) | 141.6 (80.05-322.91) | 19 (10-49) | 108.99 (55.88-285.04) | -1.02 (-1.31--0.72) |
| Palestine | 1952 (965-7362) | 81.7 (43.42-266.57) | 1538 (972-2321) | 29.99 (19.3-43.96) | -2.97 (-3.22--2.73) |
| Panama | 5361 (3982-7207) | 239.37 (177.22-324.93) | 7178 (4965-9715) | 166.05 (115.38-224.92) | -1.09 (-1.17--1.01) |
| Papua New Guinea | 192265 (80630-472342) | 4649.4 (2020.49-11668.17) | 168380 (90453-286895) | 1620.85 (869.54-2814.8) | -3.15 (-3.75--2.55) |
| Paraguay | 9227 (6603-12984) | 263.74 (200.69-357.24) | 9905 (6987-13688) | 145.67 (103.08-199.69) | -1.06 (-1.41--0.7) |
| Peru | 147460 (78466-274401) | 690.66 (380.29-1231.59) | 88977 (60359-124852) | 245.63 (167.75-343.93) | -3.46 (-3.76--3.16) |
| Philippines | 442251 (328457-614214) | 685.93 (506.03-943.28) | 298661 (235793-374946) | 263.74 (208.97-330.18) | -3.01 (-3.44--2.58) |
| Poland | 26307 (18461-35840) | 67.04 (47.3-90.93) | 19861 (12767-29109) | 41.69 (27.8-59.74) | -1.56 (-1.62--1.51) |
| Portugal | 5091 (2530-8627) | 49.34 (25.31-88.29) | 2788 (1270-4716) | 22.87 (11.17-38.47) | -3.05 (-3.38--2.72) |
| Puerto Rico | 3714 (2113-6435) | 104.52 (60.26-179.6) | 2641 (1214-5128) | 65.29 (30.71-135.1) | -1.6 (-1.75--1.46) |
| Qatar | 126 (80-185) | 27.55 (17.62-40.23) | 359 (216-542) | 14.06 (8.75-21) | -2.73 (-2.92--2.55) |
| Republic of Korea | 36370 (25744-49644) | 88.82 (62.72-121.71) | 45414 (30878-63015) | 62.64 (43.33-86.5) | -1.05 (-1.22--0.87) |
| Republic of Moldova | 3337 (2242-4651) | 74.03 (50.07-103.3) | 1821 (1155-2611) | 43.05 (28.24-59.68) | -1.95 (-2.07--1.82) |
| Romania | 22571 (13175-57199) | 96.47 (54.73-263.85) | 9741 (5850-16146) | 41.83 (26.6-65.12) | -2.92 (-3.12--2.71) |
| Russian Federation | 116729 (84337-155661) | 73.99 (54.13-97.25) | 79948 (56487-108239) | 47.07 (34.55-62.63) | -1.76 (-1.92--1.6) |
| Rwanda | 798055 (432885-1488334) | 7854.5 (4357.57-14741.29) | 262002 (163349-417803) | 1789.25 (1128.88-2860.93) | -6.25 (-7.05--5.45) |
| Saint Kitts and Nevis | 60 (41-84) | 159.12 (104.68-225.39) | 50 (30-79) | 79.36 (49.78-118.65) | -2.3 (-2.49--2.12) |
| Saint Lucia | 266 (184-374) | 208.53 (144.83-297.45) | 189 (124-275) | 100.86 (69.15-140.82) | -2.74 (-3.09--2.39) |
| Saint Vincent and the Grenadines | 182 (133-243) | 180.91 (130.82-242.6) | 122 (78-183) | 102.59 (67.71-150.83) | -1.73 (-1.8--1.66) |
| Samoa | 731 (518-991) | 489.08 (345.72-658.02) | 408 (278-580) | 211.48 (145.96-296.11) | -2.09 (-2.96--1.21) |
| San Marino | 6 (3-10) | 19.9 (11.25-33.58) | 7 (4-13) | 14.58 (7.85-25.54) | -1.06 (-1.13--1) |
| Sao Tome and Principe | 3868 (1766-7627) | 2889.38 (1217.42-6053.17) | 1453 (1011-1967) | 656.18 (468.75-865.98) | -6.34 (-8.07--4.57) |
| Saudi Arabia | 25092 (15831-45638) | 166.97 (109.81-276.89) | 23600 (15148-34570) | 64.32 (42.5-93.88) | -3.29 (-3.55--3.03) |
| Senegal | 509570 (224375-1110202) | 5274.11 (2403.15-11296.21) | 374662 (207628-508444) | 2328.24 (1292.67-3237.09) | -4.15 (-5.08--3.22) |
| Serbia | 8254 (4458-19291) | 83.77 (43.94-218.9) | 4733 (2684-7973) | 42.01 (25.24-67.64) | -2.4 (-2.55--2.26) |
| Seychelles | 169 (75-439) | 220.25 (96.12-578.81) | 156 (50-515) | 153.19 (50.27-501.35) | -1.22 (-1.78--0.66) |
| Sierra Leone | 822204 (393099-1385593) | 14319.52 (7091.73-24166.99) | 969790 (390593-1786967) | 9853.4 (3935.94-18228.18) | -1.68 (-2.13--1.23) |
| Singapore | 2541 (595-6222) | 83.72 (19.44-204.73) | 5002 (1037-12527) | 91.02 (18.46-227.25) | 0.98 (-0.09-2.06) |
| Slovakia | 3429 (2155-5118) | 62.99 (39.85-93.25) | 2818 (1510-4902) | 41.26 (23.33-66.88) | -1.38 (-1.42--1.33) |
| Slovenia | 481 (275-1216) | 27.43 (15.37-73.43) | 220 (143-331) | 12.52 (7.98-18.78) | -2.82 (-2.98--2.65) |
| Solomon Islands | 13436 (2977-38394) | 5545.42 (1029.41-16881.94) | 7799 (5121-12448) | 1572.01 (1022.2-2610.93) | -4.82 (-5.85--3.77) |
| Somalia | 834148 (537324-1349366) | 7651.96 (5089.15-11804.23) | 331532 (205641-533899) | 1519.41 (1021.73-2382.18) | -5.99 (-6.57--5.41) |
| South Africa | 175270 (121689-248204) | 465.77 (328.29-651.33) | 121270 (87437-172639) | 216 (157.19-302.45) | -2.91 (-3.18--2.63) |
| South Sudan | 821041 (627289-1124455) | 10753.64 (8324.73-14338.47) | 803208 (471547-1340643) | 6675.62 (4141.56-10734.33) | -1.55 (-1.92--1.17) |
| Spain | 14021 (7591-26938) | 34.65 (18.97-78.55) | 13527 (6178-25133) | 20.46 (10.36-35.23) | -1.22 (-1.47--0.96) |
| Sri Lanka | 84112 (58645-133953) | 496.44 (345.06-795.13) | 25600 (18927-38452) | 116.71 (85.82-174.36) | -5.35 (-6.24--4.46) |
| Sudan | 613358 (358500-1153443) | 2589.26 (1586.07-4498.13) | 396388 (207978-739668) | 847.69 (453.89-1585.42) | -4.6 (-5.05--4.14) |
| Suriname | 4119 (1344-16576) | 1047.36 (362.88-4131.17) | 1752 (1266-2570) | 295.79 (213.81-430.63) | -4.27 (-5.43--3.1) |
| Sweden | 516 (333-772) | 7.12 (4.54-10.89) | 493 (344-691) | 5.23 (3.44-7.8) | -1.12 (-1.23--1.02) |
| Switzerland | 339 (208-522) | 6.03 (3.65-9.27) | 352 (229-569) | 4.39 (2.68-7.44) | -0.79 (-0.95--0.63) |
| Syrian Arab Republic | 26893 (15332-45057) | 245.24 (133.67-420.86) | 49645 (33099-69793) | 353.82 (236.53-498.38) | 1.21 (0.77-1.66) |
| Taiwan (Province of China) | 17018 (9967-26828) | 84.63 (49.95-130.67) | 6135 (3906-9480) | 21.19 (13.94-31.18) | -4.6 (-5.33--3.87) |
| Tajikistan | 35509 (10008-88899) | 430.28 (150.7-1028.11) | 8940 (5942-12757) | 83.77 (56.16-118.7) | -5.29 (-5.85--4.73) |
| Thailand | 402460 (276097-555871) | 752.7 (517.95-1041.47) | 83027 (52766-116693) | 107.11 (72.76-144.6) | -6.22 (-6.36--6.08) |
| Timor-Leste | 14434 (8430-28338) | 1636.68 (1024.68-2842.61) | 4014 (2876-5434) | 316.15 (226.53-428.73) | -6.89 (-9.44--4.28) |
| Togo | 388237 (222207-627450) | 8040.28 (4694.84-12851.33) | 413008 (182370-710906) | 4823.32 (2120.13-8542.53) | -1.27 (-1.73--0.8) |
| Tokelau | 2 (2-4) | 144.62 (99.46-213.78) | 1 (1-1) | 74.53 (52.03-99.13) | -2.46 (-2.66--2.26) |
| Tonga | 194 (125-287) | 203.11 (130.42-288.1) | 272 (86-701) | 257.17 (83.04-653.11) | 1.89 (1.27-2.52) |
| Trinidad and Tobago | 1539 (1067-2120) | 136.38 (93.94-187.48) | 1565 (1039-2271) | 104.33 (70.75-148.02) | -0.83 (-1.03--0.63) |
| Tunisia | 9758 (5686-21789) | 117.41 (69.6-235.82) | 11638 (7770-16641) | 96.82 (64.33-139) | -0.41 (-0.6--0.22) |
| Turkey | 72885 (41181-243099) | 123.65 (73.19-377.45) | 28010 (19607-38657) | 34.49 (23.8-47.26) | -4.19 (-4.46--3.91) |
| Turkmenistan | 7246 (4737-10099) | 201.97 (130.48-286) | 5435 (3550-7645) | 108.9 (70.81-153.18) | -2.33 (-2.45--2.22) |
| Tuvalu | 18 (12-25) | 183.53 (126.82-261.52) | 10 (7-14) | 84.01 (57.71-116.25) | -2.29 (-2.5--2.09) |
| Uganda | 4097127 (2802745-6057498) | 15277.52 (10739.44-22028.1) | 3815880 (1417126-6508239) | 6436.08 (2389.33-11495.89) | -4.92 (-5.62--4.23) |
| Ukraine | 42675 (28015-60205) | 75.04 (50.34-102.87) | 40495 (25158-58464) | 75.58 (49.76-105.11) | 0.18 (0.03-0.34) |
| United Arab Emirates | 565 (370-860) | 28.4 (18.86-41.89) | 1415 (851-2135) | 20.28 (12.4-30.69) | -1.05 (-1.15--0.95) |
| United Kingdom | 5000 (3536-7100) | 10.73 (7.43-15.39) | 3833 (2521-5882) | 7.58 (4.92-11.75) | -1.12 (-1.26--0.99) |
| United Republic of Tanzania | 3271032 (1893143-5124043) | 8225.21 (5018.64-12570.69) | 1490871 (659463-2758034) | 2018.65 (951.94-3756.89) | -5.69 (-6.3--5.08) |
| United States of America | 67724 (43305-101748) | 23.76 (15.32-35.61) | 118831 (78069-178762) | 26.66 (18.07-38.41) | 0.5 (0.05-0.95) |
| United States Virgin Islands | 83 (53-119) | 80.97 (51.3-117.29) | 63 (36-102) | 57.36 (36.79-87.83) | -1.17 (-1.21--1.13) |
| Uruguay | 2017 (1376-2899) | 59.78 (40.85-85.97) | 1883 (1146-3005) | 43.86 (26.85-68.62) | -0.83 (-0.9--0.76) |
| Uzbekistan | 23992 (15900-34246) | 102.1 (68.31-144.4) | 27600 (18936-39020) | 78.29 (53.64-110.9) | -1 (-1.09--0.91) |
| Vanuatu | 3880 (2161-6839) | 3311.22 (1758.63-6094.35) | 591 (407-862) | 190.75 (134.29-275.84) | -7.81 (-9.7--5.88) |
| Venezuela (Bolivarian Republic of) | 93787 (76996-115513) | 628.86 (517.37-759.16) | 95061 (71375-127675) | 351.33 (262.09-481.42) | -1.87 (-2.29--1.45) |
| Viet Nam | 513676 (351278-737637) | 763.47 (525.02-1084.04) | 149185 (105378-201875) | 147.89 (104.02-200.64) | -6.12 (-6.46--5.78) |
| Yemen | 479403 (195093-1012446) | 3563.94 (1521.16-7467.58) | 577084 (184479-1179171) | 1908.24 (570.59-4143.23) | -2.21 (-3.13--1.29) |
| Zambia | 843636 (592238-1280216) | 7748.45 (5469.85-11563.39) | 565811 (225729-1095292) | 2632.7 (1038.62-5236.65) | -4.59 (-5.18--4.01) |
| Zimbabwe | 285758 (152605-498025) | 2226.23 (1267.44-3832.32) | 194697 (117895-332723) | 1097.09 (713.1-1800.88) | 0.12 (-0.93-1.17) |

**Table S5.** The predicted results in the neglected tropical diseases and malaria-related numbers and age-standardized rates of incidence, prevalence, deaths, DALYs by sex globally from 2022 to 2046 of the APC model. Abbreviations: DALYs, disability-adjusted-life-years; APC, age-period-cohort.

| year | sex | Age-standardized incidence rate | Numer of incidence cases | Age-standardized prevalence rate | Numer of prevalence cases | Age-standardized deaths rate | Numer of deaths cases | Age-standardized DALYs rate | Numer of DALYs cases |
| --- | --- | --- | --- | --- | --- | --- | --- | --- | --- |
| 2022 | Female | 4309.82 | 156633332.9 | 15155.89 | 576392874.6 | 10.8 | 401726.4 | 977.72 | 35312832 |
| 2023 | Female | 4293.3 | 156554693 | 15092.43 | 576246967.2 | 10.85 | 406154.4 | 983.19 | 35627755 |
| 2024 | Female | 4276.79 | 156395467.5 | 15028.96 | 575765542.5 | 10.91 | 410317.2 | 988.66 | 35918648 |
| 2025 | Female | 4267.99 | 156457987.1 | 14912.68 | 573635172.6 | 10.89 | 411794.3 | 986.81 | 35945946 |
| 2026 | Female | 4259.2 | 156444355.3 | 14796.4 | 571236135.2 | 10.87 | 413026.6 | 984.97 | 35952629 |
| 2027 | Female | 4250.41 | 156366012.3 | 14680.11 | 568596382.2 | 10.85 | 414031.5 | 983.12 | 35941991 |
| 2028 | Female | 4241.62 | 156218194.9 | 14563.83 | 565691000.8 | 10.82 | 414777.9 | 981.27 | 35911296 |
| 2029 | Female | 4232.83 | 155992520.8 | 14447.55 | 562484778.4 | 10.8 | 415254.6 | 979.43 | 35858343 |
| 2030 | Female | 4227.14 | 155856224.8 | 14375.1 | 561039298.1 | 10.79 | 415851.5 | 978.18 | 35818907 |
| 2031 | Female | 4221.45 | 155670112.5 | 14302.65 | 559380340.4 | 10.77 | 416334.1 | 976.94 | 35769079 |
| 2032 | Female | 4215.76 | 155432184.4 | 14230.2 | 557533576.7 | 10.76 | 416687.1 | 975.7 | 35706700 |
| 2033 | Female | 4210.07 | 155197259.9 | 14157.76 | 555483817.3 | 10.74 | 417218 | 974.46 | 35657056 |
| 2034 | Female | 4204.38 | 154910892.4 | 14085.31 | 553197835.5 | 10.72 | 417671.3 | 973.22 | 35596992 |
| 2035 | Female | 4201.41 | 154723289.5 | 14050.78 | 552547441.9 | 10.71 | 418290.5 | 972.51 | 35555584 |
| 2036 | Female | 4198.44 | 154501927.1 | 14016.24 | 551754449 | 10.7 | 418800.1 | 971.8 | 35506978 |
| 2037 | Female | 4195.47 | 154249748.8 | 13981.71 | 550819054.8 | 10.69 | 419174.5 | 971.09 | 35451517 |
| 2038 | Female | 4192.5 | 153978939.4 | 13947.18 | 549822307.8 | 10.68 | 419417.8 | 970.38 | 35389812 |
| 2039 | Female | 4189.53 | 153676450.2 | 13912.65 | 548658755.2 | 10.67 | 419561.7 | 969.67 | 35322242 |
| 2040 | Female | 4186.75 | 153371519.6 | 13879.03 | 547419699 | 10.66 | 419784.9 | 969.03 | 35256331 |
| 2041 | Female | 4183.97 | 153045015.9 | 13845.41 | 546053634.5 | 10.65 | 419942 | 968.38 | 35187503 |
| 2042 | Female | 4181.2 | 152697012.1 | 13811.79 | 544571705.6 | 10.64 | 420030.8 | 967.74 | 35115554 |
| 2043 | Female | 4178.42 | 152330327.3 | 13778.17 | 542970211.5 | 10.63 | 420046.7 | 967.1 | 35039552 |
| 2044 | Female | 4175.64 | 151934273.1 | 13744.55 | 541221540 | 10.62 | 419975.4 | 966.46 | 34957791 |
| 2045 | Female | 4172.86 | 151509352 | 13710.93 | 539345487.1 | 10.61 | 419816.3 | 965.82 | 34870366 |
| 2046 | Female | 4170.08 | 151056273.2 | 13677.31 | 537359672.5 | 10.6 | 419569.9 | 965.18 | 34777191 |
| 2022 | Male | 4008.77 | 152334106.3 | 14769.48 | 579833701.1 | 11.75 | 452649.5 | 994.72 | 37749265 |
| 2023 | Male | 3991.85 | 152233202.6 | 14670.34 | 578726459.9 | 11.78 | 456836.1 | 997.16 | 37973724 |
| 2024 | Male | 3974.93 | 152043348.9 | 14571.2 | 577218579.1 | 11.81 | 460714.2 | 999.61 | 38168963 |
| 2025 | Male | 3965.87 | 152091057.3 | 14431.52 | 574285846.9 | 11.79 | 462365.5 | 996.04 | 38130965 |
| 2026 | Male | 3956.8 | 152057279.2 | 14291.83 | 571030545.4 | 11.76 | 463740.5 | 992.47 | 38068804 |
| 2027 | Male | 3947.73 | 151955074.4 | 14152.15 | 567481737.7 | 11.74 | 464859.7 | 988.9 | 37986184 |
| 2028 | Male | 3938.67 | 151781231.3 | 14012.47 | 563618933.6 | 11.71 | 465711.9 | 985.34 | 37881217 |
| 2029 | Male | 3929.6 | 151526667.9 | 13872.78 | 559409542 | 11.69 | 466287.7 | 981.77 | 37751427 |
| 2030 | Male | 3923.99 | 151371378.6 | 13789.5 | 557497941 | 11.67 | 467363.2 | 979.62 | 37670024 |
| 2031 | Male | 3918.37 | 151160567.9 | 13706.22 | 555343980 | 11.66 | 468289.2 | 977.47 | 37574511 |
| 2032 | Male | 3912.76 | 150889677.4 | 13622.94 | 552973178.7 | 11.65 | 469031.5 | 975.32 | 37461530 |
| 2033 | Male | 3907.14 | 150631574.2 | 13539.66 | 550387921.6 | 11.64 | 469963.2 | 973.17 | 37362708 |
| 2034 | Male | 3901.53 | 150324104.5 | 13456.37 | 547551154.4 | 11.63 | 470810 | 971.02 | 37252370 |
| 2035 | Male | 3899.08 | 150136730.1 | 13420.84 | 546822221.3 | 11.63 | 472238.8 | 970.1 | 37194942 |
| 2036 | Male | 3896.64 | 149917570 | 13385.31 | 545946308.3 | 11.63 | 473572.1 | 969.18 | 37130644 |
| 2037 | Male | 3894.19 | 149668565.4 | 13349.78 | 544913000.4 | 11.62 | 474790.3 | 968.26 | 37059952 |
| 2038 | Male | 3891.74 | 149408251.2 | 13314.25 | 543866006 | 11.62 | 475907 | 967.34 | 36984617 |
| 2039 | Male | 3889.3 | 149120618.2 | 13278.72 | 542667021 | 11.62 | 476951 | 966.41 | 36904103 |
| 2040 | Male | 3886.7 | 148815252.6 | 13242.33 | 541311154.6 | 11.63 | 478114.8 | 965.51 | 36823712 |
| 2041 | Male | 3884.1 | 148492112.6 | 13205.94 | 539839366.7 | 11.63 | 479225.5 | 964.6 | 36741030 |
| 2042 | Male | 3881.5 | 148151252.5 | 13169.55 | 538261949.3 | 11.63 | 480275.1 | 963.69 | 36655826 |
| 2043 | Male | 3878.9 | 147795637.2 | 13133.16 | 536578142.2 | 11.63 | 481256.8 | 962.78 | 36566993 |
| 2044 | Male | 3876.3 | 147415181.9 | 13096.77 | 534767651.9 | 11.63 | 482161.1 | 961.88 | 36472881 |
| 2045 | Male | 3873.71 | 147010489.8 | 13060.39 | 532847546 | 11.63 | 482989.9 | 960.97 | 36373649 |
| 2046 | Male | 3871.11 | 146581744.6 | 13024 | 530830107.5 | 11.63 | 483747.4 | 960.06 | 36269236 |

**Table S6.** The predicted results in the neglected tropical diseases and malaria-related numbers and age-standardized rates of incidence, prevalence, deaths, DALYs by sex globally from 2022 to 2046 of the ARIMA model. Abbreviations: DALYs, disability-adjusted-life-years; ARIMA, Autoregressive Integrated Moving Average.

| year | sex | Age-standardized incidence rate | Numer of incidence cases | Age-standardized prevalence rate | Numer of prevalence cases | Age-standardized deaths rate | Numer of deaths cases | Age-standardized DALYs rate | Numer of DALYs cases |
| --- | --- | --- | --- | --- | --- | --- | --- | --- | --- |
| 2022 | Male | 4109.732 | 153516021.3 | 13895.47 | 545816464.9 | 12.33837 | 450532.9 | 1033.181 | 37053835 |
| 2023 | Male | 4109.732 | 154449373 | 13438.93 | 533553320.6 | 12.34463 | 450441.1 | 1033.863 | 37006717 |
| 2024 | Male | 4109.732 | 155382724.8 | 13046.72 | 524526678.5 | 12.34807 | 450403 | 1034.319 | 36982495 |
| 2025 | Male | 4109.732 | 156316076.5 | 12752.25 | 519090247 | 12.34996 | 450387.1 | 1034.624 | 36970043 |
| 2026 | Male | 4109.732 | 157249428.3 | 12470.21 | 513720176.8 | 12.351 | 450380.5 | 1034.827 | 36963642 |
| 2027 | Male | 4109.732 | 158182780 | 12121.84 | 505970770.3 | 12.35157 | 450377.8 | 1034.963 | 36960351 |
| 2028 | Male | 4109.732 | 159116131.8 | 11722.48 | 496982370.7 | 12.35188 | 450376.6 | 1035.053 | 36958659 |
| 2029 | Male | 4109.732 | 160049483.6 | 11341.5 | 489291113.3 | 12.35205 | 450376.1 | 1035.114 | 36957790 |
| 2030 | Male | 4109.732 | 160982835.3 | 11010.69 | 483590569.2 | 12.35215 | 450375.9 | 1035.154 | 36957343 |
| 2031 | Male | 4109.732 | 161916187.1 | 10697.38 | 478388328.1 | 12.3522 | 450375.9 | 1035.181 | 36957113 |
| 2032 | Male | 4109.732 | 162849538.8 | 10356.97 | 472234792.3 | 12.35223 | 450375.8 | 1035.199 | 36956995 |
| 2033 | Male | 4109.732 | 163782890.6 | 9986.327 | 465354220.7 | 12.35224 | 450375.8 | 1035.211 | 36956934 |
| 2034 | Male | 4109.732 | 164716242.4 | 9617.288 | 458969459.1 | 12.35225 | 450375.8 | 1035.219 | 36956903 |
| 2035 | Male | 4109.732 | 165649594.1 | 9272.189 | 453657102.8 | 12.35226 | 450375.8 | 1035.225 | 36956887 |
| 2036 | Male | 4109.732 | 166582945.9 | 8940.866 | 448843606.7 | 12.35226 | 450375.8 | 1035.228 | 36956878 |
| 2037 | Male | 4109.732 | 167516297.6 | 8600.017 | 443734490.4 | 12.35226 | 450375.8 | 1035.231 | 36956874 |
| 2038 | Male | 4109.732 | 168449649.4 | 8242.752 | 438266147.7 | 12.35226 | 450375.8 | 1035.232 | 36956872 |
| 2039 | Male | 4109.732 | 169383001.1 | 7882.431 | 432991671.3 | 12.35226 | 450375.8 | 1035.233 | 36956871 |
| 2040 | Male | 4109.732 | 170316352.9 | 7532.643 | 428293317.4 | 12.35226 | 450375.8 | 1035.234 | 36956870 |
| 2041 | Male | 4109.732 | 171249704.7 | 7191.772 | 423983648.5 | 12.35226 | 450375.8 | 1035.235 | 36956870 |
| 2042 | Male | 4109.732 | 172183056.4 | 6848.498 | 419653392.9 | 12.35226 | 450375.8 | 1035.235 | 36956870 |
| 2043 | Male | 4109.732 | 173116408.2 | 6496.956 | 415186173.7 | 12.35226 | 450375.8 | 1035.235 | 36956870 |
| 2044 | Male | 4109.732 | 174049759.9 | 6142.057 | 410813293.3 | 12.35226 | 450375.8 | 1035.235 | 36956870 |
| 2045 | Male | 4109.732 | 174983111.7 | 5791.327 | 406759587.4 | 12.35226 | 450375.8 | 1035.235 | 36956870 |
| 2046 | Male | 4109.732 | 175916463.5 | 5445.753 | 402984088.8 | 12.35226 | 450375.8 | 1035.235 | 36956870 |
| 2047 | Male | 4109.732 | 176849815.2 | 5100.248 | 399287212.7 | 12.35226 | 450375.8 | 1035.235 | 36956870 |
| 2048 | Male | 4109.732 | 177783167 | 4750.875 | 395572167.3 | 12.35226 | 450375.8 | 1035.235 | 36956870 |
| 2049 | Male | 4109.732 | 178716518.7 | 4399.028 | 391925130.6 | 12.35226 | 450375.8 | 1035.235 | 36956870 |
| 2050 | Male | 4109.732 | 179649870.5 | 4048.563 | 388467625.5 | 12.35226 | 450375.8 | 1035.235 | 36956870 |
| 2022 | Female | 4418.831 | 158305035.5 | 14334.79 | 547475939.2 | 11.29919 | 396538.8 | 1010.383 | 34414747 |
| 2023 | Female | 4421.557 | 159389978.6 | 13955.78 | 538372989.8 | 11.30651 | 396471.4 | 1011.146 | 34381852 |
| 2024 | Female | 4423.996 | 160474921.7 | 13624.06 | 531645679.1 | 11.31047 | 396446.1 | 1011.624 | 34366321 |
| 2025 | Female | 4426.178 | 161559864.8 | 13370.04 | 527731062.2 | 11.31262 | 396436.6 | 1011.922 | 34358988 |
| 2026 | Female | 4428.13 | 162644807.9 | 13130.44 | 524064662.4 | 11.31379 | 396433.1 | 1012.109 | 34355527 |
| 2027 | Female | 4429.876 | 163729751.1 | 12842.18 | 518701696.4 | 11.31442 | 396431.7 | 1012.226 | 34353892 |
| 2028 | Female | 4431.438 | 164814694.2 | 12512.11 | 512310297.8 | 11.31476 | 396431.2 | 1012.299 | 34353121 |
| 2029 | Female | 4432.835 | 165899637.3 | 12191.55 | 506742579.3 | 11.31495 | 396431 | 1012.345 | 34352756 |
| 2030 | Female | 4434.085 | 166984580.4 | 11908.21 | 502660079.1 | 11.31505 | 396431 | 1012.373 | 34352584 |
| 2031 | Female | 4435.203 | 168069523.5 | 11641.57 | 499099287.8 | 11.3151 | 396430.9 | 1012.391 | 34352503 |
| 2032 | Female | 4436.203 | 169154466.6 | 11357.87 | 494957160.1 | 11.31513 | 396430.9 | 1012.402 | 34352465 |
| 2033 | Female | 4437.098 | 170239409.7 | 11050.85 | 490246758.7 | 11.31515 | 396430.9 | 1012.409 | 34352447 |
| 2034 | Female | 4437.899 | 171324352.8 | 10741.62 | 485786399.9 | 11.31516 | 396430.9 | 1012.414 | 34352438 |
| 2035 | Female | 4438.615 | 172409295.9 | 10448.35 | 482074261.8 | 11.31516 | 396430.9 | 1012.416 | 34352434 |
| 2036 | Female | 4439.256 | 173494239 | 10166.93 | 478811120.1 | 11.31516 | 396430.9 | 1012.418 | 34352432 |
| 2037 | Female | 4439.829 | 174579182.2 | 9881.187 | 475433799.1 | 11.31517 | 396430.9 | 1012.419 | 34352431 |
| 2038 | Female | 4440.341 | 175664125.3 | 9583.857 | 471803994.1 | 11.31517 | 396430.9 | 1012.42 | 34352431 |
| 2039 | Female | 4440.8 | 176749068.4 | 9282.349 | 468246205 | 11.31517 | 396430.9 | 1012.42 | 34352431 |
| 2040 | Female | 4441.21 | 177834011.5 | 8986.798 | 465061415.7 | 11.31517 | 396430.9 | 1012.42 | 34352431 |
| 2041 | Female | 4441.577 | 178918954.6 | 8698.165 | 462193264.3 | 11.31517 | 396430.9 | 1012.421 | 34352431 |
| 2042 | Female | 4441.906 | 180003897.7 | 8409.515 | 459378354.4 | 11.31517 | 396430.9 | 1012.421 | 34352431 |
| 2043 | Female | 4442.199 | 181088840.8 | 8115.701 | 456487984 | 11.31517 | 396430.9 | 1012.421 | 34352431 |
| 2044 | Female | 4442.462 | 182173783.9 | 7818.602 | 453631643.8 | 11.31517 | 396430.9 | 1012.421 | 34352431 |
| 2045 | Female | 4442.697 | 183258727 | 7523.25 | 450968980.9 | 11.31517 | 396430.9 | 1012.421 | 34352431 |
| 2046 | Female | 4442.907 | 184343670.1 | 7231.459 | 448514929.8 | 11.31517 | 396430.9 | 1012.421 | 34352431 |
| 2047 | Female | 4443.095 | 185428613.3 | 6940.644 | 446156465.4 | 11.31517 | 396430.9 | 1012.421 | 34352431 |
| 2048 | Female | 4443.264 | 186513556.4 | 6647.799 | 443807453.3 | 11.31517 | 396430.9 | 1012.421 | 34352431 |
| 2049 | Female | 4443.414 | 187598499.5 | 6352.931 | 441493584.8 | 11.31517 | 396430.9 | 1012.421 | 34352431 |
| 2050 | Female | 4443.549 | 188683442.6 | 6058.283 | 439291078.4 | 11.31517 | 396430.9 | 1012.421 | 34352431 |

**Table S7.** The predicted results in the neglected tropical diseases and malaria-related numbers and age-standardized rates of incidence, prevalence, deaths, DALYs by sex globally from 2022 to 2046 of the ES model. Abbreviations: DALYs, disability-adjusted-life-years; ES, exponential smoothing.

| year | sex | Age-standardized incidence rate | Numer of incidence cases | Age-standardized prevalence rate | Numer of prevalence cases | Age-standardized deaths rate | Numer of deaths cases | Age-standardized DALYs rate | Numer of DALYs cases |
| --- | --- | --- | --- | --- | --- | --- | --- | --- | --- |
| 2022 | Male | 4111.961 | 152686617.6 | 14066.21 | 551426055 | 12.5282 | 457222.4 | 1046.608 | 37457855 |
| 2023 | Male | 4113.972 | 152780353.4 | 13877.15 | 546982127.7 | 12.70926 | 463043.1 | 1059.608 | 37738903 |
| 2024 | Male | 4115.782 | 152864715.5 | 13707.01 | 542982593.1 | 12.87221 | 468281.7 | 1071.309 | 37991846 |
| 2025 | Male | 4117.411 | 152940641.5 | 13553.87 | 539383012 | 13.01887 | 472996.5 | 1081.84 | 38219495 |
| 2026 | Male | 4118.878 | 153008974.9 | 13416.05 | 536143389 | 13.15087 | 477239.8 | 1091.317 | 38424379 |
| 2027 | Male | 4120.197 | 153070474.9 | 13292.01 | 533227728.3 | 13.26966 | 481058.8 | 1099.847 | 38608775 |
| 2028 | Male | 4121.385 | 153125824.9 | 13180.38 | 530603633.7 | 13.37658 | 484495.9 | 1107.524 | 38774731 |
| 2029 | Male | 4122.454 | 153175639.9 | 13079.91 | 528241948.5 | 13.4728 | 487589.3 | 1114.433 | 38924091 |
| 2030 | Male | 4123.416 | 153220473.5 | 12989.48 | 526116431.9 | 13.5594 | 490373.3 | 1120.651 | 39058515 |
| 2031 | Male | 4124.282 | 153260823.6 | 12908.1 | 524203466.9 | 13.63734 | 492878.9 | 1126.248 | 39179497 |
| 2032 | Male | 4125.061 | 153297138.8 | 12834.86 | 522481798.4 | 13.70749 | 495134 | 1131.284 | 39288381 |
| 2033 | Male | 4125.762 | 153329822.4 | 12768.94 | 520932296.7 | 13.77062 | 497163.5 | 1135.817 | 39386377 |
| 2034 | Male | 4126.393 | 153359237.7 | 12709.61 | 519537745.3 | 13.82744 | 498990.1 | 1139.897 | 39474572 |
| 2035 | Male | 4126.962 | 153385711.4 | 12656.22 | 518282648.9 | 13.87858 | 500634.1 | 1143.569 | 39553949 |
| 2036 | Male | 4127.473 | 153409537.8 | 12608.16 | 517153062.3 | 13.9246 | 502113.6 | 1146.874 | 39625387 |
| 2037 | Male | 4127.933 | 153430981.5 | 12564.91 | 516136434.2 | 13.96602 | 503445.2 | 1149.848 | 39689682 |
| 2038 | Male | 4128.347 | 153450280.9 | 12525.99 | 515221469 | 14.0033 | 504643.7 | 1152.524 | 39747547 |
| 2039 | Male | 4128.72 | 153467650.3 | 12490.96 | 514398000.3 | 14.03685 | 505722.3 | 1154.934 | 39799626 |
| 2040 | Male | 4129.055 | 153483282.8 | 12459.43 | 513656878.5 | 14.06705 | 506693 | 1157.102 | 39846497 |
| 2041 | Male | 4129.357 | 153497352.1 | 12431.05 | 512989868.8 | 14.09422 | 507566.7 | 1159.053 | 39888681 |
| 2042 | Male | 4129.629 | 153510014.4 | 12405.51 | 512389560.2 | 14.11868 | 508352.9 | 1160.809 | 39926646 |
| 2043 | Male | 4129.873 | 153521410.4 | 12382.53 | 511849282.3 | 14.14069 | 509060.6 | 1162.39 | 39960815 |
| 2044 | Male | 4130.093 | 153531666.9 | 12361.84 | 511363032.3 | 14.16051 | 509697.5 | 1163.812 | 39991567 |
| 2045 | Male | 4130.291 | 153540897.7 | 12343.23 | 510925407.3 | 14.17834 | 510270.7 | 1165.093 | 40019244 |
| 2046 | Male | 4130.47 | 153549205.5 | 12326.47 | 510531544.7 | 14.19438 | 510786.6 | 1166.245 | 40044153 |
| 2047 | Male | 4130.63 | 153556682.4 | 12311.39 | 510177068.5 | 14.20883 | 511250.9 | 1167.282 | 40066571 |
| 2048 | Male | 4130.775 | 153563411.7 | 12297.82 | 509858039.8 | 14.22183 | 511668.8 | 1168.215 | 40086748 |
| 2049 | Male | 4130.904 | 153569468.1 | 12285.6 | 509570914 | 14.23352 | 512044.9 | 1169.055 | 40104906 |
| 2050 | Male | 4131.021 | 153574918.8 | 12274.61 | 509312500.8 | 14.24405 | 512383.3 | 1169.811 | 40121249 |
| 2022 | Female | 4416.441 | 157325327.5 | 14472.71 | 551767306.3 | 11.46327 | 401043.4 | 1026.327 | 34761323 |
| 2023 | Female | 4417.535 | 157420224.3 | 14313.1 | 548737787.3 | 11.62307 | 404934.9 | 1040.854 | 35010464 |
| 2024 | Female | 4418.519 | 157505631.4 | 14169.46 | 546011220.1 | 11.76688 | 408437.3 | 1053.93 | 35234691 |
| 2025 | Female | 4419.405 | 157582497.9 | 14040.18 | 543557309.7 | 11.89632 | 411589.4 | 1065.697 | 35436496 |
| 2026 | Female | 4420.203 | 157651677.6 | 13923.83 | 541348790.3 | 12.01281 | 414426.4 | 1076.288 | 35618120 |
| 2027 | Female | 4420.921 | 157713939.4 | 13819.11 | 539361122.9 | 12.11765 | 416979.6 | 1085.82 | 35781582 |
| 2028 | Female | 4421.566 | 157769975 | 13724.87 | 537572222.1 | 12.21201 | 419277.5 | 1094.398 | 35928697 |
| 2029 | Female | 4422.148 | 157820407.1 | 13640.05 | 535962211.5 | 12.29693 | 421345.6 | 1102.119 | 36061101 |
| 2030 | Female | 4422.671 | 157865795.9 | 13563.71 | 534513201.9 | 12.37336 | 423206.9 | 1109.068 | 36180265 |
| 2031 | Female | 4423.142 | 157906645.9 | 13495.01 | 533209093.3 | 12.44214 | 424882.1 | 1115.321 | 36287512 |
| 2032 | Female | 4423.566 | 157943410.8 | 13433.17 | 532035395.6 | 12.50405 | 426389.8 | 1120.95 | 36384035 |
| 2033 | Female | 4423.947 | 157976499.3 | 13377.52 | 530979067.6 | 12.55977 | 427746.7 | 1126.015 | 36470905 |
| 2034 | Female | 4424.29 | 158006278.9 | 13327.44 | 530028372.4 | 12.60991 | 428967.9 | 1130.574 | 36549088 |
| 2035 | Female | 4424.599 | 158033080.6 | 13282.36 | 529172746.7 | 12.65504 | 430067 | 1134.677 | 36619453 |
| 2036 | Female | 4424.877 | 158057202.1 | 13241.79 | 528402683.7 | 12.69566 | 431056.1 | 1138.37 | 36682781 |
| 2037 | Female | 4425.128 | 158078911.4 | 13205.28 | 527709626.9 | 12.73222 | 431946.4 | 1141.694 | 36739777 |
| 2038 | Female | 4425.353 | 158098449.8 | 13172.42 | 527085875.8 | 12.76512 | 432747.6 | 1144.685 | 36791073 |
| 2039 | Female | 4425.556 | 158116034.4 | 13142.84 | 526524499.8 | 12.79473 | 433468.7 | 1147.377 | 36837239 |
| 2040 | Female | 4425.738 | 158131860.5 | 13116.23 | 526019261.4 | 12.82138 | 434117.7 | 1149.8 | 36878789 |
| 2041 | Female | 4425.902 | 158146104 | 13092.27 | 525564546.8 | 12.84536 | 434701.8 | 1151.98 | 36916184 |
| 2042 | Female | 4426.05 | 158158923.2 | 13070.71 | 525155303.7 | 12.86695 | 435227.5 | 1153.943 | 36949839 |
| 2043 | Female | 4426.183 | 158170460.4 | 13051.31 | 524786984.9 | 12.88637 | 435700.6 | 1155.709 | 36980129 |
| 2044 | Female | 4426.303 | 158180843.9 | 13033.84 | 524455498 | 12.90386 | 436126.4 | 1157.299 | 37007390 |
| 2045 | Female | 4426.41 | 158190189.1 | 13018.13 | 524157159.8 | 12.91959 | 436509.7 | 1158.729 | 37031925 |
| 2046 | Female | 4426.507 | 158198599.7 | 13003.98 | 523888655.4 | 12.93376 | 436854.6 | 1160.017 | 37054006 |
| 2047 | Female | 4426.595 | 158206169.3 | 12991.25 | 523647001.4 | 12.9465 | 437165 | 1161.176 | 37073879 |
| 2048 | Female | 4426.673 | 158212981.9 | 12979.79 | 523429512.9 | 12.95797 | 437444.4 | 1162.219 | 37091765 |
| 2049 | Female | 4426.744 | 158219113.3 | 12969.48 | 523233773.1 | 12.9683 | 437695.8 | 1163.157 | 37107862 |
| 2050 | Female | 4426.807 | 158224631.5 | 12960.2 | 523057607.4 | 12.97759 | 437922.1 | 1164.002 | 37122350 |

**Table S8.** Changes in neglected tropical diseases and malaria-related incidence number according to population-level determinants and causes from 1990 to 2021.

| location | Overll difference | varname | value |
| --- | --- | --- | --- |
| Low SDI | -71482943.82 | Aging | -31859269.06 |
| Low SDI | -71482943.82 | Population | -31859269.06 |
| Low SDI | -71482943.82 | Epidemiological change | -7764405.7 |
| Low-middle SDI | -55285646.13 | Aging | -24693483.5 |
| Low-middle SDI | -55285646.13 | Population | -24693483.5 |
| Low-middle SDI | -55285646.13 | Epidemiological change | -5898679.129 |
| Middle SDI | -29389028.46 | Aging | -14798398.24 |
| Middle SDI | -29389028.46 | Population | -14798398.24 |
| Middle SDI | -29389028.46 | Epidemiological change | 207768.027 |
| High-middle SDI | -1670441.06 | Aging | -967331.478 |
| High-middle SDI | -1670441.06 | Population | -967331.478 |
| High-middle SDI | -1670441.06 | Epidemiological change | 264221.9 |
| High SDI | -321697.85 | Aging | -180030.322 |
| High SDI | -321697.85 | Population | -180030.322 |
| High SDI | -321697.85 | Epidemiological change | 38362.792 |
| Global | 53135058.92 | Aging | -24263079.1 |
| Global | 53135058.92 | Population | 78517444.45 |
| Global | 53135058.92 | Epidemiological change | -1119306.438 |

**Table S9.** Changes in neglected tropical diseases and malaria-related prevalence number according to population-level determinants and causes from 1990 to 2021.

| location | Overll difference | varname | value |
| --- | --- | --- | --- |
| Low SDI | -321559672.9 | Aging | -132089373.6 |
| Low SDI | -321559672.9 | Population | -132089373.6 |
| Low SDI | -321559672.9 | Epidemiological change | -57380925.65 |
| Low-middle SDI | -537663182.4 | Aging | -211474522.7 |
| Low-middle SDI | -537663182.4 | Population | -211474522.7 |
| Low-middle SDI | -537663182.4 | Epidemiological change | -114714136.9 |
| Middle SDI | -626237928.8 | Aging | -240656340 |
| Middle SDI | -626237928.8 | Population | -240656340 |
| Middle SDI | -626237928.8 | Epidemiological change | -144925248.8 |
| High-middle SDI | -204706983.8 | Aging | -78741827.17 |
| High-middle SDI | -204706983.8 | Population | -78741827.17 |
| High-middle SDI | -204706983.8 | Epidemiological change | -47223329.47 |
| High SDI | -22989421.58 | Aging | -9817774.592 |
| High SDI | -22989421.58 | Population | -9817774.592 |
| High SDI | -22989421.58 | Epidemiological change | -3353872.397 |
| Global | -700326496.6 | Aging | -108327184.8 |
| Global | -700326496.6 | Population | 625113762.9 |
| Global | -700326496.6 | Epidemiological change | -1217113075 |

**Table S10.** Changes in neglected tropical diseases and malaria-related deaths number according to population-level determinants and causes from 1990 to 2021.

| location | Overll difference | varname | value |
| --- | --- | --- | --- |
| Low SDI | -184969.4 | Aging | -78295.119 |
| Low SDI | -184969.4 | Population | -78295.119 |
| Low SDI | -184969.4 | Epidemiological change | -28379.163 |
| Low-middle SDI | -118904.88 | Aging | -49745.091 |
| Low-middle SDI | -118904.88 | Population | -49745.091 |
| Low-middle SDI | -118904.88 | Epidemiological change | -19414.696 |
| Middle SDI | -61784.65 | Aging | -25741.909 |
| Middle SDI | -61784.65 | Population | -25741.909 |
| Middle SDI | -61784.65 | Epidemiological change | -10300.829 |
| High-middle SDI | -4914.88 | Aging | -2015.585 |
| High-middle SDI | -4914.88 | Population | -2015.585 |
| High-middle SDI | -4914.88 | Epidemiological change | -883.713 |
| High SDI | -536.51 | Aging | -246.447 |
| High SDI | -536.51 | Population | -246.447 |
| High SDI | -536.51 | Epidemiological change | -43.615 |
| Global | 34022.99 | Aging | 32005.214 |
| Global | 34022.99 | Population | 168078.67 |
| Global | 34022.99 | Epidemiological change | -166060.893 |

**Table S11.** Changes in neglected tropical diseases and malaria-related DALYs number according to population-level determinants and causes from 1990 to 2021.

| location | Overll difference | varname | value |
| --- | --- | --- | --- |
| Low SDI | -15258815.89 | Aging | -6234417.674 |
| Low SDI | -15258815.89 | Population | -6234417.674 |
| Low SDI | -15258815.89 | Epidemiological change | -2789980.546 |
| Low-middle SDI | -12135751.85 | Aging | -4887778.29 |
| Low-middle SDI | -12135751.85 | Population | -4887778.29 |
| Low-middle SDI | -12135751.85 | Epidemiological change | -2360195.269 |
| Middle SDI | -7873948.13 | Aging | -3204164.175 |
| Middle SDI | -7873948.13 | Population | -3204164.175 |
| Middle SDI | -7873948.13 | Epidemiological change | -1465619.78 |
| High-middle SDI | -1628818.2 | Aging | -667277.008 |
| High-middle SDI | -1628818.2 | Population | -667277.008 |
| High-middle SDI | -1628818.2 | Epidemiological change | -294264.183 |
| High SDI | -237351.07 | Aging | -110109.846 |
| High SDI | -237351.07 | Population | -110109.846 |
| High SDI | -237351.07 | Epidemiological change | -17131.375 |
| Global | -6062165.67 | Aging | -1028622.485 |
| Global | -6062165.67 | Population | 15113458.19 |
| Global | -6062165.67 | Epidemiological change | -20147001.37 |
